# Supplementary material for: Online cognitive–behavioural therapy for traumatically bereaved people: study protocol for a randomised waitlist-controlled trial
Source: BMJ Open. 2020 Sep 3;10(9):e035050. doi: 10.1136/bmjopen-2019-035050 (PMC7473627; doi:10.1136/bmjopen-2019-035050)
Supplement: Supplementary data [file bmjopen-2019-035050supp001.pdf]

Supplementary File

Information for participants

Subject: Participation in study on the treatment of grief after a traffic accident

Dear sir/madam,

We ask you to take part in a scientific study. This participation is voluntary. In order to take part we do need your permission. You receive this information because you filled in a questionnaire some time ago, mapping the emotional consequences of the death of a loved one due to a traffic accident. You have indicated that you may be interested in taking part in a follow-up study exploring the effects of a treatment to learn to cope better with the death of your loved one.

Before you decide whether you want to take part in this study, you receive an explanation of what the study entails. Read this information carefully and ask the researcher to explain if you have questions.

1. General Information

This study is conducted by the University of Groningen, Utrecht University and Stichting Centrum '45. The study is financed by the Fonds Slachtofferhulp (Victim Support Fund). The study consists of participation in an online psychological treatment. The treatment is provided by therapists in various practices across the Netherlands. The medical-ethical review committee of the Universitair Medisch Centrum Groningen (Academic Medical Centre Groningen) has approved this study.

2. Purpose of the study

Those left behind after a traffic accident often indicate that the help provided does not sufficiently connect to their experiences. Therefore a treatment has been developed that is specifically intended for the partner, relatives and friends of someone who died due to a traffic accident. You can discuss with the therapist how you are coping with the loss and what the consequences of this loss are in your life. The aim of the treatment is to cope better with the loss. The treatment is part of a scientific study. The purpose of the study is to explore whether treatment leads to a reduction in emotional problems for those who lost someone due to a traffic accident.

3. Background of the study

During the treatment cognitive behavioural therapy will be used. Previous research has shown that cognitive behavioural therapy is the most effective treatment for reducing emotional problems after the loss of a loved one due to a natural cause (for instance illness). Cognitive behavioural therapy is mainly applied during individual sessions with a therapist (face-to-face treatment). Research has shown that cognitive behavioural therapy, offered via the internet (online treatment), also seems to be suitable for reducing problems after a natural death. More research is needed to find out whether this online version of cognitive behavioural therapy is also suitable for those who lost someone due to a traffic accident. The purpose of this study is to find out whether online cognitive behavioural therapy is accompanied by a reduction of emotional problems after the death of a loved one due to a traffic accident.

The online treatment is provided individually and consists of eight modules which you go through in twelve weeks. You will then have online contact with a therapist who will guide you during the treatment. In the Netherlands, a network of therapists has been trained in the online treatment of people

who lost someone due to a traffic accident. The therapists work at several treatment centres. The online treatment is offered by Therapieland. Therapieland is a provider of psychological care via the internet.

4. What participation entails

If you take part, this will take at least 20 weeks in total for you.

Screening

First we will determine whether you can take part. You will be asked to fill in a questionnaire. The questionnaire contains questions about emotional problems you may experience in response to the passing away of your loved one due to a traffic accident. Also, questions are asked about previous psychological help you may have received. The questionnaire is used to get a picture of the degree to which you experience emotional problems.

If your completed questionnaire shows that you experience relatively few emotional problems, you cannot take part in the study. You also cannot take part in the study if you have no access to the internet. If your answers show that the treatment offered is not suitable for you, an alternative treatment will be looked for in consultation with you.

It is possible that you filled out a questionnaire on this topic before. As problems can change over time, we ask you to fill in a questionnaire once more. In this way we get a picture of the problems you are experiencing at the moment.

Treatment

In order to be able to determine the effect of the treatment, participants are assigned to one of two groups. The first group will start with the online treatment as soon as possible after registration. The second group will start with the online treatment after a waiting period of 20 weeks. By adding a waitlist group it can be determined that a reduction of problems is actually the result of the treatment, and not of the passage of time. Which group you are assigned to will be determined by drawing lots. You and we do not have any influence on the draw. We will let you know when your treatment starts.

Measurements

Before the treatment can start, you will be asked to fill in a questionnaire. This questionnaire will focus on the problems you experience. We map these in order to be able to determine whether the treatment might help you.

The therapist who will guide your online treatment will be informed of the results of this questionnaire beforehand. In order to determine to what extent the treatment helps you, we ask you to fill in a questionnaire not only beforehand, but also once during the treatment, after the last treatment session, and 8 weeks after the treatment. People assigned to the waiting group will be asked to fill in an additional questionnaire after 12 weeks and 20 weeks of waiting. In this way it can be determined whether the treatment has been effective and what the short and long term effects of the treatment are. The filling in will take approximately 10 to 30 minutes per measurement.

5. What is expected from you

In order to ensure that the study runs smoothly it is important that you adhere to the following agreements. These agreements are that you:

- contact the researcher in case of problems (the contact details are listed at the end of the information letter);
- keep all appointments with your therapist;
- fill in the questionnaires before and after the treatment.

Besides, it is important that you contact the researcher if:

- your contact information changes
- you no longer wish to take part in the study

6. Possible negative effects

During the treatment you will actively engage with your thoughts and feelings about the loss. It is possible that feelings such as grief or loss or fatigue may temporarily increase in intensity. Filling in the questionnaire can also evoke emotional responses. When your problems increase to a great extent, you can contact the researcher via the contact details listed at the end of this letter.

7. Possible advantages and disadvantages

It is important that you carefully weigh the possible advantages and disadvantages before you decide to take part. The treatment may reduce your emotional problems, but this is not certain. Your participation will contribute to more knowledge about the treatment of emotional problems after the death of a loved one due to a traffic accident.

Disadvantages of taking part in the study may be:

- possible worsening of problems due to taking part in the treatment;
- possible worsening of problems due to filling in the questionnaire.

Participation in the study also means that:

- the study will cost you time;
- you will have to adhere to certain agreements.

These issues have all been described in section 4, 5 and 6 above.

8. If you do not wish to participate or if you wish to stop participating in the study

You decide whether or not to take part in the study. Participation is voluntary. If you do take part, you can always change your mind and stop participating after all, during the study as well. You do not have to give a reason for stopping. You do have to report this to the researcher immediately. The data collected up to that point will be used for the study.

If there is new information about the study that is important to you, the researcher will let you know this. We will then ask you if you continue to participate.

9. End of the study

Your participation in the study will end when: you will have filled in the questionnaire 8 weeks after the end of the treatment; you make the choice to stop participating; the researcher considers it better for you to stop participating; the government or the reviewing medical-ethical committee decide to end the study. The entire study will end when all participants are done. After processing all the data, the researcher will inform you of the most important outcomes of the study by means of a newsletter. You can indicate whether you wish to receive this newsletter at the end of the questionnaire.

10. **Use and storage of your data**

For this study your personal data will be collected, used and stored. This concerns data such as your name, address, date of birth and data related to your health. The collection, use and storage of your data is necessary to be able to answer the questions asked in this study and to publish the results. We ask your permission for the use of your data.

Confidentiality of your data

In order to protect your privacy, your data will get a code. Your name and other data which can identify you directly will be left out from this. Data can only be traced to you with the key to the code. The key to the code will remain safely stored at the local research institution. The data sent to eventual other parties involved only contain the code, but not your name or other data with which you can be identified. In reports and publications on the study the data cannot be traced to you either.

Access to your data for checks

Some people may get access to all your data at the research location, including the data without code. This is necessary to be able to check whether the study has been done properly and reliably. People who will gain access to you data for checking purposes will be the researchers Lonneke Lenferink, a research assistant and Jos de Keijser, the committee monitoring the safety of the study and international supervising authorities. They will keep your data secret. We ask you to give your permission for this access.

Data retention period

Your data must be retained at the research location for 15 years. They are stored in order to be able to make new provisions related to this study in the course of this study.

Withdrawal of permission

You can always withdraw your permission for the use of your personal data again. The research data collected up to the moment you withdraw permission will still be used in the study.

Further information about your rights regarding data processing

For general information about you rights you can contact the person responsible for processing your personal data. For this study this is Lonneke Lenferink (University of Groningen). In case of questions or complaints regarding the processing of your personal data we recommend that you contact her.

11. **Insurance for test subjects**

An insurance has been taken out for everyone taking part in the study. The insurance covers damage from the study. You can report damage to the researchers.

12. **Informing family doctor/GP**

We do not share information about your participation in the study with your family doctor/GP.

13. **Compensation for participation**

Participation in the online treatment is free of charge.

14. Do you have any questions?

In case of general questions regarding the study you can contact the researcher assistant. If you have complaints about the study, you can discuss this with the researchers or with your treating therapist. If you prefer not to do this, you can contact the University of Groningen.

15. Signing the consent form

Below you find a 'Declaration of consent for participation in research into the treatment of grief after a traffic accident', on which you can indicate whether you wish to take part in the study. With your permission you indicate that you have understood the information and that you agree to participate in the study.

After you have filled in this declaration, you can start filling in the questionnaire. Approximately within four weeks after filling in the questionnaire we will let you know by email or by phone whether you qualify for the treatment. When you qualify for treatment, you will receive the outcome of the draw which indicates whether you have been assigned to a group that can start with the online treatment as soon as possible, or whether you have been assigned to the group that needs to wait for 20 weeks before the start of the online treatment.

If you have any further questions, you can contact us via the details below.

Kind regards,

[name research assistant]

Research assistant University of Groningen

Lonneke Lenferink

Post-doctoral researcher University of Groningen and Utrecht University

Contact Details

General questions about the study: [name research assistant], info@rouwnaverkeersongeval.nl

Questions regarding the protection of your data, your rights or complaints: please contact Lonneke Lenferink, l.i.m.lenferink@rug.nl.

Consent Form
for participation in research on treatment of grief after a traffic accident

- I have read the information letter. I also had the opportunity to ask questions. My questions have been answered adequately. I had enough time to decide whether to participate or not.
- I know that participation is voluntary. I also know that I can decide at any moment not to participate after all or to stop participating in the study. I do not have to give a reason for this.
- I give permission for collecting and using my data to answer the research question of this study.
- I know that some people may gain access to all of my data for the purpose of checks of this study. Those people are listed in this information letter. I give permission for this access by these people.
- I wish to participate in this study.

Name of participant:

Email address:

Phone number:

Signature of participant: Date: __ / __ / __

The researcher, Lonneke Lenferink, hereby declares that the test subject has been fully informed about the aforementioned study.

If information becomes known during the study which might influence the consent of the participant, the participant will be timely informed about this.
