# Supplementary material for: Online cognitive–behavioural therapy for traumatically bereaved people: study protocol for a randomised waitlist-controlled trial
Source: BMJ Open. 2020 Sep 3;10(9):e035050. doi: 10.1136/bmjopen-2019-035050 (PMC7473627; doi:10.1136/bmjopen-2019-035050)
Supplement: Author's manuscript [file bmjopen-2019-035050.draft_revisions.pdf]

BMJ Open is committed to open peer review. As part of this commitment we make the peer review history of every article we publish publicly available.

When an article is published we post the peer reviewers' comments and the authors' responses online. We also post the versions of the paper that were used during peer review. These are the versions that the peer review comments apply to.

The versions of the paper that follow are the versions that were submitted during the peer review process. They are not the versions of record or the final published versions. They should not be cited or distributed as the published version of this manuscript.

BMJ Open is an open access journal and the full, final, typeset and author-corrected version of record of the manuscript is available on our site with no access controls, subscription charges or pay-per-view fees (http://bmjopen.bmj.com).

If you have any questions on BMJ Open's open peer review process please email info.bmjopen@bmj.com

BMJ Open

Face-to-face and online cognitive behavioral therapy for traumatically bereaved people: Study protocol for a randomized waitlist controlled trial

Journal:	*BMJ Open*
Manuscript ID	bmjopen-2019-035050
Article Type:	Protocol
Date Submitted by the Author:	16-Oct-2019
Complete List of Authors:	Lenferink, Lonneke; Rijksuniversiteit Groningen,<br>de Keijser, Jos; Rijksuniversiteit Groningen<br>Eisma, Maarten<br>Smid, Geert<br>Boelen, Paul; Utrecht University
Keywords:	PSYCHIATRY, Adult psychiatry < PSYCHIATRY, PUBLIC HEALTH

SCHOLARONE™
Manuscripts

I, the Submitting Author has the right to grant and does grant on behalf of all authors of the Work (as defined in the below author licence), an exclusive licence and/or a non-exclusive licence for contributions from authors who are: i) UK Crown employees; ii) where BMJ has agreed a CC-BY licence shall apply, and/or iii) in accordance with the terms applicable for US Federal Government officers or employees acting as part of their official duties; on a worldwide, perpetual, irrevocable, royalty-free basis to BMJ Publishing Group Ltd ("BMJ") its licensees and where the relevant Journal is co-owned by BMJ to the co-owners of the Journal, to publish the Work in this journal and any other BMJ products and to exploit all rights, as set out in our licence.

The Submitting Author accepts and understands that any supply made under these terms is made by BMJ to the Submitting Author unless you are acting as an employee on behalf of your employer or a postgraduate student of an affiliated institution which is paying any applicable article publishing charge ("APC") for Open Access articles. Where the Submitting Author wishes to make the Work available on an Open Access basis (and intends to pay the relevant APC), the terms of reuse of such Open Access shall be governed by a Creative Commons licence – details of these licences and which Creative Commons licence will apply to this Work are set out in our licence referred to above.

Other than as permitted in any relevant BMJ Author's Self Archiving Policies, I confirm this Work has not been accepted for publication elsewhere, is not being considered for publication elsewhere and does not duplicate material already published. I confirm all authors consent to publication of this Work and authorise the granting of this licence.

For peer review only

Face-to-face and online cognitive behavioral therapy for traumatically bereaved people: Study protocol for a randomized waitlist controlled trial

Lenferink, L.I.M.*[1,2], de Keijser, J.[1], Eisma, M.C.[1], Smid, G.E.[3,4,5], & Boelen, P.A.[2,3,4]

[1] Department of Clinical Psychology and Experimental Psychopathology, Faculty of Behavioral and Social Sciences, University of Groningen, Grote Kruisstraat 2/1, 9712 TS Groningen, The Netherlands. E-mail L.I.M. Lenferink: l.i.m.lenferink@rug.nl.

[2] Department of Clinical Psychology, Faculty of Social Sciences, Utrecht University, P.O. Box 80140, 3508 TC, Utrecht, The Netherlands.

[3] ARQ National Psychotrauma Centre, Nienoord 5, 1112 XE, Diemen, The Netherlands

[4] Foundation Centrum '45, Nienoord 5, 1112 XE Diemen, The Netherlands

[5] University of Humanistic Studies, Utrecht, The Netherlands

*Corresponding author

For peer review only - http://bmjopen.bmj.com/site/about/guidelines.xhtml

Abstract

Introduction

The sudden or violent death of a significant other, such as death due to a traffic accident, can precipitate persistent complex bereavement disorder (PCBD) and comorbid posttraumatic stress disorder (PTSD) and depression. Waitlist controlled trials have shown that grief-specific cognitive behavioral therapy (CBT), delivered face-to-face or online, is an effective treatment for mental health problems. This is the first study that will examine 1) the effectiveness of CBT in a sample exclusively comprised of people bereaved by a traumatic death and 2) the effectiveness of face-to-face CBT (vs. waitlist controls) *and* online CBT (vs. waitlist controls). Our primary hypothesis is that people allocated to the face-to-face or online CBT condition will show larger reductions in PCBD, PTSD, and depression symptom levels post-treatment than people allocated to a waitlist. We further expect that treatment effects are mediated by reductions of negative cognitions and avoidance behaviors and moderated by accident-related stressors. Lastly, the associations between therapeutic alliance and treatment outcomes will be explored.

Methods and analysis

A three-arm (face-to-face CBT, online CBT, and waiting list) open label parallel randomized controlled trial (RCT) will be conducted. Participants will complete questionnaires at pre-treatment and 12 and 20 weeks after study enrolment. Eligible for participation are Dutch adults who lost a family member, spouse, or friend at least one year earlier due to a traffic accident and report clinically relevant levels of PCBD, and/or PTSD, and/or depression. Multilevel modeling will be used for the main analyses.

For peer review only - http://bmjopen.bmj.com/site/about/guidelines.xhtml

Ethics and dissemination

Ethical approval has been received (METc UMCG:2019.233). If the treatments are demonstrated to be effective, both treatments will be available for future bereaved people. Findings will be disseminated among lay people (e.g., newsletters), our collaborators (e.g., through presenting at non-governmental/(peer-)support organizations), and clinicians and researchers (e.g., (inter)national conferences/journals).

Keywords: bereavement; grief; trauma; PTSD; treatment; intervention.

Strengths and limitations of this study

- This study is the first to examine the effectiveness of face-to-face CBT (vs. waitlist controls) and online CBT (vs. waitlist controls) in reducing psychopathology after traumatic loss in a three-arm RCT.
- This treatment study is one of the first to examine potential moderators and mediators of change in symptom levels after traumatic loss.
- We are not able to statistically test if CBT offered face-to-face or online has equal effects, because limited recourses do not allow for recruitment of very large samples.
- Another limitation is the use of self-report questionnaires, instead of diagnostic interviews, to assess symptom levels.

Trial registration number

NL7497 (Dutch Trial Register)

Word Count: 5,017 words including main text

For peer review only - http://bmjopen.bmj.com/site/about/guidelines.xhtml

Introduction

Worldwide, traffic accidents represent the leading cause of unnatural death[1]. Ten to 20% of bereaved people develop severe and persistent grief-related distress, including persistent complex bereavement disorder (PCBD), posttraumatic stress disorder (PTSD), and depression after natural deaths (e.g., illness)[2,3]. Notably, PCBD has been introduced, as other specified psychotrauma- and stressor-related disorder, in the latest version of the Diagnostic and Statistical manual of Mental Diseases (DSM-5)[4]. PCBD is diagnosed if, after the death of a significant other at least 12 months earlier, the person experiences persistent yearning for the deceased and symptoms of reactive distress (e.g., emotional numbness) and social/identity disruption (e.g., feeling alone) causing impairment in daily life. While some PCBD symptoms overlap with PTSD (e.g., anger) and depression symptoms (e.g., sadness), several studies have shown that these three syndromes are distinct[5–7].

Unexpected/violent losses of a significant other, also referred to as a traumatic losses, including deaths caused by traffic accidents, render a risk for developing PCBD, PTSD, and depression[8,9]. The co-occurrence of (symptoms of) these disorders following traumatic loss has also been referred to as “traumatic grief”[10].

Increased risk for developing psychopathology after deaths due to traffic accident

Specific circumstances of losses caused by accidents may account for the elevated risk of traumatic grief. For instance, experiencing multiple losses simultaneously, being a witness to the accident, and juridical and financial consequences are presumed to exacerbate grief-related distress[11]. Furthermore, negative cognitions and avoidance behaviors may mediate the impact of sudden/violent loss on grief, PTSD, and depression levels[12]. According to a cognitive-behavioral model of complicated grief, three interacting malleable

For peer review only - http://bmjopen.bmj.com/site/about/guidelines.xhtml

processes underlie disturbed grief reactions: i) negative cognitions, ii) avoidance behavior, and iii) difficulties integrating the loss into the autobiographical knowledge base[13].

Experiencing a loss due to a traffic accident may violate basic assumptions about the world being a safe place[14]. This may fuel negative cognitions (e.g., “I’m less worthy, since s/he died” and “The death of him/her has taught me that the world is unjust) that may exacerbate and maintain acute grief responses[15]. Avoidance behaviors include depressive avoidance and anxious avoidance strategies. Depressive avoidance refers to withdrawal from social and occupational activities that were perceived as fulfilling before the death, out of the conviction that these activities are no longer meaningful. Anxious avoidance strategies serve to prevent confrontation with the reality of the death, out of fear that confrontation is too painful[13]. One way to avoid confrontation with the reality of the loss, is to focus on angry thoughts and feelings (e.g., “I was angry at the police, courts, or administration, because they did not do their work well enough“)[16]. This seems to be a frequently used avoidant coping strategy in bereaved people after traffic accidents and is strongly related to PTSD[17]. Difficulty with integration of the loss into the autobiographical knowledge base refers to the difficulties connecting factual knowledge that the loss is irreversible with existing information about the self and the relationship with the lost person, stored in autobiographical memory. Memories related to the loss may lack context in terms of time and place, causing the loss to be experienced as unreal[18]. It has been argued that this “sense of unrealness” may trigger intrusive memories and increase feelings of numbness or shock once the bereaved person is confronted with reminders of the loss[18,19]. The extent to which a person believes that one is capable of managing stressor-related thoughts, emotions, and behaviors, also referred to as self-efficacy (e.g., “I can usually handle whatever comes my way“), has also been determined as an important factor facilitating

For peer review only - http://bmjopen.bmj.com/site/about/guidelines.xhtml

coping with traumatic stressors[20]. Decreased self-efficacy, negative cognitions and insufficient integration of the loss may contribute to increased sensitivity to loss reminders or secondary stressors following traumatic loss[10].

Face-to-face cognitive-behavioral therapy for grief-related distress

Grief-specific CBT has, so far, proven to be the most effective treatment for bereaved people with elevated grief levels[21–24]. CBT targets the above-mentioned cognitive-behavioral variables with cognitive restructuring, loss-related exposure, and behavioral activation. Notably, research on putative mechanisms of change of grief-specific CBT is sparse[23] (but see[25,26]). Focusing on people with traumatic grief is relevant because it would enable tailoring of interventions to the specific needs of this group, which could improve treatment outcomes[27].

Studies evaluating the effectiveness of CBT-based interventions for people bereaved by sudden/violent deaths have been conducted earlier, but these were often designed to target high risk groups irrespective of symptom levels (e.g., people bereaved by suicide[27]; see also:[24]). Since interventions for people without mental health complaints following loss do not appear to be effective[28], it is critical to specifically examine the effectiveness of CBT in groups who experience traumatic grief. While some studies did so, these tested the effectiveness of CBT with additional interventions, such as group interventions or eye movement desensitization and reprocessing[29,30]. It is therefore yet unclear if application of CBT in and of itself is effective in reducing psychopathology in people with traumatic grief.

Face-to-face and online cognitive behavioral therapy for grief-related distress

For peer review only - http://bmjopen.bmj.com/site/about/guidelines.xhtml

Whilst the majority of trials assess the efficacy of face-to-face CBT[24], some online CBT-based interventions have been developed for distressed bereaved people[31–33]. Offering CBT through the internet has some potential advantages. It may lower the threshold for seeking treatment, because it can be delivered independent of geographical location. Furthermore, asynchronous communication may be used, allowing the client and therapist can contact each other at any preferred time[34]. This may counter barriers to mental health service use, such as difficulties with finding help, transportation concerns, or difficulties scheduling treatment sessions[35]. In addition, online CBT could reduce treatment costs, improving its accessibility and dissemination for people in need of support[36].

A potential downside to online CBT is the high dropout rates found in earlier studies[36,37]. It has been argued that a strong therapeutic alliance might support adherence to online treatment and mediates treatment effects [38]. Therapeutic alliance is defined as a positive emotional bond between client and therapist, whereby both parties agree on the tasks and goals of the treatment[39]. A good client-therapist relationship might also explain why guided are more effective than unguided online treatments[34]. Concerns have been raised that developing a therapeutic relationship might be more difficult when non-verbal communication is absent[40]. Studies in non-bereaved samples indicate that developing a strong therapeutic alliance is possible during online treatment[36], and this has been found to be related to online treatment outcomes[41], but see Andersson and Titov (2014). More research is needed to further examine the potential differences in quality of client-therapist relationship between face-to-face and online CBT and its relationship with treatment outcomes.

Study objectives

For peer review only - http://bmjopen.bmj.com/site/about/guidelines.xhtml

Our first aim is to examine the effectiveness of face-to-face CBT (vs. a waiting list control condition) and online CBT (vs. a waiting list control condition) in reducing symptom levels of PCBD, PTSD, and depression in people bereaved by a traffic accident. We expect that participants assigned to the face-to-face and the online CBT conditions will show larger reductions in symptom levels of PCBD, PTSD, and depression compared with waitlist controls at post-treatment assessments (Hypothesis 1).

Our second aim is to explore potential mediators of therapeutic effects. Based on prior research and theorizing[13,17,20], we expect that reductions in negative cognitions, avoidance behaviors, state anger, a sense of unrealness, and improvement in self-efficacy mediate the positive effects of face-to-face (vs. waiting list controls) and online CBT (vs. waiting list controls) (Hypothesis 2a). In addition, our aim is to explore whether background characteristics (i.e., gender, age, and educational level, kinship to the deceased, and time since loss) and accident-related stressors (i.e., single vs. multiple loss, witnessed the accident, and involvement in legal trial) moderate treatment effects (Hypothesis 2b). We have no specific expectations regarding these associations because prior treatment studies in bereaved people showed inconsistent results[24,25,30]. However, based on clinical experience, we expect that accident-related stressors will moderate treatment effects, such that multiple loss, witnessing the accident, and involvement in legal trial negatively impact treatment effects.

Our third aim is to explore i) potential differences between the face-to-face and online CBT condition in therapeutic alliance (as perceived by the participant and therapist) and (ii) the associations between quality of the therapeutic alliance and treatment

For peer review only - http://bmjopen.bmj.com/site/about/guidelines.xhtml

outcomes. Because prior research findings in this area are not equivocal[36,41], no specific hypotheses were formulated.

For peer review only

For peer review only - http://bmjopen.bmj.com/site/about/guidelines.xhtml

Methods and analysis

Design

A three-arm (face-to-face CBT, online CBT, and waiting list) multi-centre open label parallel RCT will be conducted in the Netherlands. Randomization will take place after the participant is screened for eligibility-based inclusion criteria (described below). A random number generator (www.random.org) will be used by a blinded independent researcher, to perform the blocking randomization procedure. An allocation ratio of 1:1:1 will be applied.

Participants allocated to the face-to-face or online CBT condition receive treatment within one week after allocation. All participants will be asked to fill in questionnaires (described below) at baseline (T1), 12 weeks post-allocation (T2), and 20 weeks post-allocation (T3). For participants in the waiting list control group, T3 is at the end of the 20-week waiting period after which they will be randomly allocated to receive face-to-face or online CBT (see Figure 1). A waiting list control group (instead of a no treatment control group) is included to increase the likelihood of participation by guaranteeing that all participants receive treatment. Furthermore, the inclusion of a waiting list control group allows a treatment versus no treatment comparison, that will provide knowledge about the effects of treatment relative to natural recovery from loss. We chose to randomly allocate waitlist controls to face-to-face or online CBT after the 20-week waiting period to maximize the sample size needed for the third research aim.

In line with prior treatment studies from our research group[30,42], the face-to-face CBT is carried out at the institution or private practices of licensed and registered psychologists who are a member of our Traumatic Loss Network (i.e., informal national network of trauma and grief therapists that are involved in research projects of our research

For peer review only - http://bmjopen.bmj.com/site/about/guidelines.xhtml

group). All psychologists will receive an 8-hour training about the treatment protocol of this intervention study. The personalized therapist feedback in the online CBT will also be provided by members of the Traumatic Loss Network. The treatment costs in both conditions will be reimbursed.

Figure 1. RCT design

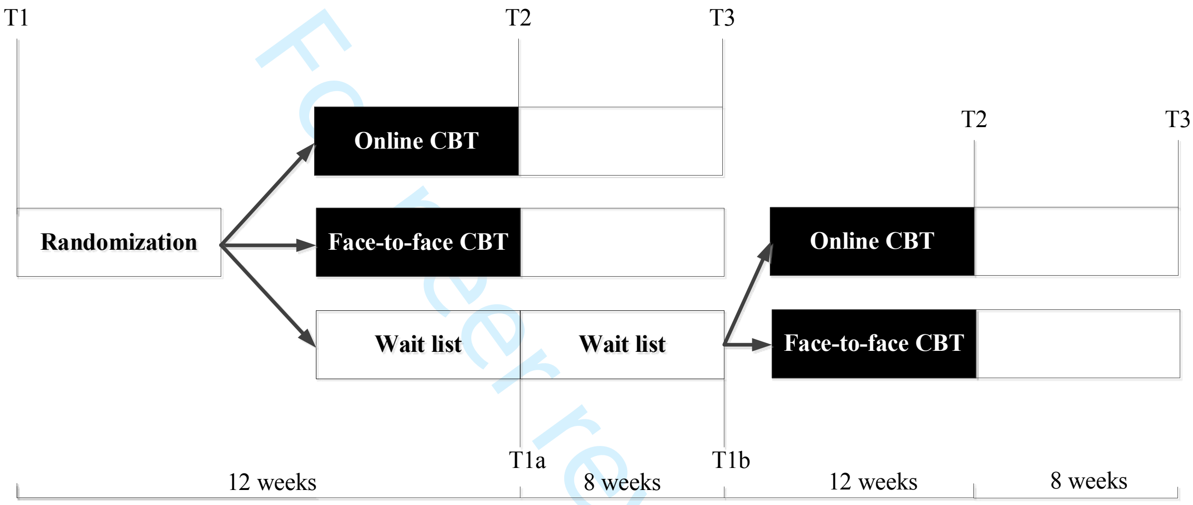

Note. CBT = cognitive-behavioral therapy

Participants

This RCT is part of a larger on-going research project (so termed "TrafVic-project") examining the psychological impact of, and care after, the death of a significant other due to a traffic accident. We expect to recruit the majority of the participants via a survey-study that has started in December 2018 that included the following question: "In this study we would like to offer psychological help to persons who experience emotional problems. May we approach you with more information about this offer, if your answers to this questionnaire imply that you may experience emotional problems?" People who answered 'yes' will receive a letter with information about the intervention, the treatment study, and an informed consent form. A website (www.rouwnaverkeersongeval.nl) has been developed so

For peer review only - http://bmjopen.bmj.com/site/about/guidelines.xhtml

that potential participants can read information about the research and treatments. People who are interested can also sign up for the study via this website. Recruitment for this RCT had not started at the time of submission of this manuscript.

To be eligible for study participation, the person must 1) be a family member, spouse, or friend of a person who died due to a traffic accident at least one year earlier, 2) be ≥18 years of age, and 3) meet DSM-5 criteria for PCBD and/or PTSD and/or experience clinically relevant depression, based on questionnaire scores (see below for more details). People are excluded when they: 1) do not master the Dutch language, 2) do not have access to Internet, 3) suffer from a substance use disorder, psychotic disorder, intellectual disability, and/or suicidality based on clinical judgment at the intake.

Sample size

To test our primary hypothesis (Hypothesis 1), two tests for each outcome separately (PCBD, PTSD, and depression) will be conducted, with test 1 examining face-to-face CBT vs. waitlist controls and test 2 examining online CBT vs. waitlist controls. To find a difference between two groups (face-to-face CBT vs. waitlist controls and online CBT vs. waitlist controls) of at least a medium effect size (f = 0.25; based on prior research[22,30,31]) with a power of 80%, an α of 0.017 (corrected for multiple testing, i.e., 0.05/3, because of three primary outcome measures (PCBD, PTSD, and depression)), and a strong association (r = .50) between the three repeated measures (T1, T2, and T3), a sample size of 23 per condition is sufficient. Taking into account an average dropout rate of 19% [22], a total sample size of 82 (69+13) is required to test Hypothesis 1.

Because our data are nested (repeated measures) (level 1) within individuals (level 2), and possibly within families sharing the same household (level 3), multi-level modeling

For peer review only - http://bmjopen.bmj.com/site/about/guidelines.xhtml

will be performed to test hypothesis 1. Conducting a power analysis within a multi-level framework is not feasible for various reasons[42]. We therefore conducted the power analysis for a repeated measures ANOVA.

Intervention

CBT will consist of eight sessions offered in within a timeframe of 12 weeks. Eight sessions have shown to be sufficient to yield clinically relevant effects in prior research[30]. Following Dutch guidelines for grief-specific CBT[43], central components of the treatment are exposure, cognitive restructuring, and behavioral activation. In the first session, psychoeducation is offered, including information about possible emotional reactions to the death of a loved one in a traffic accident and processes that might foster or hamper recovery. A rationale for the CBT interventions is provided.

Then, session 2, 3, and 4 are focused on exposure; the circumstances and story of the loss are discussed in detail, and the participant is encouraged to confront stimuli that s/he tends to avoid. Exposure is conducted by imaginary exposure assignments in session and after the session by writing assignment that have proven effective in prior research[33]. These writing assignments are focused on writing a detailed narrative of the loss and its circumstances.

The next sessions (5 and 6) focus on identifying and changing negative cognitions that hamper adjustment (i.e., cognitive restructuring); specific attention is paid to cognitions connected with responsibility/guilt and anger that may be elevated following the accidental death[11]. Cognitive restructuring assignments are provided to gain an alternative perspective on negative thoughts about the self, life, the future, by 1) psycho-education about common unhelpful thoughts, 2) identifying one's own unhelpful thoughts, and 3)

For peer review only - http://bmjopen.bmj.com/site/about/guidelines.xhtml

For peer review only

challenging these thoughts. Participants are instructed to undertake these three steps by writing down each day a description of i) an emotional moment/event, ii) their thoughts during this event, iii) their feelings (and intensity of these feelings on a scale of 1 through 10), iv) their behavior, v) evaluation of their thoughts), and vi) alternative helpful thoughts.

In session 7 and 8, participants are encouraged to reengage in previously valued social, recreational, and occupational activities in order to facilitate the process of adjustment. Behavioral activation assignments are focused on writing about valued activities and making plans to achieve valued goals. Session 8 is also focused on what the participant has learned and how to deal with difficulties in the future.

Online CBT uses the same interventions with the same goals, yet with all information and assignments being presented in an online framework, offered via a secure website. As in the face-to-face CBT, exposure, cognitive restructuring, and behavioral activation are central. Instead of information being exchanged interactively between the therapist and the participant, in online CBT, participants listen to a video-therapist verbally sharing information. Similar to face-to-face CBT, the treatment includes 8 sessions, called lessons. Participants receive weekly asynchronous written feedback from an online therapist on assignments that they complete. In both conditions, participants receive written information — offered in a treatment manual for face-to-face condition and offered online for the online condition — that consists of psychoeducation, details about the content of the treatment, and homework assignments. Moreover, in both conditions, participants are encouraged to ask a significant other to support them during treatment. This support figure is then informed about the treatment through written information.

For peer review only - http://bmjopen.bmj.com/site/about/guidelines.xhtml
For peer review only

Treatment adherence for the face-to-face CBT will be monitored by asking the therapists to report compliances and deviations from the protocol in a journal. Adherence for the online CBT will be monitored by evaluating log data. Monthly supervision of the therapists takes place by telephone.

Measures

Primary outcome measures

PCBD is assessed with the Traumatic Grief Inventory-Self Report (TGI-SR)(44). The TGI-SR consists of 18 items on a 5-point Likert scale ranging from 1 = never through 5 = always. Four items tapping disturbed grief criteria according to the 11th edition of the International Classification of Diseases were added(45). An example of an item is: "I found it difficult to trust others". The instruction of the original questionnaire was altered from referring to "the death of your loved one" to "the death of your loved one(s) due to a traffic accident". Psychometric properties of the TGI-SR are adequate(44,46). Participants are considered as meeting criteria for DSM-5 PCBD(4) when they score at least 3 ("Sometimes") on at least 1 criterion B symptom (Item 1, item 2, item 3, and item 14), at least 6 criterion C symptoms (item 4 up to 11, and item 15 up to 18), and the criterion D symptom (item 13).

PTSD symptoms are assessed with the PTSD Checklist for DSM-5 (PCL-5)(47) (Dutch version: (48)). Participants rate how often they were bothered by each symptom (e.g., "In the past month, how much were you bothered by trouble remembering important parts of the accident?") on 5-point Likert scales (0 = not at all and 4 = extremely). The instruction and the items of the original questionnaire are altered from referring to the "stressful event" to the "the death of your loved one(s) due to a traffic accident". The PCL-5 has shown to be reliable and valid(47). Participants meet the criteria for DSM-5 PTSD(4) when they score at

For peer review only - http://bmjopen.bmj.com/site/about/guidelines.xhtml

least 2 ("Moderately") on 1 criterion B item (items 1-5), 1 criterion C item (items 6-7), 2 criterion D items (items 8-14), and 2 criterion E items (items 15-20).

Depression symptom are assessed with the depression subscale of the HADS-D(49). The HADS-D consists of 7 items (e.g., “I still enjoy the thing I used to do”) rated on 4-point scores ranging from 0 (e.g., “Hardly at all”) through 3 (e.g., “Definitely as much”). The Dutch HADS-D is a reliable and valid screening tool for depression(50). A cut-off score of ≥8 is used as indicator for clinically relevant depression(49).

Secondary outcome measures

Impairments in daily functioning is measured with the 5-item Work and Social Adjustment Scale (WSAS)[51] (Dutch version:[52]). Participants rate, on 9-point Likert scales (0 = not at all to 8 = extremely), how much they are currently impaired in, for instance work, because of the death of their loved one(s) due to a traffic accident. The WSAS demonstrated good reliability and validity[51].

Negative grief-related cognitions are assessed with 18 items from the Grief Cognitions Questionnaire (GCQ)[15]. Participants are asked to rate their agreement with each item (e.g., “Since [–] is dead, I feel less worthy”) on 6-point scales varying from 0 = disagree strongly through 5 = agree strongly. The psychometric properties have been positively evaluated in prior research[15].

Avoidance is measured with the Depressive and Anxious Avoidance in Prolonged Grief Questionnaire (DAAPGQ)[53]. The depressive avoidance subscale consists of 5 items (e.g. ‘Since [–] is dead, I do much less of the things that I used to enjoy.’) and the anxious avoidance subscale consists of 4 items (e.g., ‘I avoid to dwell on painful thoughts and

For peer review only - http://bmjopen.bmj.com/site/about/guidelines.xhtml

memories connected to his/her death.'). Participants answer each item on an 8-point scale with 0 = not at all true for me, and 7 = completely true for me. The DAAPGQ has adequate psychometric properties[53].

State anger is assessed with the 15-item state anger subscale of the State-Trait Anger Expression Inventory-2 (STAXI-2)[54] (Dutch version:[55]). Participants are asked to rate on 4-point Likert scales (1 = not at all and 4 = extremely) how angry they feel right now (e.g., "I feel annoyed"). The STAXI-2 is a valid and reliable measure to assess state anger[55].

A sense of unrealness is measured with the 5-item Experienced Unrealness Scale[18]. Participants are asked to rate their agreement with each item (e.g., "I still can hardly imagine that [–] will never be here again") on 8-point scales (0 = not at all true for me 7 = completely true for me). This instrument demonstrated adequate psychometric properties[18].

Self-efficacy is assessed with the General Self-Efficacy Scale (GSES)[56]. The GSES is a 10-item measure. Participants are asked to rate their agreement with each item (e.g., "I can solve most problems if I invest the necessary effort.") on a 4-point scales (1 = completely not true, 4 = completely true). The GSES has shown excellent reliability and validity[56].

Quality of the therapeutic alliance is measured with the 12-item Work Alliance Inventory-Short Form, Client Version and Therapist Version at session 4 (WAI-SF)[57] (Dutch version:[58]. The WAI-SF consists of 12 items (e.g., Client version: "We agree on what is important for me to work on", Therapist Version: "We are working towards mutually agreed upon goals.") on 5-point scales (1 = never and 5 = always). Higher total scores indicate a higher quality of the therapeutic alliance as perceived by the participant and therapist. The WAI-SF is a reliable and valid assessment tool[59].

For peer review only - http://bmjopen.bmj.com/site/about/guidelines.xhtml

Other measures

Participants are allowed to receive other forms of psychosocial support during participation in the trial. Based on a single question we will assess whether the participants received other forms of psychosocial professional support in order to consider this in our analyses. The following question will be used: “During the past 12 weeks/8 weeks (for T2 and T3, respectively) did you receive additional psychological professional support from a psychologist, therapist or psychiatrist other than the (on-line) therapist from the TrafVic-study?” Other forms of support (for example instrumental and legal support) and the use of (psychotropic) medications are also allowed for every participant.

Statistical analyses

To examine the differences in reductions of symptom levels of PCBD, PTSD, and depression from pre- to post-treatment between the conditions (face-to-face CBT, online CBT, and waitlist), six independent multi-level models will be built (Hypothesis 1). Symptom levels of PCBD, PTSD, and depression will consecutively be included as dependent variables and condition (face-to-face CBT vs waitlist controls or online CBT vs. waitlist controls), time, and time x condition (interaction term) as predictor variables, taking into account that repeated observations (level 1) are nested within individuals (level 2), and within households (level 3; if applicable). Additionally, relevant background, loss-related variables, and use of co-interventions (yes/no) during participation in our study, will be included in the analysis as covariates. Deviance tests will be used to examine whether inclusion of these covariates improves model fit[60].

To test hypothesis 2a, mediation analyses will be conducted for the dependent variables that show a significant time x condition interaction effect. Each possible mediator

For peer review only - http://bmjopen.bmj.com/site/about/guidelines.xhtml

(i.e., negative cognitions, avoidance behavior, state anger, a sense of unrealness, and self-efficacy) will be included in the model separately. Multiple mediation models will not be examined, due to an anticipated lack of power. Scores at three measurement occasions (T1, T2, and T3 for the face-to-face and online CBT and T1, T1a, and T1b for the wait list controls) for the mediator and dependent variable will be taken into account in one structural equation model following recommendations from Little[61].

To achieve research aim 2b, multilevel analyses will be used to examine to what extent treatment effects on PCBD, PTSD, and depression levels are moderated by background characteristics, including gender (man vs. woman), age (in years), and educational level (low vs. high), kinship to the deceased (child/spouse vs. other), and time since loss (in years), and b) accident-related stressors, including number of losses (single vs. multiple), witnessing the accident (no vs. yes), and involvement in legal trial (no vs. yes). Each possible moderator will be added to the model by including it as a predictor and as interaction term (e.g., time x condition x gender).

For examination of the third research aim, T-tests will be used to explore to what extent therapeutic alliance (total and subscale sores) differ between conditions (face-to-face vs. online CBT). Lastly, therapeutic alliance scores will be added as predictor in multilevel models to examine to what extent it interacts (i.e., time x condition x therapeutic alliance) with treatment outcomes. Data of all participants entering the study will be included in all analyses (i.e., intention-to-treat analysis).

Ethics and dissemination

This study has been approved by a local ethics committee (METc UMCG: ID number: 2019.233). The study will be conducted according to the principles of the Declaration of

For peer review only - http://bmjopen.bmj.com/site/about/guidelines.xhtml

Helsinki (8th version, 2013) and in accordance with the Medical Research Involving Human Subjects Act. Collected data will be handled confidentially, according to the EU General Data Protection Regulation and the Dutch Act on Implementation of the General Data Protection Regulation. Unidentifiable data from this trial will be stored in data repositories from the University of Groningen and Utrecht University.

Findings of this RCT will be disseminated among participants by means of a newsletter. If shown to be effective, the treatment will be accessible for future bereaved people because, as a result of this study, 1) a nationwide network of therapists are trained in our protocol for the face-to-face treatment, and 2) because the online framework will be made publicly accessible. Findings will also be disseminated among lay people by uploading the newsletters on our website (www.rouwnaverkeersongeval.nl) and through media performances. Our findings will be presented to our collaborators, including non-governmental organizations and (peer-)support organizations for bereaved people. Treatment materials are available online for free (see www.rouwnaverkeersongeval.nl/downloads). Lastly, colleagues will be informed about our findings during presentations at (inter)national conferences and by articles in scientific journals.

For peer review only

Discussion

The relatively few RCTs among general bereaved people with elevated grief levels indicate that grief-specific CBT-based interventions delivered face-to-face or online yield the largest effects on post-loss mental health compared with a waiting list[21–24]. RCTs evaluating treatment effects for people with elevated mental health complaints after confrontation with sudden/violent losses are lacking, with one notable exception that compared EMDR plus CBT vs. wait list controls[30]. Given that traumatically bereaved people are most strongly at risk for PCBD and comorbid PTSD and depression[8], it seems particularly relevant to develop evidence-based interventions for this population.

This will be the first RCT to examine the effectiveness of CBT in a sample exclusively comprised of people exposed to a traumatic death. Furthermore, the effectiveness of face-to-face *and* online grief-specific CBT (vs. waitlist controls) will be examined for the first time in one trial. We are not able to test whether face-to-face and online CBT has equal effects. A non-inferiority trial would require a sample size of over 1000 people[62], which is not feasible given our resources. Nonetheless, the findings are expected to yield important insights in the effects of online CBT (vs. waitlist controls) relative to the effects of face-to-face CBT (vs. waitlist controls). Unlike prior studies, our study design enables a fair, albeit indirect comparison of face-to-face and online grief treatment. For instance, in this RCT the treatment conditions are designed to be as similar as possible in terms of treatment content, treatment duration, experience and training of therapists, outcome measures, and characteristics of study participants. When we find similar effect sizes for between group comparisons (face-to-face CBT vs. waiting list and online CBT vs. waiting list), CBT delivered online can be considered as supplement for or complementary to face-to-face treatment,

For peer review only - http://bmjopen.bmj.com/site/about/guidelines.xhtml

because it potentially overcomes drawbacks of face-to-face treatment, such as waiting lists and travel expenses.

We will also examine potential mediators and moderators of change. Findings from mediation analyses, examining the role of among others negative cognitions and avoidance behaviors, will provide insights in potential underlying therapeutic processes to foster recovery from traumatic loss. These insights are deemed important to design treatments that more effectively target these mechanisms of change. Findings from the moderator analyses are expected to improve our knowledge on for whom (e.g., women or people who are more remotely bereaved) this grief-CBT works best. Findings on potential mediators and moderators of change are necessary to improve treatments given that a maximum of 42% of bereaved people report clinically relevant reductions in grief levels after treatment[21,23].

Lastly, the role of therapeutic alliance on therapy outcomes will be explored. Prior research in bereaved people has shown that greater therapeutic alliance, from the perspective of the client, at week 4 of a face-to-face grief-specific treatment, was related to greater reductions in grief levels. This therapeutic alliance-grief relationship was not significant for non-grief specific treatment[63]. Our exploration of this association, from the perspective of client *and* therapist, may for the first time shed light on (similarities or differences in) success rates of face-to-face and online CBT for traumatic grief.

An anticipated limitation of our RCT is the self-selected sample. It is possible that people who are more open towards innovative technology in general[64] and received support prior to the loss[35] are more likely to sign up for this study, limiting the generalization of findings emerging from this study. Furthermore, we will use self-report measures instead of diagnostic interviews, which may increase the risk of overestimating

For peer review only - http://bmjopen.bmj.com/site/about/guidelines.xhtml

symptom levels[65]. Another limitation of this trial may relate to the fact that the operationalization and assessment of grief as a disorder is still under debate[66–68]. For instance, PCBD, included as “condition for further study” in the DSM-5, is likely to be changed in a revision of the DSM. To maximize diagnostic compatibility, we added four items, corresponding to ICD-11 PGD criteria, to the TGI-SR, enabling operationalizion of our primary outcome measure in terms of diagnoses of pathological grief according to both DSM-5 and ICD-11.

To conclude, this RCT will provide new insights in effectiveness of face-to-face and online CBT for bereaved people after traffic accidents with clinically relevant distress as well as in mediators and moderators of therapeutic change. As trials to date have primarily focused on effects of face-to-face treatment for non-traumatically bereaved people, our findings are expected to provide a valuable addition to the knowledge base on treating severely distressed bereaved people.

For peer review only

For peer review only - http://bmjopen.bmj.com/site/about/guidelines.xhtml

References

1 World Health Organization. The top 10 causes of death [Internet]. 2019 [accessed on October 14 2019]. Available from: https://www.who.int/news-room/fact-sheets/detail/the-top-10-causes-of-death

2 Lundorff M, Holmgren H, Zachariae R, et al. Prevalence of prolonged grief disorder in adult bereavement: A systematic review and meta-analysis. *J Affect Disord.* 2017;212:138–49.

3 Onrust SA, Cuijpers P. Mood and anxiety disorders in widowhood: a systematic review. *Aging Ment Health.* 2006;10(4):327–34.

4 American Psychiatric Association. Diagnostic and statistical manual of mental disorders (5th ed.). Arlington, VA: American Psychiatric Publishing; 2013.

5 Boelen PA, van de Schoot R, van den Hout MA, et al. Prolonged Grief Disorder, depression, and posttraumatic stress disorder are distinguishable syndromes. *J Affect Disord*. 2010;125(1–3):374–8.

6 Lenferink LIM, Nickerson A, de Keijser J, et al. Trajectories of grief, depression, and posttraumatic stress in disaster-bereaved people. *Depress Anxiety*. 2018.

7 Malgaroli M, Maccallum F, Bonanno GA. Symptoms of persistent complex bereavement disorder, depression, and PTSD in a conjugally bereaved sample: a network analysis. *Psychol Med.* 2018;48(14):2439–48.

For peer review only

For peer review only - http://bmjopen.bmj.com/site/about/guidelines.xhtml

8 Heeke C, Kampisiou C, Niemeyer H, et al. A systematic review and meta-analysis of correlates of prolonged grief disorder in adults exposed to violent loss. *Eur J Psychotraumatology*. 2019;10(1).

9 Kristensen P, Weisæth L, Heir T. Bereavement and mental health after sudden and violent losses: a review. *Psychiatry.* 2012;75(1):76–97.

10 Smid GE, Kleber RJ, Rie SM de la, et al. Brief Eclectic Psychotherapy for Traumatic Grief (BEP-TG): toward integrated treatment of symptoms related to traumatic loss. *Eur J Psychotraumatology.* 2015;6(1):27324.

11 Eisma, M.C., de Keijser, J. Nabestaanden van verkeersongevallen. In: de Keijser, J., Boelen, P.A., Smid, G.E., redacteuren. Handboek traumatische rouw. Amsterdam: Uitgeverij Boom; 2018. p. 104–16.

12 Boelen PA, de Keijser J, Smid G. Cognitive–behavioral variables mediate the impact of violent loss on post-loss psychopathology. *Psychol Trauma Theory Res Pract Policy*. 2015;7(4):382–90.

13 Boelen PA, van den Hout MA, van den Bout J. A Cognitive-Behavioral Conceptualization of Complicated Grief. *Clin Psychol Sci Pract.* 2006;13(2):109–28.

14 Janoff-Bulman R. Assumptive worlds and the stress of traumatic events: Applications of the schema construct. *Soc Cogn.* 1989;7(2):113–36.

15 Boelen PA, Lensvelt-Mulders GJLM. Psychometric Properties of the Grief Cognitions Questionnaire (GCQ). *J Psychopathol Behav Assess.* 2005;27(4):291–303.

For peer review only

16 Foa EB, Riggs DS, Massie ED, et al. The impact of fear activation and anger on the efficacy of exposure treatment for posttraumatic stress disorder. *Behav Ther.* 1995;26(3):487–99.

17 Tehrani N. Road victim trauma: an investigation of the impact on the injured and bereaved. *Couns Psychol Q.* 2004;17(4):361–73.

18 Boelen PA. A Sense of 'unrealness' about the death of a loved-one: An exploratory study of its role in emotional complications among bereaved individuals. *Appl Cogn Psychol.* 2010;24(2):238–51.

19 Boelen PA. "It feels as if she might return one day": A sense of unrealness as a predictor of bereavement-related emotional distress / "Tengo la sensación de que ella puede volver algún día": la sensación de irrealidad como un predictor del sufrimiento emocional relacionado con la pérdida. *Estud Psicol.* 2017;38(3):734–51.

20 Benight CC. Social cognitive theory of posttraumatic recovery: the role of perceived self-efficacy. *Behav Res Ther.* 2004;42(10):1129–48.

21 Boelen PA, Smid GE. Disturbed grief: prolonged grief disorder and persistent complex bereavement disorder. *BMJ.* 2017;357:j2016.

22 Currier JM, Holland JM, Neimeyer RA. Do CBT-Based Interventions Alleviate Distress Following Bereavement? A Review of the Current Evidence. *Int Cog Ther.* 2010;3:77.93.

23 Doering BK, Eisma MC. Treatment for complicated grief: state of the science and ways forward. *Curr Opin Psychiatry.* 2016;29(5):286–91.

For peer review only - http://bmjopen.bmj.com/site/about/guidelines.xhtml

24 Johannsen M, Damholdt MF, Zachariae R, et al. Psychological interventions for grief in adults: A systematic review and meta-analysis of randomized controlled trials. *J Affect Disord*. 2019;253:69–86.

25 Boelen PA, Keijser J de, Hout MA van den, et al. Factors associated with outcome of cognitive–behavioural therapy for complicated grief: A preliminary study. *Clin Psychol Psychother*. 2011;18(4):284–91.

26 Bryant RA, Kenny L, Joscelyne A, et al. Predictors of treatment response for cognitive behaviour therapy for prolonged grief disorder. *Eur J Psychotraumatology.* 2019;8(6).

27 Linde K, Treml J, Steinig J, et al. Grief interventions for people bereaved by suicide: A systematic review. *PloS One*. 2017;12(6):e0179496.

28 Wittouck C, Van Autreve S, De Jaegere E, et al. The prevention and treatment of complicated grief: a meta-analysis. *Clin Psychol Rev*. 2011;31(1):69–78.

29 de Heus A, Hengst SMC, de la Rie SM, et al. Day patient treatment for traumatic grief: preliminary evaluation of a one-year treatment programme for patients with multiple and traumatic losses. *Eur J Psychotraumatology.* 2017;8(1).

30 van Denderen M, de Keijser J, Stewart R, et al. Treating complicated grief and posttraumatic stress in homicidally bereaved individuals: A randomized controlled trial. *Clin Psychol Psychother.* 2018.

31 Eisma MC, Boelen PA, van den Bout J, et al. Internet-Based Exposure and Behavioral Activation for Complicated Grief and Rumination: A Randomized Controlled Trial. *Behav Ther*. 2015;46(6):729–48.

For peer review only - http://bmjopen.bmj.com/site/about/guidelines.xhtml

32 Kersting A, Dölemeyer R, Steinig J, et al. Brief Internet-based intervention reduces posttraumatic stress and prolonged grief in parents after the loss of a child during pregnancy: a randomized controlled trial. *Psychother Psychosom.* 2013;82(6):372–81.

33 Wagner B, Knaevelsrud C, Maercker A. Internet-based cognitive-behavioral therapy for complicated grief: a randomized controlled trial. *Death Stud.* 2006;30(5):429–53.

34 Baumeister H, Reichler L, Munzinger M, et al. The impact of guidance on Internet-based mental health interventions — A systematic review. *Internet Interv.* 2014;1(4):205–15.

35 Lichtenthal WG, Corner GW, Sweeney CR, et al. Mental Health Services for Parents Who Lost a Child to Cancer: If We Build Them, Will They Come? *J Clin Oncol Off J Am Soc Clin Oncol*. 2015;33(20):2246–53.

36 Andersson G, Titov N. Advantages and limitations of Internet-based interventions for common mental disorders. *World Psychiatry*. 2014;13(1):4–11.

37 Melville KM, Casey LM, Kavanagh DJ. Dropout from Internet-based treatment for psychological disorders. *Br J Clin Psychol.* 2010;49(Pt 4):455–71.

38 Pihlaja S, Stenberg J-H, Joutsenniemi K, et al. Therapeutic alliance in guided internet therapy programs for depression and anxiety disorders - A systematic review. *Internet Interv.* 2018;11:1–10.

39 Bordin ES. Theory and research on the therapeutic working alliance: New directions. In: The working alliance: Theory, research, and practice. Oxford, England: John Wiley & Sons; 1994. p. 13–37.

For peer review only

For peer review only - http://bmjopen.bmj.com/site/about/guidelines.xhtml

40 Cook JE, Doyle C. Working alliance in online therapy as compared to face-to-face therapy: preliminary results. *Cyberpsychology Behav Impact Internet Multimed Virtual Real Behav Soc.* 2002;5(2):95–105.

41 Sucala M, Schnur JB, Constantino MJ, et al. The therapeutic relationship in e-therapy for mental health: a systematic review. *J Med Internet Res*. 2012;14(4):e110.

42 Lenferink LIM, de Keijser J, Wessel I, et al. Cognitive behavioural therapy and mindfulness for relatives of missing persons: a pilot study. *Pilot Feasibility Stud.* 2019;5(1):93.

43 Boelen PA, Bout J van den. Protocollaire behandeling van persisterende complexe rouwstoornis. In: Keijsers, G., Minnen, A. van, Verbraak, M., Hoogduin, K., Emmelkamp, P., (Red.). Protocollaire behandelingen voor volwassenen met psychische klachten. Ed. 1. Amsterdam: Boom; 2017.

44 Boelen PA, Smid GE. The Traumatic Grief Inventory Self-Report Version (TGI-SR): Introduction and Preliminary Psychometric Evaluation. *J Loss Trauma.* 2017;22(3):196–212.

45 World Health Organization. ICD-11. Prolonged Grief Disorder Criteria [Internet]. 2018 [Accessed on October 14 2019]. Available from: https://icd.who.int/browse11/l-m/en#/http://id.who.int/icd/entity/1183832314

46 Boelen PA, Djelantik AAAMJ, de Keijser J, et al. Further validation of the Traumatic Grief Inventory-Self Report (TGI-SR): A measure of persistent complex bereavement disorder and prolonged grief disorder. *Death Stud. 2019*;43(6):351–64.

For peer review only - http://bmjopen.bmj.com/site/about/guidelines.xhtml

47 Blevins CA, Weathers FW, Davis MT, et al. The Posttraumatic Stress Disorder Checklist for DSM-5 (PCL-5): Development and Initial Psychometric Evaluation. *J Trauma Stress.* 2015;28(6):489–98.

48 Boeschoten, M.A., Bakker, A., Jongedijk, et al. PTSS checklist voor de DSM-5 (PCL-5). Diemen: Arq Nationaal Psychotrauma Centrum; 2014.

49 Zigmond AS, Snaith RP. The hospital anxiety and depression scale. *Acta Psychiatr Scand.* 1983;67(6):361–70.

50 Spinhoven PH, Ormel J, Sloekers PPA, et al. A validation study of the Hospital Anxiety and Depression scale (HADS) in different groups of Dutch subjects. *Psychol Med.* 1997;27(2):363–70.

51 Mundt JC, Marks IM, Shear MK, et al. The Work and Social Adjustment Scale: a simple measure of impairment in functioning. *Br J Psychiatry J Ment Sci.* 2002;180:461–4.

52 de Graaf LE, Gerhards SH, Arntz A, et al. Clinical effectiveness of online computerised cognitive-behavioural therapy without support for depression in primary care: randomised trial. *Br J Psychiatry J Ment Sci.* 2009;195(1):73–80.

53 Boelen PA, van den Bout J. Anxious and depressive avoidance and symptoms of prolonged grief, depression, and post-traumatic stress disorder. *Psychol Belg.* 2010;50(1–2):49–67.

54 Spielberger, C.D. State-Trait Anger Expression Inventory-2. Psychological Assessment Resources; 1999.

For peer review only - http://bmjopen.bmj.com/site/about/guidelines.xhtml

55 Lievaart M, Franken IHA, Hovens JE. Anger Assessment in Clinical and Nonclinical Populations: Further Validation of the State-Trait Anger Expression Inventory-2. *J Clin Psychol.* 2016;72(3):263–78.

56 Schwarzer, R., Jerusalem, M. Generalized self-efficacy scale. In: Weinman, J., Wright, S., Johnston, M., redacteuren. Measures in health psychology: A user's portfolio Causal and control beliefs. Windsor, UK: Nfer-Nelson; 1995. p. 35–7.

57 Horvath AO, Greenberg LS. Development and validation of the Working Alliance Inventory. *J Couns Psychol.* 1989;36(2):223–33.

58 Vertommen, H., Vervaeke, G. Nederlandstalige experimentele vertaling van de Working Alliance Inventory van Horvath en Greenberg. Leuven: KU; 1990.

59 Munder T, Wilmers F, Leonhart R, et al. Working Alliance Inventory-Short Revised (WAI-SR): psychometric properties in outpatients and inpatients. *Clin Psychol Psychother*. 2010;17(3):231–9.

60 Snijders, T.A.B., Bosker, R.J. Deviance tests. In: Snijders, T.A.B., Bosker, R.J., (Red.). Multilevel analysis. London: SAGE Publications Ltd; 2012. p. 97–8.

61 Little, T. Mediation and moderation. In: Little, T., (Red.). Longitudinal Structural Equation Modeling. New York: The Guilford Press; 2013. p. 286–324.

62 Schumi J, Wittes JT. Through the looking glass: understanding non-inferiority. *Trials.* 2011;12:106.

63 Glickman K, Shear KM, Wall MM. Therapeutic Alliance and Outcome in Complicated Grief Treatment. *Int J Cogn Ther.* 2018;11(2):222–33.

For peer review only - http://bmjopen.bmj.com/site/about/guidelines.xhtml

64 Arjadi R, Nauta MH, Bockting CLH. Acceptability of internet-based interventions for depression in Indonesia. *Internet Interv.* 2018;13:8–15.

65 Lim GY, Tam WW, Lu Y, et al. Prevalence of Depression in the Community from 30 Countries between 1994 and 2014. *Sci Rep.* 2018;8(1):2861.

66 Lenferink, L.I.M., Boelen, P.A., Smid, G.E., et al. The importance of harmonising diagnostic criteria sets for pathological grief. *British Journal of Psychiatry.* in press;

67 Maciejewski PK, Prigerson HG. Prolonged, but not complicated, grief is a mental disorder. *Br J Psychiatry.* 2017;211(4):189–91.

68 Wakefield JC. Should prolonged grief be reclassified as a mental disorder in DSM-5?: reconsidering the empirical and conceptual arguments for complicated grief disorder. *J Nerv Ment Dis.* 2012;200(6):499–511.

Authors' contributions

JdK, LL, and PB are the principal investigators. LL is the executive researcher. JdK, PB, ME, and GS are the grant holders. LL developed the study design and wrote the ethics proposal and drafts of the manuscript. JdK, ME, GS, and PB read, revised, and approved the drafts of the study design, ethics proposal, and the manuscript.

Funding statement

Fund Victim Support subsidized this work. This funder does not have ultimate authority over any of the research activities.

Competing Interests Statement

For peer review only - http://bmjopen.bmj.com/site/about/guidelines.xhtml

All authors declare to have no competing interests.

Patient and Public Involvement

A committee of representatives of bereaved people, non-governmental support organizations, and professionals working with bereaved people is initiated for this project. These committee members are involved in recruitment of participants, pilot-testing research materials, and dissemination of findings.

For peer review only

For peer review only - http://bmjopen.bmj.com/site/about/guidelines.xhtml

Reporting checklist for protocol of a clinical trial.

Based on the SPIRIT guidelines.

Instructions to authors

Complete this checklist by entering the page numbers from your manuscript where readers will find each of the items listed below.

Your article may not currently address all the items on the checklist. Please modify your text to include the missing information. If you are certain that an item does not apply, please write "n/a" and provide a short explanation.

Upload your completed checklist as an extra file when you submit to a journal.

In your methods section, say that you used the SPIRITreporting guidelines, and cite them as:

Chan A-W, Tetzlaff JM, Altman DG, Laupacis A, Gøtzsche PC, Krleža-Jerić K, Hróbjartsson A, Mann H, Dickersin K, Berlin J, Doré C, Parulekar W, Summerskill W, Groves T, Schulz K, Sox H, Rockhold FW, Rennie D, Moher D. SPIRIT 2013 Statement: Defining standard protocol items for clinical trials. Ann Intern Med. 2013;158(3):200-207

		Reporting Item	Page Number
**Administrative information**			
Title	#1	Descriptive title identifying the study design, population, interventions, and, if applicable, trial acronym	1
Trial registration	#2a	Trial identifier and registry name. If not yet registered, name of intended registry	3
Trial registration: data set	#2b	All items from the World Health Organization Trial Registration Data Set	n/a
Protocol version	#3	Date and version identifier	n/a
Funding	#4	Sources and types of financial, material, and other support	32

Roles and responsibilities: contributorship	#5a	Names, affiliations, and roles of protocol contributors	32
Roles and responsibilities: sponsor contact information	#5b	Name and contact information for the trial sponsor	n/a
Roles and responsibilities: sponsor and funder	#5c	Role of study sponsor and funders, if any, in study design; collection, management, analysis, and interpretation of data; writing of the report; and the decision to submit the report for publication, including whether they will have ultimate authority over any of these activities	32
Roles and responsibilities: committees	#5d	Composition, roles, and responsibilities of the coordinating centre, steering committee, endpoint adjudication committee, data management team, and other individuals or groups overseeing the trial, if applicable (see Item 21a for data monitoring committee)	n/a
**Introduction**			
Background and rationale	#6a	Description of research question and justification for undertaking the trial, including summary of relevant studies (published and unpublished) examining benefits and harms for each intervention	4-7
Background and rationale: choice of comparators	#6b	Explanation for choice of comparators	4-7
Objectives	#7	Specific objectives or hypotheses	8
Trial design	#8	Description of trial design including type of trial (eg, parallel group, crossover, factorial, single group), allocation ratio, and framework (eg, superiority, equivalence, non-inferiority, exploratory)	10
**Methods: Participants,**			

For peer review only

**interventions, and outcomes**			
Study setting	#9	Description of study settings (eg, community clinic, academic hospital) and list of countries where data will be collected. Reference to where list of study sites can be obtained	10
Eligibility criteria	#10	Inclusion and exclusion criteria for participants. If applicable, eligibility criteria for study centres and individuals who will perform the interventions (eg, surgeons, psychotherapists)	12
Interventions: description	#11a	Interventions for each group with sufficient detail to allow replication, including how and when they will be administered	13-14
Interventions: modifications	#11b	Criteria for discontinuing or modifying allocated interventions for a given trial participant (eg, drug dose change in response to harms, participant request, or improving / worsening disease)	19
Interventions: adherance	#11c	Strategies to improve adherence to intervention protocols, and any procedures for monitoring adherence (eg, drug tablet return; laboratory tests)	15
Interventions: concomitant care	#11d	Relevant concomitant care and interventions that are permitted or prohibited during the trial	18
Outcomes	#12	Primary, secondary, and other outcomes, including the specific measurement variable (eg, systolic blood pressure), analysis metric (eg, change from baseline, final value, time to event), method of aggregation (eg, median, proportion), and time point for each outcome. Explanation of the clinical relevance of chosen efficacy and harm outcomes is strongly recommended	15-17
Participant timeline	#13	Time schedule of enrolment, interventions (including any run-ins and washouts), assessments, and visits for participants. A schematic diagram is highly recommended (see Figure)	11
Sample size	#14	Estimated number of participants needed to achieve study objectives and how it was determined, including	12

For peer review only

		clinical and statistical assumptions supporting any sample size calculations	
Recruitment	#15	Strategies for achieving adequate participant enrolment to reach target sample size	11-12
**Methods: Assignment of interventions (for controlled trials)**			
Allocation: sequence generation	#16a	Method of generating the allocation sequence (eg, computer-generated random numbers), and list of any factors for stratification. To reduce predictability of a random sequence, details of any planned restriction (eg, blocking) should be provided in a separate document that is unavailable to those who enrol participants or assign interventions	10
Allocation concealment mechanism	#16b	Mechanism of implementing the allocation sequence (eg, central telephone; sequentially numbered, opaque, sealed envelopes), describing any steps to conceal the sequence until interventions are assigned	10
Allocation: implementation	#16c	Who will generate the allocation sequence, who will enrol participants, and who will assign participants to interventions	10
Blinding (masking)	#17a	Who will be blinded after assignment to interventions (eg, trial participants, care providers, outcome assessors, data analysts), and how	10
Blinding (masking): emergency unblinding	#17b	If blinded, circumstances under which unblinding is permissible, and procedure for revealing a participant's allocated intervention during the trial	n/a
**Methods: Data collection, management, and analysis**			
Data collection plan	#18a	Plans for assessment and collection of outcome, baseline, and other trial data, including any related processes to promote data quality (eg, duplicate	15-18

For peer review only

		measurements, training of assessors) and a description of study instruments (eg, questionnaires, laboratory tests) along with their reliability and validity, if known. Reference to where data collection forms can be found, if not in the protocol	
Data collection plan: retention	#18b	Plans to promote participant retention and complete follow-up, including list of any outcome data to be collected for participants who discontinue or deviate from intervention protocols	n/a
Data management	#19	Plans for data entry, coding, security, and storage, including any related processes to promote data quality (eg, double data entry; range checks for data values). Reference to where details of data management procedures can be found, if not in the protocol	20
Statistics: outcomes	#20a	Statistical methods for analysing primary and secondary outcomes. Reference to where other details of the statistical analysis plan can be found, if not in the protocol	18-19
Statistics: additional analyses	#20b	Methods for any additional analyses (eg, subgroup and adjusted analyses)	18-19
Statistics: analysis population and missing data	#20c	Definition of analysis population relating to protocol non-adherence (eg, as randomised analysis), and any statistical methods to handle missing data (eg, multiple imputation)	18-19
**Methods: Monitoring**			
Data monitoring: formal committee	#21a	Composition of data monitoring committee (DMC); summary of its role and reporting structure; statement of whether it is independent from the sponsor and competing interests; and reference to where further details about its charter can be found, if not in the protocol. Alternatively, an explanation of why a DMC is not needed	20
Data monitoring: interim analysis	#21b	Description of any interim analyses and stopping guidelines, including who will have access to these	20

For peer review only

		interim results and make the final decision to terminate the trial	
Harms	#22	Plans for collecting, assessing, reporting, and managing solicited and spontaneously reported adverse events and other unintended effects of trial interventions or trial conduct	20
Auditing	#23	Frequency and procedures for auditing trial conduct, if any, and whether the process will be independent from investigators and the sponsor	20
**Ethics and dissemination**			
Research ethics approval	#24	Plans for seeking research ethics committee / institutional review board (REC / IRB) approval	20
Protocol amendments	#25	Plans for communicating important protocol modifications (eg, changes to eligibility criteria, outcomes, analyses) to relevant parties (eg, investigators, REC / IRBs, trial participants, trial registries, journals, regulators)	20
Consent or assent	#26a	Who will obtain informed consent or assent from potential trial participants or authorised surrogates, and how (see Item 32)	20
Consent or assent: ancillary studies	#26b	Additional consent provisions for collection and use of participant data and biological specimens in ancillary studies, if applicable	n/a
Confidentiality	#27	How personal information about potential and enrolled participants will be collected, shared, and maintained in order to protect confidentiality before, during, and after the trial	20
Declaration of interests	#28	Financial and other competing interests for principal investigators for the overall trial and each study site	32
Data access	#29	Statement of who will have access to the final trial dataset, and disclosure of contractual agreements that limit such access for investigators	20

For peer review only

Ancillary and post trial care	#30	Provisions, if any, for ancillary and post-trial care, and for compensation to those who suffer harm from trial participation	20
Dissemination policy: trial results	#31a	Plans for investigators and sponsor to communicate trial results to participants, healthcare professionals, the public, and other relevant groups (eg, via publication, reporting in results databases, or other data sharing arrangements), including any publication restrictions	19-20
Dissemination policy: authorship	#31b	Authorship eligibility guidelines and any intended use of professional writers	n/a
Dissemination policy: reproducible research	#31c	Plans, if any, for granting public access to the full protocol, participant-level dataset, and statistical code	n/a
**Appendices**			
Informed consent materials	#32	Model consent form and other related documentation given to participants and authorised surrogates	n/a
Biological specimens	#33	Plans for collection, laboratory evaluation, and storage of biological specimens for genetic or molecular analysis in the current trial and for future use in ancillary studies, if applicable	n/a

None The SPIRIT checklist is distributed under the terms of the Creative Commons Attribution License CC-BY-ND 3.0. This checklist can be completed online using https://www.goodreports.org/, a tool made by the EQUATOR Network in collaboration with Penelope.ai

For peer review only

Online cognitive behavioral therapy for traumatically bereaved people: Study protocol for a randomized waitlist-controlled trial

Journal:	*BMJ Open*
Manuscript ID	bmjopen-2019-035050.R1
Article Type:	Protocol
Date Submitted by the Author:	11-Jun-2020
Complete List of Authors:	Lenferink, Lonneke; Rijksuniversiteit Groningen, Clinical Psychology and Experimental Psychopathology; Utrecht University, Clinical Psychology<br>de Keijser, Jos; Rijksuniversiteit Groningen, Clinical Psychology and Experimental Psychopathology<br>Eisma, Maarten; Clinical Psychology and Experimental Psychopathology<br>Boelen, Paul; Utrecht University, Clinical Psychology; Foundation Centrum '45<br>Smid, Geert; Foundation Centrum '45
**Primary Subject Heading**:	Mental health
Secondary Subject Heading:	Mental health, Evidence based practice
Keywords:	PSYCHIATRY, Adult psychiatry < PSYCHIATRY, PUBLIC HEALTH

SCHOLARONE™
Manuscripts

I, the Submitting Author has the right to grant and does grant on behalf of all authors of the Work (as defined in the below author licence), an exclusive licence and/or a non-exclusive licence for contributions from authors who are: i) UK Crown employees; ii) where BMJ has agreed a CC-BY licence shall apply, and/or iii) in accordance with the terms applicable for US Federal Government officers or employees acting as part of their official duties; on a worldwide, perpetual, irrevocable, royalty-free basis to BMJ Publishing Group Ltd ("BMJ") its licensees and where the relevant Journal is co-owned by BMJ to the co-owners of the Journal, to publish the Work in this journal and any other BMJ products and to exploit all rights, as set out in our licence.

The Submitting Author accepts and understands that any supply made under these terms is made by BMJ to the Submitting Author unless you are acting as an employee on behalf of your employer or a postgraduate student of an affiliated institution which is paying any applicable article publishing charge ("APC") for Open Access articles. Where the Submitting Author wishes to make the Work available on an Open Access basis (and intends to pay the relevant APC), the terms of reuse of such Open Access shall be governed by a Creative Commons licence – details of these licences and which Creative Commons licence will apply to this Work are set out in our licence referred to above.

Other than as permitted in any relevant BMJ Author's Self Archiving Policies, I confirm this Work has not been accepted for publication elsewhere, is not being considered for publication elsewhere and does not duplicate material already published. I confirm all authors consent to publication of this Work and authorise the granting of this licence.

For peer review only

Online cognitive behavioral therapy for traumatically bereaved people: Study protocol for a randomized waitlist-controlled trial

Lenferink, L.I.M.*[1,2], de Keijser, J.[1], Eisma, M.C.[1], Smid, G.E.[3,4,5], & Boelen, P.A.[2,3,4]

[1] Department of Clinical Psychology and Experimental Psychopathology, Faculty of Behavioral and Social Sciences, University of Groningen, Grote Kruisstraat 2/1, 9712 TS Groningen, The Netherlands. E-mail L.I.M. Lenferink: l.i.m.lenferink@rug.nl.

[2] Department of Clinical Psychology, Faculty of Social Sciences, Utrecht University, P.O. Box 80140, 3508 TC, Utrecht, The Netherlands.

[3] ARQ Nationaal Psychotrauma Centre, Nienoord 5, 1112 XE, Diemen, The Netherlands

[4] Foundation Centrum '45, Nienoord 5, 1112 XE Diemen, The Netherlands

[5] University of Humanistic Studies, Utrecht, The Netherlands

*Corresponding author

For peer review only

Abstract

Introduction

The traumatic death of a loved one, such as death due to a traffic accident, can precipitate persistent complex bereavement disorder (PCBD) and comorbid posttraumatic stress disorder (PTSD) and depression. Waitlist-controlled trials have shown that grief-specific cognitive behavioral therapy (CBT) is an effective treatment for such mental health problems. This is the first study that will examine the effectiveness of online CBT (vs. waitlist controls) in a sample exclusively comprised of people bereaved by a traumatic death. Our primary hypothesis is that people allocated to the online CBT condition will show larger reductions in PCBD, PTSD, and depression symptom levels at post-treatment than people allocated to a waitlist. We further expect that reductions in symptom levels during treatment are associated with reductions of negative cognitions and avoidance behaviors and the experience of fewer accident-related stressors. Moreover, the effect of the quality of the therapeutic alliance on treatment effects and drop-out rates will be explored.

Methods and analysis

A two-arm (online CBT vs. waiting list) open label parallel randomized controlled trial (RCT) will be conducted. Participants will complete questionnaires at pre-treatment and 12 and 20 weeks after study enrolment. Eligible for participation are Dutch adults who lost a loved one at least one year earlier due to a traffic accident and report clinically relevant levels of PCBD, PTSD, and/or depression. Multilevel modeling will be used.

Ethics and dissemination

For peer review only - http://bmjopen.bmj.com/site/about/guidelines.xhtml

Ethics approval has been received (METc UMCG: M20.252121). This study will provide new insights in the effectiveness of online CBT for traumatically bereaved people. If the treatment is demonstrated to be effective, it will be made publicly accessible. Findings will be disseminated among lay people (e.g., through newsletters and media performances), our collaborators (e.g., through presentations at support organizations), and clinicians and researchers (e.g., through conference presentations and scientific journal articles).

Strengths and limitations of this study

- This study is the first to examine the effectiveness of online CBT (vs. waitlist controls) in reducing psychopathology after traumatic loss in an RCT.
- This study is one of the first to examine potential correlates of change in symptom levels following online treatment after traumatic loss.
- We are not able to formally test mediators or moderators of treatment effects.
- We are not able to examine if online CBT has equal effects as face-to-face CBT.
- We are not able to establish formal diagnoses, as we use self-report questionnaires, instead of diagnostic interviews, to assess symptom levels.

Trial registration number: NL7497 (Dutch Trial Register)

Word Count: 5,133 words including main text

For peer review only - http://bmjopen.bmj.com/site/about/guidelines.xhtml

Worldwide, traffic accidents represent the leading cause of unnatural deaths(1). Ten to 20 percent of bereaved people who experience natural deaths (e.g., illness) develop severe and persistent grief-related distress, including persistent complex bereavement disorder (PCBD), posttraumatic stress disorder (PTSD), and depression(2,3). Notably, PCBD has been introduced as other specified trauma- and stressor-related disorder, in the latest version of the Diagnostic and Statistical manual of Mental Diseases (DSM-5)(4). PCBD can be diagnosed if, after the death of a significant other at least 12 months earlier, a person experiences persistent yearning for the deceased and symptoms of reactive distress (e.g., emotional numbness) and social/identity disruption (e.g., feeling alone) causing impairment in daily life. While some PCBD symptoms overlap with PTSD (e.g., anger) and depression symptoms (e.g., diminished interest in activities), several studies have shown that these three syndromes are distinct(5–7). Unexpected/violent losses of a significant other, also referred to as a traumatic losses, including deaths caused by traffic accidents, increase risks for the development of PCBD, PTSD, and depression(8,9).

Heightened risk for developing psychopathology after deaths due to traffic accident

Specific circumstances of losses caused by accidents may account for the elevated risk of grief-related distress. For instance, experiencing multiple losses simultaneously, being a witness to the accident, and juridical and financial consequences are proposed to exacerbate grief-related distress(10). Furthermore, negative cognitions and avoidance behaviors may mediate the influence of sudden/violent loss on grief, PTSD, and depression levels(11). According to a cognitive-behavioral model three interacting malleable processes underlie disturbed grief reactions: i) negative cognitions, ii) avoidance behavior, and iii) difficulties integrating the loss into the autobiographical knowledge base(12).

For peer review only - http://bmjopen.bmj.com/site/about/guidelines.xhtml

Experiencing a loss due to a traffic accident may violate basic assumptions about the world being a safe place(13). This may fuel negative cognitions (e.g., “I’m less worthy, since s/he died” and “The death of him/her has taught me that the world is unjust) that may exacerbate and maintain acute grief responses(14). Avoidance behaviors include depressive avoidance and anxious avoidance strategies. Depressive avoidance refers to withdrawal from social and occupational activities that were perceived as fulfilling before the death, out of the conviction that these activities are no longer meaningful. Anxious avoidance strategies serve to prevent confrontation with the reality of the death, out of fear that confrontation is too painful(12). One potential way to avoid confrontation with the reality of the loss, is to focus on angry thoughts and feelings (e.g., “I was angry at the police, courts, or administration, because they did not do their work well enough“)(15). This seems to be a frequently used avoidant coping strategy in bereaved people after traffic accidents and is strongly related to PTSD(16). Difficulty with integration of the loss into the autobiographical knowledge base refers to the difficulties connecting factual knowledge that the loss is irreversible with existing information about the self and the relationship with the lost person, stored in autobiographical memory. Memories related to the loss may lack context in terms of time and place, causing the loss to be experienced as unreal(17). It has been argued that this “sense of unrealness” may trigger intrusive memories and increase feelings of numbness or shock once the bereaved person is confronted with reminders of the loss(17,18). The extent to which a person believes that one is capable of managing stressor-related thoughts, emotions, and behaviors, also referred to as self-efficacy (e.g., “I can usually handle whatever comes my way“), has also been determined as an important factor facilitating coping with traumatic stressors(19). Decreased self-efficacy, negative cognitions

For peer review only - http://bmjopen.bmj.com/site/about/guidelines.xhtml

and insufficient integration of the loss may contribute to increased sensitivity to loss reminders or secondary stressors following traumatic loss(20).

Cognitive-behavioral therapy for grief-related distress

Grief-specific CBT has been demonstrated to be the most effective treatment for bereaved people with elevated grief levels(21–24). CBT targets the abovementioned cognitive-behavioral variables with cognitive restructuring, loss-related exposure, and behavioral activation. Notably, research on putative mechanisms of change of grief-specific CBT is sparse(23) (but see(25,26)). Examining the effectiveness of grief-specific CBT and its potential mechanisms of change in traumatically bereaved people with traumatic grief is clinically relevant because it would enable tailoring of interventions to the specific needs of this group, which could improve treatment outcomes(27).

Whilst the majority of trials assess the efficacy of face-to-face CBT(24), so far, to the best of our knowledge, three online CBT-based interventions have been developed for distressed bereaved people(28–30). These prior studies provided preliminary data on the potential effectiveness of online grief-specific CBT, but had some limitations. For instance, treatment was solely provided to people who experienced perinatal loss (29) or included relatively small samples (28). Comparability between these three studies is also limited, because interventions differed in treatment content; different elements of CBT were offered, for instance behavioral activation, exposure (28), or writing assignments (29,30). Offering CBT via the internet has some potential advantages. It may lower the threshold for seeking treatment, because it can be delivered independent of geographical location. Furthermore, asynchronous communication may be used, allowing the client and therapist can contact each other at any preferred time(31). This may counter barriers to mental health

For peer review only - http://bmjopen.bmj.com/site/about/guidelines.xhtml

service use, such as difficulties with finding help, transportation concerns, or difficulties scheduling treatment sessions(32). In addition, online CBT could reduce treatment costs, improving accessibility and dissemination of care for people in need of support(33). Moreover, during times of a crisis, such as the COVID-19 pandemic, it seems more relevant than ever to further examine the effectiveness of online CBT for distressed bereaved people, as it will allow them to retain access to evidence-based care (34).

A potential downside to online CBT is the high dropout rate found in earlier studies(33,35). It has been argued that a strong therapeutic alliance might support adherence to online treatment and mediates treatment effects (36). Therapeutic alliance is defined as a positive emotional bond between client and therapist, whereby both parties agree on the tasks and goals of the treatment(37). The client-therapist relationship might also explain why online treatments are more effective with therapist guidance than without (31). Concerns have been raised that developing a therapeutic relationship might be more difficult when non-verbal communication is absent(38). However, studies in non-bereaved samples indicate that developing a strong therapeutic alliance is possible during online treatment(33) and that therapeutic alliance is often related to online treatment outcomes(39), but not always (33). More research is needed to further examine the interrelations of the quality of client-therapist relationship, drop-out, and treatment outcomes in online CBT.

Study objectives

Our first aim is to examine the effectiveness of online CBT (vs. a waiting list control condition) in reducing symptom levels of PCBD, PTSD, and depression in people bereaved by a traffic accident. We expect that participants assigned to the online CBT condition will show

For peer review only

larger reductions in symptom levels of PCBD, PTSD, and depression compared with waitlist controls at post-treatment assessments (Hypothesis 1).

Our second aim is to explore correlates of change. Based on prior research and theories(12,16,19), we expect that reductions in negative cognitions, avoidance behaviors, state anger, a sense of unrealness, and improvement in self-efficacy are related to reductions in PCBD, PTSD, and depression levels in online CBT (Hypothesis 2a). Additionally, we aim to explore whether background characteristics (i.e., gender, age, and educational level, kinship to the deceased, and time since loss) and accident-related stressors (i.e., single vs. multiple loss, witnessing the accident, and status of legal trial) are related to treatment effects (Hypothesis 2b). We have no specific expectations regarding these associations because prior treatment studies in bereaved people showed inconsistent results(24,25,40). However, based on clinical experience, we expect that accident-related stressors are associated with treatment effects, such that multiple loss, witnessing the accident, and on-going legal trial negatively impact treatment effects.

Our third aim is to explore the associations between quality of the therapeutic alliance and drop-out rates and treatment outcomes. We expect that a stronger therapeutic alliance is related to lower dropout rates and better treatment outcomes.

Methods and analysis

Design

A two-arm (online CBT vs. waiting list) multi-centre open label parallel RCT will be conducted. Randomization will take place after the participant is screened for eligibility-based inclusion criteria (described below). A random number generator (www.random.org)

For peer review only - http://bmjopen.bmj.com/site/about/guidelines.xhtml

will be used by a blinded independent researcher, to perform the blocking randomization procedure. An allocation ratio of 1:1 will be applied.

Participants allocated to the online CBT condition receive treatment within one week after allocation. All participants will be asked to fill in questionnaires (described below) at baseline (T1), 12 weeks post-allocation (T2 for the intervention condition and T1a for waitlist controls), and 20 weeks post-allocation (T3 for the intervention condition and T1b for waitlist controls). For participants in the waiting list control group, at the end of the 20-week waiting period after which they will receive online CBT, they will be asked to fill in T2 and T3 12 and 20 weeks after starting treatment, respectively (see Figure 1). A link to online questionnaires will be sent to the participants by a non-blinded member of the research team at each time-point. A waitlist control group (instead of a no treatment control group) is chosen to increase the likelihood of continued study participation by guaranteeing that all participants receive treatment. Furthermore, the inclusion of a waiting list control group allows a treatment versus no treatment comparison, that will provide knowledge about the effects of treatment relative to natural recovery from loss.

In line with prior treatment studies from our research group(40,41), the treatment is guided by licensed and registered psychologists who are a member of our Traumatic Loss Network (i.e., an informal national network of trauma and grief therapists that are involved in research projects of our research group). In total six therapists (including authors PB and JdK who are registered clinical psychologists) will guide the participants online; participants will receive feedback from the same therapist each time. The therapists will receive a training, provided by LL, PB, and JdK, on the use of the treatment protocol of this intervention study. In preparation for the training, therapists read all treatment materials

For peer review only - http://bmjopen.bmj.com/site/about/guidelines.xhtml

and a selection of grief treatment literature. Instructions about the use of the online treatment interface will be given by its developers. During a 5-hour face-to-face group meeting the rationale of the online treatment will be explained and research procedures will be discussed. In a 2-hour online video-meeting outstanding questions regarding the treatment and the research project will be answered. Supervision (by telephone or mail) by PB and JdK is possible on request, for instance when therapists encounter difficulties in treatment. Therapists will be contacted by a member of the research team by phone or email biweekly to monitor treatment progress and protocol adherence. Treatment costs will be reimbursed.

==Figure 1 about here==

Participants

This RCT is part of a larger on-going research project (the "TrafVic-project") examining the psychological impact of, and care after, the death of a loved one due to a traffic accident. We expect to recruit the majority of the participants via a survey that started in December 2018 and included the following question: "In this study we would like to offer psychological help to persons who experience emotional problems. May we approach you with more information about this offer, if your answers to this questionnaire show that you experience emotional problems?" Those who answered 'yes' will be sent a letter with information about the intervention, the treatment study, and an informed consent form (see Supplementary Materials). A Dutch website (www.rouwnaverkeersongeval.nl) has been developed so that potential participants can read information about the research and treatment. People who are interested can also sign up for the study via this website. Recruitment for this RCT had not started at the time of submission of this manuscript.

For peer review only - http://bmjopen.bmj.com/site/about/guidelines.xhtml

To be eligible for study participation, the person must 1) be a family member, spouse, or friend of a person who died due to a traffic accident at least one year earlier, 2) be ≥18 years of age, and 3) meet DSM-5 criteria for PCBD and/or PTSD and/or experience clinically relevant depression, based on questionnaire scores (see below for more details). People are excluded when they do not master the Dutch language or have no Internet access.

Sample size

To test our primary hypothesis (Hypothesis 1), a test for each outcome separately (PCBD, PTSD, and depression) will be conducted to assess the effects of online CBT vs. waitlist controls. To find a difference between two groups (online CBT vs. waitlist controls) of at least a medium effect size (f = 0.25; based on prior research(22,28,40)) with a power of 80%, an α of 0.017 (corrected for multiple testing, i.e., 0.05/3, as there are three primary outcome measures (PCBD, PTSD, and depression)), and a strong association (r = .50) between the pre- and post-assessment, a sample size of 23 per condition is sufficient. Taking into account an average dropout rate of 19% (22), a total sample size of 55 (46+9) is required to test Hypothesis 1.

Because our data are nested (repeated measures) (level 1) within individuals (level 2), and possibly within families sharing the same household (level 3), multi-level modeling will be performed to test hypothesis 1. Conducting a power analysis within a multi-level framework is not feasible for various reasons(42). Our power analysis is therefore based on a repeated measures ANOVA.

Intervention

For peer review only - http://bmjopen.bmj.com/site/about/guidelines.xhtml

Online CBT will consist of eight one-on-one sessions, called lessons, offered within a timeframe of 12 weeks. Eight sessions have shown to be sufficient to yield clinically relevant effects in prior research(40). Following Dutch guidelines for grief-specific CBT(42), central components of the treatment are exposure, cognitive restructuring, and behavioral activation. In the first session, psychoeducation is offered, including information about possible emotional reactions to the death of a loved one in a traffic accident and processes that might foster or hamper recovery. A rationale for the CBT interventions is provided.

Then, session 2, 3, and 4 are focused on exposure; the circumstances and story of the loss are presented in detail, and the participant is encouraged to confront stimuli that s/he tends to avoid. Exposure is conducted by imaginary exposure assignments and by writing assignment that have proven to be effective in prior research(30). These writing assignments are focused on writing a detailed narrative of the loss and its circumstances.

The next sessions (5 and 6) focus on identifying and changing negative cognitions that hamper adjustment (i.e., cognitive restructuring); specific attention is paid to cognitions connected with responsibility/guilt and anger that may be experienced following the accidental death(10). Cognitive restructuring assignments are provided to gain an alternative perspective on negative thoughts about the self, life, the future, through 1) psycho-education about common unhelpful thoughts, 2) identifying one's own unhelpful thoughts, and 3) challenging these thoughts. Participants are instructed to undertake these three steps by providing a daily description of i) an emotional moment/event, ii) their thoughts during this event, iii) their feelings (and intensity of these feelings on a scale of 1 through 10), iv) their behavior, v) evaluation of their thoughts), and vi) alternative helpful thoughts.

For peer review only - http://bmjopen.bmj.com/site/about/guidelines.xhtml

In session 7 and 8, participants are encouraged to re-engage in previously valued social, recreational, and occupational activities in order to facilitate the process of adjustment. Behavioral activation assignments are focused on writing about valued activities and making plans to achieve valued goals. Session 8 is also focused on what the participant has learned and how to deal with difficulties in the future.

All information and assignments are presented in an online framework, offered via a secure website. Participants receive online written information that consists of psychoeducation, information about treatment content and structure, and homework assignments. As part of the online treatment, participants also listen to a video-therapist verbally sharing parts of information that are also presented in text. The video-therapists are two therapists from the Traumatic Loss Network; one male and one female and both middle-aged. At the start of the treatment the video-therapists introduce themselves and the participant is asked to select one of the video-therapists. The information shared by these video-therapists are recorded in video-messages in which they read parts of the texts out loud. Each participant therefore receives the same information from a video-therapist. Direct contact with the video-therapist is not possible.

Participants receive weekly asynchronous written feedback from one online therapist on each assignment that they complete online. As mentioned earlier, six online therapists are trained to guide the participants. The online therapists are instructed to contact the participant twice a week; once to encourage participants to log in and complete assignments and once to provide feedback on assignments. In total, they spend 30 minutes per week on reading assignments and providing feedback. Moreover, participants are encouraged to ask

For peer review only - http://bmjopen.bmj.com/site/about/guidelines.xhtml

a family member or friend to support them during treatment. This support figure is then informed about the treatment through written information in an online framework.

Measures

Primary outcome measures

PCBD will be assessed with the Traumatic Grief Inventory-Self Report (TGI-SR)(43). The TGI-SR consists of 18 items on a 5-point Likert scale ranging from 1 = never through 5 = always. Four items tapping disturbed grief criteria according to the 11th edition of the International Classification of Diseases were added(44). An example of an item is: "I found it difficult to trust others". The instruction of the original questionnaire was altered from referring to "the death of your loved one" to "the death of your loved one(s) due to a traffic accident". Psychometric properties of the TGI-SR are adequate(43,45). Participants are considered to meet criteria for DSM-5 PCBD(4) when they score at least 3 ("Sometimes") on at least 1 criterion B symptom (Item 1, item 2, item 3, and item 14), at least 6 criterion C symptoms (item 4 up to 11, and item 15 up to 18), and the criterion D symptom (item 13).

PTSD will be assessed with the PTSD Checklist for DSM-5 (PCL-5)(46) (Dutch version: (47)). Participants rate how often they were bothered by each symptom (e.g., "In the past month, how much were you bothered by trouble remembering important parts of the accident?") on 5-point Likert scales (0 = not at all and 4 = extremely). The instruction and the items of the original questionnaire are altered from referring to the "stressful event" to the "the death of your loved one(s) due to a traffic accident". The PCL-5 has shown to be reliable and valid(46). Participants meet the criteria for DSM-5 PTSD(4) when they score at least 2 ("Moderately") on 1 criterion B item (items 1-5), 1 criterion C item (items 6-7), 2 criterion D items (items 8-14), and 2 criterion E items (items 15-20).

For peer review only - http://bmjopen.bmj.com/site/about/guidelines.xhtml

Depression symptom levels are assessed with the depression subscale of the HADS-D(48). The HADS-D consists of 7 items (e.g., “I still enjoy the thing I used to do”) rated on 4-point scores ranging from 0 (e.g., “Hardly at all”) through 3 (e.g., “Definitely as much”). The Dutch HADS-D is a reliable and valid screening tool for depression(49). A cut-off score of ≥8 is used as indicator for clinically relevant depression(48).

Secondary outcome measures

Negative grief-related cognitions are assessed with 18 items from the Grief Cognitions Questionnaire (GCQ)(14). Participants are asked to rate their agreement with each item (e.g., “Since [–] is dead, I feel less worthy”) on 6-point scales varying from 0 = disagree strongly through 5 = agree strongly. The psychometric properties have been positively evaluated in prior research(14).

Avoidance is measured with the Depressive and Anxious Avoidance in Prolonged Grief Questionnaire (DAAPGQ)(50). The depressive avoidance subscale consists of 5 items (e.g. ‘Since [–] is dead, I do much less of the things that I used to enjoy.’) and the anxious avoidance subscale consists of 4 items (e.g., ‘I avoid to dwell on painful thoughts and memories connected to his/her death.’). Participants answer each item on an 8-point scale with 0 = not at all true for me, and 7 = completely true for me. The DAAPGQ has adequate psychometric properties(50).

State anger is assessed with the 15-item state anger subscale of the State-Trait Anger Expression Inventory-2 (STAXI-2)(51) (Dutch version:(52)). Participants are asked to rate on 4-point Likert scales (1 = not at all and 4 = extremely) how angry they feel right now (e.g., “I feel annoyed”). The STAXI-2 is a valid and reliable measure to assess state anger(52).

For peer review only - http://bmjopen.bmj.com/site/about/guidelines.xhtml

A sense of unrealness is measured with the 5-item Experienced Unrealness Scale(17). Participants are asked to rate their agreement with each item (e.g., "I still can hardly imagine that [–] will never be here again") on 8-point scales (0 = not at all true for me 7 = completely true for me). This instrument demonstrated adequate psychometric properties(17).

Self-efficacy is assessed with the General Self-Efficacy Scale (GSES)(53). The GSES is a 10-item measure. Participants are asked to rate their agreement with each item (e.g., "I can solve most problems if I invest the necessary effort.") on a 4-point scale (1 = completely not true, 4 = completely true). The GSES has shown excellent reliability and validity(53).

Quality of the therapeutic alliance is measured with the 12-item Work Alliance Inventory-Short Form, Client Version and Therapist Version after session 4 (WAI-SF)(54) (Dutch version:(55). The WAI-SF consists of 12 items (e.g., Client version: "We agree on what is important for me to work on", Therapist Version: "We are working towards mutually agreed upon goals.") that are rated on 5-point scales (1 = never and 5 = always). Higher total scores indicate a higher quality of the therapeutic alliance as perceived by the participant and therapist. The WAI-SF is a reliable and valid assessment tool(56).

Other measures

Background characteristics (i.e., gender, age, and educational level, kinship to the deceased, and time since loss) and accident-related stressors (i.e., single vs. multiple loss, witnessed the accident, and status of legal trial) will be assessed with single items.

Participants are allowed to receive other forms of psychosocial, instrumental or legal support during participation in the trial. Using a single question we will assess whether the participants received other forms of psychosocial professional support. The following

For peer review only - http://bmjopen.bmj.com/site/about/guidelines.xhtml

question will be used: “During the past 12 weeks/8 weeks (for T2 and T3, respectively) did you receive additional psychological professional support from a psychologist, therapist or psychiatrist other than the (online) therapist from the TrafVic-study?” We will also include two dichotomous items (yes/no) at T1 to assess psychological support received prior to participation in the study, namely: “Did you ever receive support from a psychologist, therapist or psychiatrist, for your own emotional/mental problems, prior to the loss of your loved one due to a traffic accident?” and “Did you ever receive support from a psychologist, therapist or psychiatrist, for your own emotional/mental problems, related to the loss of your loved one due to a traffic accident?”

Statistical analyses

To examine the differences in reductions of symptom levels of PCBD, PTSD, and depression from pre- to post-treatment/waiting period between the conditions (online CBT vs. waitlist), three independent multi-level models will be built (Hypothesis 1). Symptom levels of PCBD, PTSD, and depression will consecutively be included as dependent variables and condition (online CBT vs. waitlist controls), time, and time x condition (interaction term) as predictor variables, taking into account that repeated observations (level 1) are nested within individuals (level 2), and within households (level 3; if applicable). Additionally, relevant background, loss-related variables, and use of co-interventions (yes/no) during participation in our study, will be included in the analysis as covariates. Deviance tests will be used to examine whether inclusion of these covariates improves model fit(57). Data of all participants entering the study will be included in all analyses (i.e., intention-to-treat analysis). Furthermore, percentages of people meeting diagnostic criteria for PCBD, PTSD,

For peer review only - http://bmjopen.bmj.com/site/about/guidelines.xhtml

and clinically relevant depression will be calculated for each measurement occasion and percentages of people reporting reliable change scores for each outcome measure, using a formula from Jacobson and Truax(58, p. 14), will be reported.

To examine to what extent symptom improvement after treatment is related to improvement in possible correlates of change, residual gain scores will be calculated for all outcome measures (i.e., PCBD, PTSD, and depression) and possible correlates of change (i.e., negative cognitions, avoidance behaviors, state anger, a sense of unrealness, and self-efficacy). Following previous research (cf. (59)), residual gain scores will be calculated by subtracting the standardized combined pre-treatment scores of both conditions (T1 data from immediate treatment condition and T1b data from waitlist condition) multiplied by the correlation coefficient between standardized combined pre-treatment scores and standardized post-treatment (or follow-up) scores from standardized post-treatment (or follow up) scores. To test hypothesis 2a, multiple regression analyses will be conducted to examine the associations between residual gain scores of PCBD, PTSD, or depression and residual gain scores of negative cognitions, avoidance behaviors, state anger, a sense of unrealness, and self-efficacy.

To achieve research aim 2b, multiple regression analyses will be used to examine to what extent residual gain scores of PCBD, PTSD, and depression varies as function of a) background characteristics, including gender (male/female), age (in years), and educational level (low/high), kinship to the deceased (child/spouse vs other), and time since loss (in years) and b) accident-related stressors, including number of losses (single vs multiple), witnessing the accident (yes/no), and status of legal trial (not applicable/on-

For peer review only - http://bmjopen.bmj.com/site/about/guidelines.xhtml

going/completed). Condition (intervention vs. waitlist controls) will be added as a covariate to fulfill research aim 2a and 2b.

To achieve the third research aim, a) differences in therapeutic alliance scores will be assessed between people who completed and dropped out of treatment and b) multiple regression analyses will be used to examine to what extent symptom improvement in PCBD, PTSD, and depression is related to therapeutic alliance (from both participant and therapist perspectives).

Ethics and dissemination

This study has been approved by a local ethics committee (METc UMCG: ID number: M20.252121). The study will be conducted according to the principles of the Declaration of Helsinki (8th version, 2013) and in accordance with the Medical Research Involving Human Subjects Act. Collected data will be handled confidentially, according to the EU General Data Protection Regulation and the Dutch Act on Implementation of the General Data Protection Regulation. Unidentifiable data from this trial will be stored in data repositories from the University of Groningen and Utrecht University.

Findings of this RCT will be disseminated among participants by means of a newsletter. If shown to be effective, the online framework will be made publicly accessible, so that it can benefit other bereaved people. Findings will also be disseminated among lay people by uploading the newsletters on our website (www.rouwnaverkeersongeval.nl) and through media performances. Our findings will be presented to our collaborators, including non-governmental organizations and (peer-)support organizations for bereaved people. Treatment materials will also be made available upon request. Lastly, colleagues will be

For peer review only

informed about our findings during presentations at (inter)national conferences and publications in scientific journals.

Patient and public involvement

No patient involvement.

For peer review only

For peer review only - http://bmjopen.bmj.com/site/about/guidelines.xhtml

Discussion

The relatively few RCTs among general bereaved people with elevated grief levels indicate that grief-specific CBT-based interventions yield the largest effects on post-loss mental health compared with a waiting list(21–24). RCTs evaluating face-to-face or online treatment effects for people with elevated mental health complaints after confrontation with sudden/violent losses are lacking, with the exception of two studies that compared face-to-face EMDR plus CBT against waitlist controls(40,59). Given that traumatically bereaved people are at risk for PCBD and comorbid PTSD and depression(8), it seems particularly relevant to develop evidence-based interventions for this population.

This will be the first RCT to examine the effectiveness of online CBT in a sample exclusively comprised of people who experienced a traumatic death. We are not able to test whether the online CBT has equal effects as face-to-face CBT. Nonetheless, the findings are expected to yield important insights in the effects of online CBT. In this RCT, the online treatment is designed to be as similar as possible to face-face CBT in terms of treatment content, treatment duration, and experience and training of therapists. When we find effect sizes for online CBT that are similar to effect sizes found in earlier studies for face-to-face CBT, delivering CBT online can be considered as supplement to face-to-face treatment, in particular when barriers to face-to-face treatments, such as waiting lists and travel expenses, are experienced.

We will also examine potential correlates of change. These analyses, examining the associations between reductions in symptoms levels and among others negative cognitions and avoidance behaviors, will provide insights in potential underlying therapeutic processes to foster recovery from traumatic loss. These insights are deemed important to design

For peer review only - http://bmjopen.bmj.com/site/about/guidelines.xhtml

treatments that more effectively target these correlates of change. We also expect to improve our knowledge on for whom (e.g., women or people who are more remotely bereaved) grief-specific CBT works best. Findings on these potential correlates of change are necessary to improve treatments given that a maximum of 42% of bereaved people report clinically relevant reductions in grief levels after treatment(21).

Lastly, the role of therapeutic alliance on therapy outcomes will be explored. Prior research in bereaved people has shown that greater therapeutic alliance, from the perspective of the client, at week 4 of a face-to-face grief-specific treatment, was related to greater reductions in grief levels. This therapeutic alliance-grief relationship was not significant for a non-grief specific treatment(60). Our exploration of this association, from the perspective of client *and* therapist, may for the first time shed light on therapeutic processes in online CBT for traumatic grief.

An anticipated limitation of our RCT is the self-selected sample. It is possible that people who are more open towards innovative technology in general(61) and who received support prior to the loss(32) are more likely to sign up for this study, limiting the generalizability of findings emerging from this study. Due to the absence of an active control group (e.g., face-to-face CBT) we are not able to test the effects of online CBT compared with an alternative treatment. Furthermore, we will use self-report measures instead of diagnostic interviews, which may increase the risk of overestimating symptom levels(62). Another potential limitation of this trial relates to the fact that the operationalization and assessment of grief as a disorder is still under debate(63–65). For instance, PCBD, included as "condition for further study" in the DSM-5, is likely to be changed in a revision of the DSM. To maximize diagnostic compatibility, we added four items, corresponding to ICD-11 PGD

For peer review only - http://bmjopen.bmj.com/site/about/guidelines.xhtml

criteria, to the TGI-SR, enabling operationalizion of our primary outcome measure in terms of diagnoses of pathological grief according to both the DSM-5 and the ICD-11.

To conclude, this RCT will provide new insights in effectiveness of online CBT for people who experience clinically relevant distress after bereavement due to a traffic accident, as well as in potential correlates of therapeutic change. As trials to date have primarily focused on effects of face-to-face treatment for non-traumatically bereaved people, our findings are expected to provide a valuable addition to the knowledge base on treating severely distressed bereaved people.

For peer review only

For peer review only - http://bmjopen.bmj.com/site/about/guidelines.xhtml

References

1. World Health Organization. The top 10 causes of death [Internet]. 2019 [geciteerd 14 oktober 2019]. Beschikbaar op: https://www.who.int/news-room/fact-sheets/detail/the-top-10-causes-of-death

2. Lundorff M, Holmgren H, Zachariae R, Farver-Vestergaard I, O'Connor M. Prevalence of prolonged grief disorder in adult bereavement: A systematic review and meta-analysis. J Affect Disord. 2017;212:138–49.

3. Onrust SA, Cuijpers P. Mood and anxiety disorders in widowhood: a systematic review. Aging Ment Health. 2006;10(4):327–34.

4. American Psychiatric Association. Diagnostic and statistical manual of mental disorders (5th ed.). Arlington, VA: American Psychiatric Publishing; 2013.

5. Boelen PA, van de Schoot R, van den Hout MA, de Keijser J, van den Bout J. Prolonged Grief Disorder, depression, and posttraumatic stress disorder are distinguishable syndromes. J Affect Disord. 2010;125(1–3):374–8.

6. Lenferink LIM, Nickerson A, de Keijser J, Smid GE, Boelen PA. Trajectories of grief, depression, and posttraumatic stress in disaster-bereaved people. Depress Anxiety. 2018;

7. Malgaroli M, Maccallum F, Bonanno GA. Symptoms of persistent complex bereavement disorder, depression, and PTSD in a conjugally bereaved sample: a network analysis. Psychol Med. 2018;48(14):2439–48.

For peer review only

For peer review only - http://bmjopen.bmj.com/site/about/guidelines.xhtml

8. Heeke C, Kampisiou C, Niemeyer H, Knaevelsrud C. A systematic review and meta-analysis of correlates of prolonged grief disorder in adults exposed to violent loss. Eur J Psychotraumatology [Internet]. 2019 [geciteerd 14 oktober 2019];10(1). Beschikbaar op: https://www.ncbi.nlm.nih.gov/pmc/articles/PMC6442112/

9. Kristensen P, Weisæth L, Heir T. Bereavement and mental health after sudden and violent losses: a review. Psychiatry. 2012;75(1):76–97.

10. Eisma, M.C., de Keijser, J. Nabestaanden van verkeersongevallen. In: de Keijser, J., Boelen, P.A., Smid, G.E., redacteuren. Handboek traumatische rouw. Amsterdam: Uitgeverij Boom; 2018. p. 104–16.

11. Boelen PA, de Keijser J, Smid G. Cognitive–behavioral variables mediate the impact of violent loss on post-loss psychopathology. Psychol Trauma Theory Res Pract Policy. 2015;7(4):382–90.

12. Boelen PA, van den Hout MA, van den Bout J. A Cognitive-Behavioral Conceptualization of Complicated Grief. Clin Psychol Sci Pract. 2006;13(2):109–28.

13. Janoff-Bulman R. Assumptive worlds and the stress of traumatic events: Applications of the schema construct. Soc Cogn. 1989;7(2):113–36.

14. Boelen PA, Lensvelt-Mulders GJLM. Psychometric Properties of the Grief Cognitions Questionnaire (GCQ). J Psychopathol Behav Assess. 2005;27(4):291–303.

15. Foa EB, Riggs DS, Massie ED, Yarczower M. The impact of fear activation and anger on the efficacy of exposure treatment for posttraumatic stress disorder. Behav Ther. 1995;26(3):487–99.

For peer review only - http://bmjopen.bmj.com/site/about/guidelines.xhtml

For peer review only

16. Tehrani, N. Road victim trauma: an investigation of the impact on the injured and bereaved. Couns Psychol Q. 2004;17(4):361–73.

17. Boelen PA. A Sense of 'unrealness' about the death of a loved-one: An exploratory study of its role in emotional complications among bereaved individuals. Appl Cogn Psychol. 2010;24(2):238–51.

18. Boelen PA. "It feels as if she might return one day": A sense of unrealness as a predictor of bereavement-related emotional distress / "Tengo la sensación de que ella puede volver algún día": la sensación de irrealidad como un predictor del sufrimiento emocional relacionado con la pérdida. Estud Psicol. 2017;38(3):734–51.

19. Benight CC. Social cognitive theory of posttraumatic recovery: the role of perceived self-efficacy. Behav Res Ther. 2004;42(10):1129–48.

20. Smid GE, Kleber RJ, Rie SM de la, Bos JBA, Gersons BPR, Boelen PA. Brief Eclectic Psychotherapy for Traumatic Grief (BEP-TG): toward integrated treatment of symptoms related to traumatic loss. Eur J Psychotraumatology. 2015;6(1):27324.

21. Boelen PA, Smid GE. Disturbed grief: prolonged grief disorder and persistent complex bereavement disorder. BMJ. 2017;357:j2016.

22. Currier JM, Holland JM, Neimeyer RA. Do CBT-Based Interventions Alleviate Distress Following Bereavement? A Review of the Current Evidence. International Journal of Cognitive Therapy. 2010;3:77–93.

23. Doering BK, Eisma MC. Treatment for complicated grief: state of the science and ways forward. Curr Opin Psychiatry. 2016;29(5):286–91.

For peer review only - http://bmjopen.bmj.com/site/about/guidelines.xhtml

24. Johannsen M, Damholdt MF, Zachariae R, Lundorff M, Farver-Vestergaard I, O'Connor M. Psychological interventions for grief in adults: A systematic review and meta-analysis of randomized controlled trials. J Affect Disord. 2019;253:69–86.

25. Boelen PA, Keijser J de, Hout MA van den, Bout J van den. Factors associated with outcome of cognitive–behavioural therapy for complicated grief: A preliminary study. Clin Psychol Psychother. 2011;18(4):284–91.

26. Bryant RA, Kenny L, Joscelyne A, Rawson N, Maccallum F, Cahill C, e.a. Predictors of treatment response for cognitive behaviour therapy for prolonged grief disorder. Eur J Psychotraumatology [Internet]. 2019 [geciteerd 13 oktober 2019];8(6). Beschikbaar op: https://www.ncbi.nlm.nih.gov/pmc/articles/PMC6383610/

27. Linde K, Treml J, Steinig J, Nagl M, Kersting A. Grief interventions for people bereaved by suicide: A systematic review. PloS One. 2017;12(6):e0179496.

28. Eisma MC, Boelen PA, van den Bout J, Stroebe W, Schut HAW, Lancee J, e.a. Internet-Based Exposure and Behavioral Activation for Complicated Grief and Rumination: A Randomized Controlled Trial. Behav Ther. 2015;46(6):729–48.

29. Kersting A, Dölemeyer R, Steinig J, Walter F, Kroker K, Baust K, e.a. Brief Internet-based intervention reduces posttraumatic stress and prolonged grief in parents after the loss of a child during pregnancy: a randomized controlled trial. Psychother Psychosom. 2013;82(6):372–81.

30. Wagner B, Knaevelsrud C, Maercker A. Internet-based cognitive-behavioral therapy for complicated grief: a randomized controlled trial. Death Stud. 2006;30(5):429–53.

For peer review only - http://bmjopen.bmj.com/site/about/guidelines.xhtml

31. Baumeister H, Reichler L, Munzinger M, Lin J. The impact of guidance on Internet-based mental health interventions — A systematic review. Internet Interv. 2014;1(4):205–15.

32. Lichtenthal WG, Corner GW, Sweeney CR, Wiener L, Roberts KE, Baser RE, e.a. Mental Health Services for Parents Who Lost a Child to Cancer: If We Build Them, Will They Come? J Clin Oncol Off J Am Soc Clin Oncol. 2015;33(20):2246–53.

33. Andersson G, Titov N. Advantages and limitations of Internet-based interventions for common mental disorders. World Psychiatry. 2014;13(1):4–11.

34. Eisma MC, Boelen PA, Lenferink LIM. Prolonged grief disorder following the Coronavirus (COVID-19) pandemic. Psychiatry Res. 2020;288:113031.

35. Melville KM, Casey LM, Kavanagh DJ. Dropout from Internet-based treatment for psychological disorders. Br J Clin Psychol. 2010;49(Pt 4):455–71.

36. Pihlaja S, Stenberg J-H, Joutsenniemi K, Mehik H, Ritola V, Joffe G. Therapeutic alliance in guided internet therapy programs for depression and anxiety disorders - A systematic review. Internet Interv. 2018;11:1–10.

37. Bordin ES. Theory and research on the therapeutic working alliance: New directions. In: The working alliance: Theory, research, and practice. Oxford, England: John Wiley & Sons; 1994. p. 13–37. (Wiley series on personality processes).

38. Cook JE, Doyle C. Working alliance in online therapy as compared to face-to-face therapy: preliminary results. Cyberpsychology Behav Impact Internet Multimed Virtual Real Behav Soc. 2002;5(2):95–105.

For peer review only

For peer review only - http://bmjopen.bmj.com/site/about/guidelines.xhtml

39. Sucala M, Schnur JB, Constantino MJ, Miller SJ, Brackman EH, Montgomery GH. The therapeutic relationship in e-therapy for mental health: A systematic review. J Med Internet Res. 2012;14(4):e110.

40. van Denderen M, de Keijser J, Stewart R, Boelen PA. Treating complicated grief and posttraumatic stress in homicidally bereaved individuals: A randomized controlled trial. Clin Psychol Psychother. 2018;

41. Lenferink LIM, de Keijser J, Wessel I, Boelen PA. Cognitive behavioural therapy and mindfulness for relatives of missing persons: A pilot study. Pilot Feasibility Stud. 2019;5(1):93.

42. Boelen PA, Bout J van den. Protocollaire behandeling van persisterende complexe rouwstoornis. In: Keijsers, G., Minnen, A. van, Verbraak, M., Hoogduin, K., Emmelkamp, P., redacteuren. Protocollaire behandelingen voor volwassenen met psychische klachten. 1ste dr. Amsterdam: Boom; 2017.

43. Boelen PA, Smid GE. The Traumatic Grief Inventory Self-Report Version (TGI-SR): Introduction and Preliminary Psychometric Evaluation. J Loss Trauma. 2017;22(3):196–212.

44. World Health Organization. ICD-11. Prolonged Grief Disorder Criteria [Internet]. 2018 [geciteerd 14 oktober 2019]. Beschikbaar op: https://icd.who.int/browse11/l-m/en#/http://id.who.int/icd/entity/1183832314

For peer review only - http://bmjopen.bmj.com/site/about/guidelines.xhtml

45. Boelen PA, Djelantik AAAMJ, de Keijser J, Lenferink LIM, Smid GE. Further validation of the Traumatic Grief Inventory-Self Report (TGI-SR): A measure of persistent complex bereavement disorder and prolonged grief disorder. Death Stud. 2019;43(6):351–64.

46. Blevins CA, Weathers FW, Davis MT, Witte TK, Domino JL. The Posttraumatic Stress Disorder Checklist for DSM-5 (PCL-5): Development and Initial Psychometric Evaluation. J Trauma Stress. 2015;28(6):489–98.

47. Boeschoten, M.A., Bakker, A., Jongedijk, R.A., Olff, M. PTSS checklist voor de DSM-5 (PCL-5). Diemen: Arq Nationaal Psychotrauma Centrum; 2014.

48. Zigmond AS, Snaith RP. The hospital anxiety and depression scale. Acta Psychiatr Scand. 1983;67(6):361–70.

49. Spinhoven PH, Ormel J, Sloekers PPA, Kempen GIJM. A validation study of the Hospital Anxiety and Depression scale (HADS) in different groups of Dutch subjects. Psychol Med. 1997;27(2):363–70.

50. Boelen PA, van den Bout J. Anxious and depressive avoidance and symptoms of prolonged grief, depression, and post-traumatic stress disorder. Psychol Belg. 2010;50(1–2):49–67.

51. Spielberger, C.D. State-Trait Anger Expression Inventory-2. Psychological Assessment Resources; 1999.

52. Lievaart M, Franken IHA, Hovens JE. Anger Assessment in Clinical and Nonclinical Populations: Further Validation of the State-Trait Anger Expression Inventory-2. J Clin Psychol. 2016;72(3):263–78.

For peer review only

For peer review only - http://bmjopen.bmj.com/site/about/guidelines.xhtml

53. Schwarzer, R., Jerusalem, M. Generalized self-efficacy scale. In: Weinman, J., Wright, S., Johnston, M., redacteuren. Measures in health psychology: A user's portfolio Causal and control beliefs. Windsor, UK: Nfer-Nelson; 1995. p. 35–7.

54. Horvath AO, Greenberg LS. Development and validation of the Working Alliance Inventory. J Couns Psychol. 1989;36(2):223–33.

55. Vertommen, H., Vervaeke, G. Nederlandstalige experimentele vertaling van de Working Alliance Inventory van Horvath en Greenberg. Leuven: KU; 1990.

56. Munder T, Wilmers F, Leonhart R, Linster HW, Barth J. Working Alliance Inventory-Short Revised (WAI-SR): psychometric properties in outpatients and inpatients. Clin Psychol Psychother. 2010;17(3):231–9.

57. Snijders, T.A.B., Bosker, R.J. Deviance tests. In: Snijders, T.A.B., Bosker, R.J., redacteuren. Multilevel analysis. London: SAGE Publications Ltd; 2012. p. 97–8.

58. Jacobson NS, Truax P. Clinical significance: A statistical approach to defining meaningful change in psychotherapy research. J Consult Clin Psychol. 1991;59(1):12–9.

59. Lenferink LIM, de Keijser J, Smid GE, Boelen PA. Cognitive therapy and EMDR for reducing psychopathology in bereaved people after the MH17 plane crash: Findings from a randomized controlled trial. Traumatology. 2020;

60. Glickman K, Shear, K.M., Wall MM. Therapeutic Alliance and Outcome in Complicated Grief Treatment. Int J Cogn Ther. 2018;11(2):222–33.

61. Arjadi R, Nauta MH, Bockting CLH. Acceptability of internet-based interventions for depression in Indonesia. Internet Interv. 2018;13:8–15.

For peer review only - http://bmjopen.bmj.com/site/about/guidelines.xhtml

62. Lim GY, Tam WW, Lu Y, Ho CS, Zhang MW, Ho RC. Prevalence of Depression in the Community from 30 Countries between 1994 and 2014. Sci Rep. 2018;8(1):2861.

63. Lenferink, L.I.M., Boelen, P.A., Smid, G.E., Paap, M.C.S. The importance of harmonising diagnostic criteria sets for pathological grief. British Journal of Psychiatry. in press;

64. Maciejewski PK, Prigerson HG. Prolonged, but not complicated, grief is a mental disorder. Br J Psychiatry. 2017;211(4):189–91.

65. Wakefield JC. Should prolonged grief be reclassified as a mental disorder in DSM-5?: reconsidering the empirical and conceptual arguments for complicated grief disorder. J Nerv Ment Dis. 2012;200(6):499–511.

Authors' contributions

JdK is principal investigator. LL is executive researcher. JdK, PB, ME, and GS are grant holders. LL developed the study design and wrote the ethics proposal and drafts of the manuscript. JdK, ME, GS, and PB read, revised, and approved the drafts of the study design, ethics proposal, and the manuscript.

Funding statement

Fund Victim Support subsidized this work.

Competing Interests Statement

All authors declare to have no competing interests.

For peer review only

Figure caption/legend

Figure 1. Design of RCT

Note. CBT = cognitive behavioral therapy

For peer review only

For peer review only - http://bmjopen.bmj.com/site/about/guidelines.xhtml

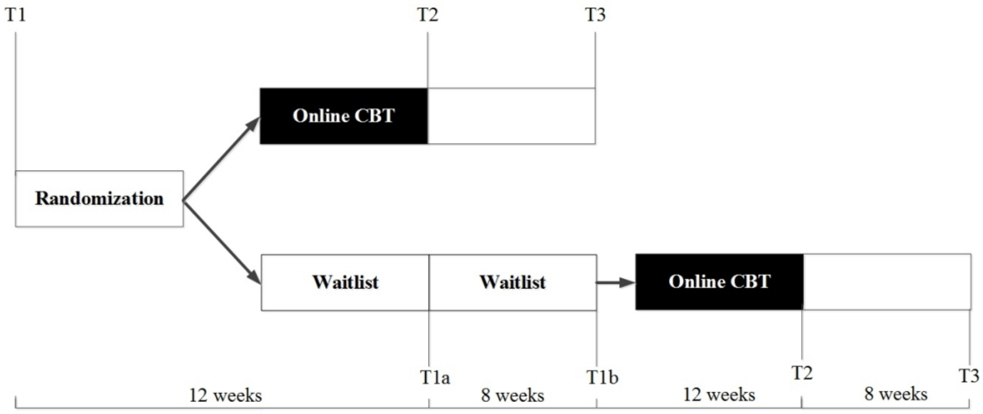

Figure 1. Design of RCT

356x148mm (96 x 96 DPI)

Supplementary File

Information for participants

Subject: Participation in study on the treatment of grief after a traffic accident

Dear sir/madam,

We ask you to take part in a scientific study. This participation is voluntary. In order to take part we do need your permission. You receive this information because you filled in a questionnaire some time ago, mapping the emotional consequences of the death of a loved one due to a traffic accident. You have indicated that you may be interested in taking part in a follow-up study exploring the effects of a treatment to learn to cope better with the death of your loved one.

Before you decide whether you want to take part in this study, you receive an explanation of what the study entails. Read this information carefully and ask the researcher to explain if you have questions.

1. **General Information**

This study is conducted by the University of Groningen, Utrecht University and Stichting Centrum '45. The study is financed by the Fonds Slachtofferhulp (Victim Support Fund). The study consists of participation in an online psychological treatment. The treatment is provided by therapists in various practices across the Netherlands. The medical-ethical review committee of the Universitair Medisch Centrum Groningen (Academic Medical Centre Groningen) has approved this study.

2. **Purpose of the study**

Those left behind after a traffic accident often indicate that the help provided does not sufficiently connect to their experiences. Therefore a treatment has been developed that is specifically intended for the partner, relatives and friends of someone who died due to a traffic accident. You can discuss with the therapist how you are coping with the loss and what the consequences of this loss are in your life. The aim of the treatment is to cope better with the loss. The treatment is part of a scientific study. The purpose of the study is to explore whether treatment leads to a reduction in emotional problems for those who lost someone due to a traffic accident.

3. **Background of the study**

During the treatment cognitive behavioural therapy will be used. Previous research has shown that cognitive behavioural therapy is the most effective treatment for reducing emotional problems after the loss of a loved one due to a natural cause (for instance illness). Cognitive behavioural therapy is mainly applied during individual sessions with a therapist (face-to-face treatment). Research has shown that cognitive behavioural therapy, offered via the internet (online treatment), also seems to be suitable for reducing problems after a natural death. More research is needed to find out whether this online version of cognitive behavioural therapy is also suitable for those who lost someone due to a traffic accident. The purpose of this study is to find out whether online cognitive behavioural therapy is accompanied by a reduction of emotional problems after the death of a loved one due to a traffic accident.

The online treatment is provided individually and consists of eight modules which you go through in twelve weeks. You will then have online contact with a therapist who will guide you during the treatment. In the Netherlands, a network of therapists has been trained in the online treatment of people

For peer review only

who lost someone due to a traffic accident. The therapists work at several treatment centres. The online treatment is offered by Therapieland. Therapieland is a provider of psychological care via the internet.

4. What participation entails

If you take part, this will take at least 20 weeks in total for you.

Screening

First we will determine whether you can take part. You will be asked to fill in a questionnaire. The questionnaire contains questions about emotional problems you may experience in response to the passing away of your loved one due to a traffic accident. Also, questions are asked about previous psychological help you may have received. The questionnaire is used to get a picture of the degree to which you experience emotional problems.

If your completed questionnaire shows that you experience relatively few emotional problems, you cannot take part in the study. You also cannot take part in the study if you have no access to the internet. If your answers show that the treatment offered is not suitable for you, an alternative treatment will be looked for in consultation with you.

It is possible that you filled out a questionnaire on this topic before. As problems can change over time, we ask you to fill in a questionnaire once more. In this way we get a picture of the problems you are experiencing at the moment.

Treatment

In order to be able to determine the effect of the treatment, participants are assigned to one of two groups. The first group will start with the online treatment as soon as possible after registration. The second group will start with the online treatment after a waiting period of 20 weeks. By adding a waitlist group it can be determined that a reduction of problems is actually the result of the treatment, and not of the passage of time. Which group you are assigned to will be determined by drawing lots. You and we do not have any influence on the draw. We will let you know when your treatment starts.

Measurements

Before the treatment can start, you will be asked to fill in a questionnaire. This questionnaire will focus on the problems you experience. We map these in order to be able to determine whether the treatment might help you.

The therapist who will guide your online treatment will be informed of the results of this questionnaire beforehand. In order to determine to what extent the treatment helps you, we ask you to fill in a questionnaire not only beforehand, but also once during the treatment, after the last treatment session, and 8 weeks after the treatment. People assigned to the waiting group will be asked to fill in an additional questionnaire after 12 weeks and 20 weeks of waiting. In this way it can be determined whether the treatment has been effective and what the short and long term effects of the treatment are. The filling in will take approximately 10 to 30 minutes per measurement.

5. What is expected from you

In order to ensure that the study runs smoothly it is important that you adhere to the following agreements. These agreements are that you:

- contact the researcher in case of problems (the contact details are listed at the end of the information letter);
- keep all appointments with your therapist;
- fill in the questionnaires before and after the treatment.

For peer review only

Besides, it is important that you contact the researcher if:

- your contact information changes
- you no longer wish to take part in the study

6. Possible negative effects

During the treatment you will actively engage with your thoughts and feelings about the loss. It is possible that feelings such as grief or loss or fatigue may temporarily increase in intensity. Filling in the questionnaire can also evoke emotional responses. When your problems increase to a great extent, you can contact the researcher via the contact details listed at the end of this letter.

7. Possible advantages and disadvantages

It is important that you carefully weigh the possible advantages and disadvantages before you decide to take part. The treatment may reduce your emotional problems, but this is not certain. Your participation will contribute to more knowledge about the treatment of emotional problems after the death of a loved one due to a traffic accident.

Disadvantages of taking part in the study may be:

- possible worsening of problems due to taking part in the treatment;
- possible worsening of problems due to filling in the questionnaire.

Participation in the study also means that:

- the study will cost you time;
- you will have to adhere to certain agreements.

These issues have all been described in section 4, 5 and 6 above.

8. If you do not wish to participate or if you wish to stop participating in the study

You decide whether or not to take part in the study. Participation is voluntary. If you do take part, you can always change your mind and stop participating after all, during the study as well. You do not have to give a reason for stopping. You do have to report this to the researcher immediately. The data collected up to that point will be used for the study.

If there is new information about the study that is important to you, the researcher will let you know this. We will then ask you if you continue to participate.

9. End of the study

Your participation in the study will end when: you will have filled in the questionnaire 8 weeks after the end of the treatment; you make the choice to stop participating; the researcher considers it better for you to stop participating; the government or the reviewing medical-ethical committee decide to end the study. The entire study will end when all participants are done. After processing all the data, the researcher will inform you of the most important outcomes of the study by means of a newsletter. You can indicate whether you wish to receive this newsletter at the end of the questionnaire.

For peer review only

10. Use and storage of your data

For this study your personal data will be collected, used and stored. This concerns data such as your name, address, date of birth and data related to your health. The collection, use and storage of your data is necessary to be able to answer the questions asked in this study and to publish the results. We ask your permission for the use of your data.

Confidentiality of your data

In order to protect your privacy, your data will get a code. Your name and other data which can identify you directly will be left out from this. Data can only be traced to you with the key to the code. The key to the code will remain safely stored at the local research institution. The data sent to eventual other parties involved only contain the code, but not your name or other data with which you can be identified. In reports and publications on the study the data cannot be traced to you either.

Access to your data for checks

Some people may get access to all your data at the research location, including the data without code. This is necessary to be able to check whether the study has been done properly and reliably. People who will gain access to you data for checking purposes will be the researchers Lonneke Lenferink, a research assistant and Jos de Keijser, the committee monitoring the safety of the study and international supervising authorities. They will keep your data secret. We ask you to give your permission for this access.

Data retention period

Your data must be retained at the research location for 15 years. They are stored in order to be able to make new provisions related to this study in the course of this study.

Withdrawal of permission

You can always withdraw your permission for the use of your personal data again. The research data collected up to the moment you withdraw permission will still be used in the study.

Further information about your rights regarding data processing

For general information about you rights you can contact the person responsible for processing your personal data. For this study this is Lonneke Lenferink (University of Groningen). In case of questions or complaints regarding the processing of your personal data we recommend that you contact her.

11. Insurance for test subjects

An insurance has been taken out for everyone taking part in the study. The insurance covers damage from the study. You can report damage to the researchers.

12. Informing family doctor/GP

We do not share information about your participation in the study with your family doctor/GP.

13. Compensation for participation

For peer review only

Participation in the online treatment is free of charge.

14. Do you have any questions?

In case of general questions regarding the study you can contact the researcher assistant. If you have complaints about the study, you can discuss this with the researchers or with your treating therapist. If you prefer not to do this, you can contact the University of Groningen.

15. Signing the consent form

Below you find a 'Declaration of consent for participation in research into the treatment of grief after a traffic accident', on which you can indicate whether you wish to take part in the study. With your permission you indicate that you have understood the information and that you agree to participate in the study.

After you have filled in this declaration, you can start filling in the questionnaire. Approximately within four weeks after filling in the questionnaire we will let you know by email or by phone whether you qualify for the treatment. When you qualify for treatment, you will receive the outcome of the draw which indicates whether you have been assigned to a group that can start with the online treatment as soon as possible, or whether you have been assigned to the group that needs to wait for 20 weeks before the start of the online treatment.

If you have any further questions, you can contact us via the details below.

Kind regards,

[name research assistant]

Research assistant University of Groningen

Lonneke Lenferink

Post-doctoral researcher University of Groningen and Utrecht University

Contact Details

General questions about the study: [name research assistant], info@rouwnaverkeersongeval.nl

Questions regarding the protection of your data, your rights or complaints: please contact Lonneke Lenferink, l.i.m.lenferink@rug.nl.

For peer review only

Consent Form
for participation in research on treatment of grief after a traffic accident

- I have read the information letter. I also had the opportunity to ask questions. My questions have been answered adequately. I had enough time to decide whether to participate or not.
- I know that participation is voluntary. I also know that I can decide at any moment not to participate after all or to stop participating in the study. I do not have to give a reason for this.
- I give permission for collecting and using my data to answer the research question of this study.
- I know that some people may gain access to all of my data for the purpose of checks of this study. Those people are listed in this information letter. I give permission for this access by these people.
- I wish to participate in this study.

Name of participant:

Email address:

Phone number:

Signature of participant: Date: __ / __ / __

The researcher, Lonneke Lenferink, hereby declares that the test subject has been fully informed about the aforementioned study.

If information becomes known during the study which might influence the consent of the participant, the participant will be timely informed about this.

For peer review only

Reporting checklist for protocol of a clinical trial.

Based on the SPIRIT guidelines.

Instructions to authors

Complete this checklist by entering the page numbers from your manuscript where readers will find each of the items listed below.

Your article may not currently address all the items on the checklist. Please modify your text to include the missing information. If you are certain that an item does not apply, please write "n/a" and provide a short explanation.

Upload your completed checklist as an extra file when you submit to a journal.

In your methods section, say that you used the SPIRITreporting guidelines, and cite them as:

Chan A-W, Tetzlaff JM, Altman DG, Laupacis A, Gøtzsche PC, Krleža-Jerić K, Hróbjartsson A, Mann H, Dickersin K, Berlin J, Doré C, Parulekar W, Summerskill W, Groves T, Schulz K, Sox H, Rockhold FW, Rennie D, Moher D. SPIRIT 2013 Statement: Defining standard protocol items for clinical trials. Ann Intern Med. 2013;158(3):200-207

		Reporting Item	Page Number
**Administrative information**			
Title	#1	Descriptive title identifying the study design, population, interventions, and, if applicable, trial acronym	1

For peer review only

Trial registration	#2a	Trial identifier and registry name. If not yet registered, name of intended registry	3
Trial registration: data set	#2b	All items from the World Health Organization Trial Registration Data Set	n/a
Protocol version	#3	Date and version identifier	n/a
Funding	#4	Sources and types of financial, material, and other support	32
Roles and responsibilities: contributorship	#5a	Names, affiliations, and roles of protocol contributors	32
Roles and responsibilities: sponsor contact information	#5b	Name and contact information for the trial sponsor	n/a
Roles and responsibilities: sponsor and funder	#5c	Role of study sponsor and funders, if any, in study design; collection, management, analysis, and interpretation of data; writing of the report; and the decision to submit the report for publication, including whether they will have ultimate authority over any of these activities	32
Roles and responsibilities: committees	#5d	Composition, roles, and responsibilities of the coordinating centre, steering committee, endpoint adjudication committee, data management team, and	n/a

For peer review only

		other individuals or groups overseeing the trial, if applicable (see Item 21a for data monitoring committee)	
**Introduction**			
Background and rationale	#6a	Description of research question and justification for undertaking the trial, including summary of relevant studies (published and unpublished) examining benefits and harms for each intervention	4-7
Background and rationale: choice of comparators	#6b	Explanation for choice of comparators	4-7
Objectives	#7	Specific objectives or hypotheses	8
Trial design	#8	Description of trial design including type of trial (eg, parallel group, crossover, factorial, single group), allocation ratio, and framework (eg, superiority, equivalence, non-inferiority, exploratory)	8-9
**Methods: Participants, interventions, and outcomes**			
Study setting	#9	Description of study settings (eg, community clinic, academic hospital) and list of countries where data will be collected. Reference to where list of study sites can be obtained	10

For peer review only

Eligibility criteria	#10	Inclusion and exclusion criteria for participants. If applicable, eligibility criteria for study centres and individuals who will perform the interventions (eg, surgeons, psychotherapists)	11
Interventions: description	#11a	Interventions for each group with sufficient detail to allow replication, including how and when they will be administered	12-13
Interventions: modifications	#11b	Criteria for discontinuing or modifying allocated interventions for a given trial participant (eg, drug dose change in response to harms, participant request, or improving / worsening disease)	19
Interventions: adherance	#11c	Strategies to improve adherence to intervention protocols, and any procedures for monitoring adherence (eg, drug tablet return; laboratory tests)	10
Interventions: concomitant care	#11d	Relevant concomitant care and interventions that are permitted or prohibited during the trial	16-17
Outcomes	#12	Primary, secondary, and other outcomes, including the specific measurement variable (eg, systolic blood pressure), analysis metric (eg, change from baseline, final value, time to event), method of aggregation (eg, median, proportion), and time point for each outcome. Explanation of the clinical relevance of chosen efficacy and harm outcomes is strongly recommended	14-17

For peer review only

Participant timeline	#13	Time schedule of enrolment, interventions (including any run-ins and washouts), assessments, and visits for participants. A schematic diagram is highly recommended (see Figure)	10
Sample size	#14	Estimated number of participants needed to achieve study objectives and how it was determined, including clinical and statistical assumptions supporting any sample size calculations	11
Recruitment	#15	Strategies for achieving adequate participant enrolment to reach target sample size	10
**Methods: Assignment of interventions (for controlled trials)**			
Allocation: sequence generation	#16a	Method of generating the allocation sequence (eg, computer-generated random numbers), and list of any factors for stratification. To reduce predictability of a random sequence, details of any planned restriction (eg, blocking) should be provided in a separate document that is unavailable to those who enrol participants or assign interventions	8-9
Allocation concealment mechanism	#16b	Mechanism of implementing the allocation sequence (eg, central telephone; sequentially numbered, opaque,	8-9

For peer review only

		sealed envelopes), describing any steps to conceal the sequence until interventions are assigned	
Allocation: implementation	#16c	Who will generate the allocation sequence, who will enrol participants, and who will assign participants to interventions	9
Blinding (masking)	#17a	Who will be blinded after assignment to interventions (eg, trial participants, care providers, outcome assessors, data analysts), and how	9
Blinding (masking): emergency unblinding	#17b	If blinded, circumstances under which unblinding is permissible, and procedure for revealing a participant's allocated intervention during the trial	n/a
**Methods: Data collection, management, and analysis**			
Data collection plan	#18a	Plans for assessment and collection of outcome, baseline, and other trial data, including any related processes to promote data quality (eg, duplicate measurements, training of assessors) and a description of study instruments (eg, questionnaires, laboratory tests) along with their reliability and validity, if known. Reference to where data collection forms can be found, if not in the protocol	14-17

For peer review only

Data collection plan: retention	#18b	Plans to promote participant retention and complete follow-up, including list of any outcome data to be collected for participants who discontinue or deviate from intervention protocols	n/a
Data management	#19	Plans for data entry, coding, security, and storage, including any related processes to promote data quality (eg, double data entry; range checks for data values). Reference to where details of data management procedures can be found, if not in the protocol	19
Statistics: outcomes	#20a	Statistical methods for analysing primary and secondary outcomes. Reference to where other details of the statistical analysis plan can be found, if not in the protocol	17-20
Statistics: additional analyses	#20b	Methods for any additional analyses (eg, subgroup and adjusted analyses)	17-20
Statistics: analysis population and missing data	#20c	Definition of analysis population relating to protocol non-adherence (eg, as randomised analysis), and any statistical methods to handle missing data (eg, multiple imputation)	17-20

Methods: Monitoring

Data monitoring: formal committee	#21a	Composition of data monitoring committee (DMC); summary of its role and reporting structure; statement of whether it is independent from the sponsor and competing interests; and reference to where further	19

For peer review only

		details about its charter can be found, if not in the protocol. Alternatively, an explanation of why a DMC is not needed	
Data monitoring: interim analysis	#21b	Description of any interim analyses and stopping guidelines, including who will have access to these interim results and make the final decision to terminate the trial	19
Harms	#22	Plans for collecting, assessing, reporting, and managing solicited and spontaneously reported adverse events and other unintended effects of trial interventions or trial conduct	19
Auditing	#23	Frequency and procedures for auditing trial conduct, if any, and whether the process will be independent from investigators and the sponsor	19
**Ethics and dissemination**			
Research ethics approval	#24	Plans for seeking research ethics committee / institutional review board (REC / IRB) approval	19
Protocol amendments	#25	Plans for communicating important protocol modifications (eg, changes to eligibility criteria, outcomes, analyses) to relevant parties (eg, investigators, REC / IRBs, trial participants, trial registries, journals, regulators)	19

For peer review only

Consent or assent	#26a	Who will obtain informed consent or assent from potential trial participants or authorised surrogates, and how (see Item 32)	19
Consent or assent: ancillary studies	#26b	Additional consent provisions for collection and use of participant data and biological specimens in ancillary studies, if applicable	n/a
Confidentiality	#27	How personal information about potential and enrolled participants will be collected, shared, and maintained in order to protect confidentiality before, during, and after the trial	19
Declaration of interests	#28	Financial and other competing interests for principal investigators for the overall trial and each study site	32
Data access	#29	Statement of who will have access to the final trial dataset, and disclosure of contractual agreements that limit such access for investigators	19
Ancillary and post trial care	#30	Provisions, if any, for ancillary and post-trial care, and for compensation to those who suffer harm from trial participation	19
Dissemination policy: trial results	#31a	Plans for investigators and sponsor to communicate trial results to participants, healthcare professionals, the public, and other relevant groups (eg, via publication, reporting in results databases, or other data sharing arrangements), including any publication restrictions	19-20

For peer review only

Dissemination policy: authorship	#31b	Authorship eligibility guidelines and any intended use of professional writers	n/a
Dissemination policy: reproducible research	#31c	Plans, if any, for granting public access to the full protocol, participant-level dataset, and statistical code	n/a
**Appendices**			
Informed consent materials	#32	Model consent form and other related documentation given to participants and authorised surrogates	10
Biological specimens	#33	Plans for collection, laboratory evaluation, and storage of biological specimens for genetic or molecular analysis in the current trial and for future use in ancillary studies, if applicable	n/a

None The SPIRIT checklist is distributed under the terms of the Creative Commons Attribution License CC-BY-ND 3.0. This checklist can be completed online using https://www.goodreports.org/, a tool made by the EQUATOR Network in collaboration with Penelope.ai

For peer review only

BMJ Open

Online cognitive behavioral therapy for traumatically bereaved people: Study protocol for a randomized waitlist-controlled trial

Journal:	*BMJ Open*
Manuscript ID	bmjopen-2019-035050.R2
Article Type:	Protocol
Date Submitted by the Author:	21-Jul-2020
Complete List of Authors:	Lenferink, Lonneke; Rijksuniversiteit Groningen, Clinical Psychology and Experimental Psychopathology; Utrecht University, Clinical Psychology<br>de Keijser, Jos; Rijksuniversiteit Groningen, Clinical Psychology and Experimental Psychopathology<br>Eisma, Maarten; Clinical Psychology and Experimental Psychopathology<br>Smid, Geert; Foundation Centrum '45<br>Boelen, Paul; Utrecht University, Clinical Psychology; Foundation Centrum '45
**Primary Subject Heading**:	Mental health
Secondary Subject Heading:	Mental health, Evidence based practice
Keywords:	PSYCHIATRY, Adult psychiatry < PSYCHIATRY, PUBLIC HEALTH

SCHOLARONE™ Manuscripts

I, the Submitting Author has the right to grant and does grant on behalf of all authors of the Work (as defined in the below author licence), an exclusive licence and/or a non-exclusive licence for contributions from authors who are: i) UK Crown employees; ii) where BMJ has agreed a CC-BY licence shall apply, and/or iii) in accordance with the terms applicable for US Federal Government officers or employees acting as part of their official duties; on a worldwide, perpetual, irrevocable, royalty-free basis to BMJ Publishing Group Ltd ("BMJ") its licensees and where the relevant Journal is co-owned by BMJ to the co-owners of the Journal, to publish the Work in this journal and any other BMJ products and to exploit all rights, as set out in our licence.

The Submitting Author accepts and understands that any supply made under these terms is made by BMJ to the Submitting Author unless you are acting as an employee on behalf of your employer or a postgraduate student of an affiliated institution which is paying any applicable article publishing charge ("APC") for Open Access articles. Where the Submitting Author wishes to make the Work available on an Open Access basis (and intends to pay the relevant APC), the terms of reuse of such Open Access shall be governed by a Creative Commons licence – details of these licences and which Creative Commons licence will apply to this Work are set out in our licence referred to above.

Other than as permitted in any relevant BMJ Author's Self Archiving Policies, I confirm this Work has not been accepted for publication elsewhere, is not being considered for publication elsewhere and does not duplicate material already published. I confirm all authors consent to publication of this Work and authorise the granting of this licence.

For peer review only

Online cognitive behavioral therapy for traumatically bereaved people: Study protocol for a randomized waitlist-controlled trial

Lenferink, L.I.M.*[1,2], de Keijser, J.[1], Eisma, M.C.[1], Smid, G.E.[3,4,5], & Boelen, P.A.[2,3,4]

[1] Department of Clinical Psychology and Experimental Psychopathology, Faculty of Behavioral and Social Sciences, University of Groningen, Grote Kruisstraat 2/1, 9712 TS Groningen, The Netherlands. E-mail L.I.M. Lenferink: l.i.m.lenferink@rug.nl.

[2] Department of Clinical Psychology, Faculty of Social Sciences, Utrecht University, P.O. Box 80140, 3508 TC, Utrecht, The Netherlands.

[3] ARQ Nationaal Psychotrauma Centre, Nienoord 5, 1112 XE, Diemen, The Netherlands

[4] Foundation Centrum ’45, Nienoord 5, 1112 XE Diemen, The Netherlands

[5] University of Humanistic Studies, Utrecht, The Netherlands

*Corresponding author

For peer review only

Abstract

Introduction

The traumatic death of a loved one, such as death due to a traffic accident, can precipitate persistent complex bereavement disorder (PCBD) and comorbid posttraumatic stress disorder (PTSD) and depression. Waitlist-controlled trials have shown that grief-specific cognitive behavioral therapy (CBT) is an effective treatment for such mental health problems. This is the first study that will examine the effectiveness of online CBT (vs. waitlist controls) in a sample exclusively comprised of people bereaved by a traumatic death. Our primary hypothesis is that people allocated to the online CBT condition will show larger reductions in PCBD, PTSD, and depression symptom levels at post-treatment than people allocated to a waitlist. We further expect that reductions in symptom levels during treatment are associated with reductions of negative cognitions and avoidance behaviors and the experience of fewer accident-related stressors. Moreover, the effect of the quality of the therapeutic alliance on treatment effects and drop-out rates will be explored.

Methods and analysis

A two-arm (online CBT vs. waiting list) open label parallel randomized controlled trial (RCT) will be conducted. Participants will complete questionnaires at pre-treatment and 12 and 20 weeks after study enrolment. Eligible for participation are Dutch adults who lost a loved one at least one year earlier due to a traffic accident and report clinically relevant levels of PCBD, PTSD, and/or depression. Multilevel modeling will be used.

Ethics and dissemination

For peer review only - http://bmjopen.bmj.com/site/about/guidelines.xhtml

Ethics approval has been received by the Medical Ethics Review Board of the University Medical Center Groningen (METc UMCG: M20.252121). This study will provide new insights in the effectiveness of online CBT for traumatically bereaved people. If the treatment is demonstrated to be effective, it will be made publicly accessible. Findings will be disseminated among lay people (e.g., through newsletters and media performances), our collaborators (e.g., through presentations at support organizations), and clinicians and researchers (e.g., through conference presentations and scientific journal articles).

Strengths and limitations of this study

- This study is the first to examine the effectiveness of online CBT (vs. waitlist controls) in reducing psychopathology after traumatic loss in an RCT.
- This study is one of the first to examine potential correlates of change in symptom levels following online treatment after traumatic loss.
- We are not able to formally test mediators or moderators of treatment effects.
- We are not able to examine if online CBT has equal effects as face-to-face CBT.
- We are not able to establish formal diagnoses, as we use self-report questionnaires, instead of diagnostic interviews, to assess symptom levels.

Trial registration number: NL7497 (Dutch Trial Register)

Word Count: 5,133 words including main text

For peer review only

Worldwide, traffic accidents represent the leading cause of unnatural deaths(1). Ten to 20 percent of bereaved people who experience natural deaths (e.g., illness) develop severe and persistent grief-related distress, including persistent complex bereavement disorder (PCBD), posttraumatic stress disorder (PTSD), and depression(2,3). Notably, PCBD has been introduced as other specified trauma- and stressor-related disorder, in the latest version of the Diagnostic and Statistical manual of Mental Diseases (DSM-5)(4). PCBD can be diagnosed if, after the death of a significant other at least 12 months earlier, a person experiences persistent yearning for the deceased and symptoms of reactive distress (e.g., emotional numbness) and social/identity disruption (e.g., feeling alone) causing impairment in daily life. While some PCBD symptoms overlap with PTSD (e.g., anger) and depression symptoms (e.g., diminished interest in activities), several studies have shown that these three syndromes are distinct(5–7). Unexpected/violent losses of a significant other, also referred to as a traumatic losses, including deaths caused by traffic accidents, increase risks for the development of PCBD, PTSD, and depression(8,9).

Heightened risk for developing psychopathology after deaths due to traffic accident

Specific circumstances of losses caused by accidents may account for the elevated risk of grief-related distress. For instance, experiencing multiple losses simultaneously, being a witness to the accident, and juridical and financial consequences are proposed to exacerbate grief-related distress(10). Furthermore, negative cognitions and avoidance behaviors may mediate the influence of sudden/violent loss on grief, PTSD, and depression levels(11). According to a cognitive-behavioral model three interacting malleable processes underlie disturbed grief reactions: i) negative cognitions, ii) avoidance behavior, and iii) difficulties integrating the loss into the autobiographical knowledge base(12).

For peer review only - http://bmjopen.bmj.com/site/about/guidelines.xhtml

Experiencing a loss due to a traffic accident may violate basic assumptions about the world being a safe place(13). This may fuel negative cognitions (e.g., “I’m less worthy, since s/he died” and “The death of him/her has taught me that the world is unjust) that may exacerbate and maintain acute grief responses(14). Avoidance behaviors include depressive avoidance and anxious avoidance strategies. Depressive avoidance refers to withdrawal from social and occupational activities that were perceived as fulfilling before the death, out of the conviction that these activities are no longer meaningful. Anxious avoidance strategies serve to prevent confrontation with the reality of the death, out of fear that confrontation is too painful(12). One potential way to avoid confrontation with the reality of the loss, is to focus on angry thoughts and feelings (e.g., “I was angry at the police, courts, or administration, because they did not do their work well enough“)(15). This seems to be a frequently used avoidant coping strategy in bereaved people after traffic accidents and is strongly related to PTSD(16). Difficulty with integration of the loss into the autobiographical knowledge base refers to the difficulties connecting factual knowledge that the loss is irreversible with existing information about the self and the relationship with the lost person, stored in autobiographical memory. Memories related to the loss may lack context in terms of time and place, causing the loss to be experienced as unreal(17). It has been argued that this “sense of unrealness” may trigger intrusive memories and increase feelings of numbness or shock once the bereaved person is confronted with reminders of the loss(17,18). The extent to which a person believes that one is capable of managing stressor-related thoughts, emotions, and behaviors, also referred to as self-efficacy (e.g., “I can usually handle whatever comes my way“), has also been determined as an important factor facilitating coping with traumatic stressors(19). Decreased self-efficacy, negative cognitions

For peer review only - http://bmjopen.bmj.com/site/about/guidelines.xhtml

and insufficient integration of the loss may contribute to increased sensitivity to loss reminders or secondary stressors following traumatic loss(20).

Cognitive-behavioral therapy for grief-related distress

Grief-specific CBT has been demonstrated to be the most effective treatment for bereaved people with elevated grief levels(21–24). CBT targets the abovementioned cognitive-behavioral variables with cognitive restructuring, loss-related exposure, and behavioral activation. Notably, research on putative mechanisms of change of grief-specific CBT is sparse(23) (but see(25,26)). Examining the effectiveness of grief-specific CBT and its potential mechanisms of change in traumatically bereaved people with traumatic grief is clinically relevant because it would enable tailoring of interventions to the specific needs of this group, which could improve treatment outcomes(27).

Whilst the majority of trials assess the efficacy of face-to-face CBT(24), so far, to the best of our knowledge, three online CBT-based interventions have been developed for distressed bereaved people(28–30). These prior studies provided preliminary data on the potential effectiveness of online grief-specific CBT, but had some limitations. For instance, treatment was solely provided to people who experienced perinatal loss (29) or included relatively small samples (28). Comparability between these three studies is also limited, because interventions differed in treatment content; different elements of CBT were offered, for instance behavioral activation, exposure (28), or writing assignments (29,30). Offering CBT via the internet has some potential advantages. It may lower the threshold for seeking treatment, because it can be delivered independent of geographical location. Furthermore, asynchronous communication may be used, allowing the client and therapist can contact each other at any preferred time(31). This may counter barriers to mental health

For peer review only - http://bmjopen.bmj.com/site/about/guidelines.xhtml

service use, such as difficulties with finding help, transportation concerns, or difficulties scheduling treatment sessions(32). In addition, online CBT could reduce treatment costs, improving accessibility and dissemination of care for people in need of support(33). Moreover, during times of a crisis, such as the COVID-19 pandemic, it seems more relevant than ever to further examine the effectiveness of online CBT for distressed bereaved people, as it will allow them to retain access to evidence-based care (34).

A potential downside to online CBT is the high dropout rate found in earlier studies(33,35). It has been argued that a strong therapeutic alliance might support adherence to online treatment and mediates treatment effects (36). Therapeutic alliance is defined as a positive emotional bond between client and therapist, whereby both parties agree on the tasks and goals of the treatment(37). The client-therapist relationship might also explain why online treatments are more effective with therapist guidance than without (31). Concerns have been raised that developing a therapeutic relationship might be more difficult when non-verbal communication is absent(38). However, studies in non-bereaved samples indicate that developing a strong therapeutic alliance is possible during online treatment(33) and that therapeutic alliance is often related to online treatment outcomes(39), but not always (33). More research is needed to further examine the interrelations of the quality of client-therapist relationship, drop-out, and treatment outcomes in online CBT.

Study objectives

Our first aim is to examine the effectiveness of online CBT (vs. a waiting list control condition) in reducing symptom levels of PCBD, PTSD, and depression in people bereaved by a traffic accident. We expect that participants assigned to the online CBT condition will show

For peer review only - http://bmjopen.bmj.com/site/about/guidelines.xhtml

larger reductions in symptom levels of PCBD, PTSD, and depression compared with waitlist controls at post-treatment assessments (Hypothesis 1).

Our second aim is to explore correlates of change. Based on prior research and theories(12,16,19), we expect that reductions in negative cognitions, avoidance behaviors, state anger, a sense of unrealness, and improvement in self-efficacy are related to reductions in PCBD, PTSD, and depression levels in online CBT (Hypothesis 2a). Additionally, we aim to explore whether background characteristics (i.e., gender, age, and educational level, kinship to the deceased, and time since loss) and accident-related stressors (i.e., single vs. multiple loss, witnessing the accident, and status of legal trial) are related to treatment effects (Hypothesis 2b). We have no specific expectations regarding these associations because prior treatment studies in bereaved people showed inconsistent results(24,25,40). However, based on clinical experience, we expect that accident-related stressors are associated with treatment effects, such that multiple loss, witnessing the accident, and on-going legal trial negatively impact treatment effects.

Our third aim is to explore the associations between quality of the therapeutic alliance and drop-out rates and treatment outcomes. We expect that a stronger therapeutic alliance is related to lower dropout rates and better treatment outcomes.

Methods and analysis

Design

A two-arm (online CBT vs. waiting list) multi-centre open label parallel RCT will be conducted. Randomization will take place after the participant is screened for eligibility-based inclusion criteria (described below). A random number generator (www.random.org)

For peer review only - http://bmjopen.bmj.com/site/about/guidelines.xhtml

will be used by a blinded independent researcher, to perform the blocking randomization procedure. An allocation ratio of 1:1 will be applied.

Participants allocated to the online CBT condition receive treatment within one week after allocation. All participants will be asked to fill in questionnaires (described below) at baseline (T1), 12 weeks post-allocation (T2 for the intervention condition and T1a for waitlist controls), and 20 weeks post-allocation (T3 for the intervention condition and T1b for waitlist controls). For participants in the waiting list control group, at the end of the 20-week waiting period after which they will receive online CBT, they will be asked to fill in T2 and T3 12 and 20 weeks after starting treatment, respectively (see Figure 1). A link to online questionnaires will be sent to the participants by a non-blinded member of the research team at each time-point. A waitlist control group (instead of a no treatment control group) is chosen to increase the likelihood of continued study participation by guaranteeing that all participants receive treatment. Furthermore, the inclusion of a waiting list control group allows a treatment versus no treatment comparison, that will provide knowledge about the effects of treatment relative to natural recovery from loss.

In line with prior treatment studies from our research group(40,41), the online treatment is guided by governmentally licensed psychologists, connected with a Dutch informal "traumatic loss network" of therapists specialized in treating emotional distress following traumatic loss. In total six therapists (including authors PB and JdK who are registered clinical psychologists) will guide the participants online; participants will receive feedback from the same therapist each time. The therapists will receive a training, provided by LL, PB, and JdK, on the use of the treatment protocol of this intervention study. In preparation for the training, therapists read all treatment materials and a selection of grief

For peer review only - http://bmjopen.bmj.com/site/about/guidelines.xhtml

treatment literature. Instructions about the use of the online treatment interface will be given by its developers. During a 5-hour face-to-face group meeting the rationale of the online treatment will be explained and research procedures will be discussed. In a 2-hour online video-meeting outstanding questions regarding the treatment and the research project will be answered. Supervision (by telephone or mail) by PB and JdK is possible on request, for instance when therapists encounter difficulties in treatment. Therapists will be contacted by a member of the research team by phone or email biweekly to monitor treatment progress and protocol adherence. Treatment costs will be reimbursed.

==Figure 1 about here==

Participants

This RCT is part of a larger on-going research project (the “TrafVic-project”) examining the psychological impact of, and care after, the death of a loved one due to a traffic accident. We expect to recruit the majority of the participants via a survey that started in December 2018 and included the following question: “In this study we would like to offer psychological help to persons who experience emotional problems. May we approach you with more information about this offer, if your answers to this questionnaire show that you experience emotional problems?” Those who answered ‘yes’ will be sent a letter with information about the intervention, the treatment study, and an informed consent form (see Supplementary Materials). A Dutch website (www.rouwnaverkeersongeval.nl) has been developed so that potential participants can read information about the research and treatment. People who are interested can also sign up for the study via this website. Recruitment for this RCT had not started at the time of submission of this manuscript.

For peer review only - http://bmjopen.bmj.com/site/about/guidelines.xhtml

To be eligible for study participation, the person must 1) be a family member, spouse, or friend of a person who died due to a traffic accident at least one year earlier, 2) be ≥18 years of age, and 3) meet DSM-5 criteria for PCBD and/or PTSD and/or experience clinically relevant depression, based on questionnaire scores (see below for more details). People are excluded when they do not master the Dutch language or have no Internet access.

Sample size

To test our primary hypothesis (Hypothesis 1), a test for each outcome separately (PCBD, PTSD, and depression) will be conducted to assess the effects of online CBT vs. waitlist controls. To find a difference between two groups (online CBT vs. waitlist controls) of at least a medium effect size (f = 0.25; based on prior research(22,28,40)) with a power of 80%, an α of 0.017 (corrected for multiple testing, i.e., 0.05/3, as there are three primary outcome measures (PCBD, PTSD, and depression)), and a strong association (r = .50) between the pre- and post-assessment, a sample size of 23 per condition is sufficient. Taking into account an average dropout rate of 19% (22), a total sample size of 55 (46+9) is required to test Hypothesis 1.

Because our data are nested (repeated measures) (level 1) within individuals (level 2), and possibly within families sharing the same household (level 3), multi-level modeling will be performed to test hypothesis 1. Conducting a power analysis within a multi-level framework is not feasible for various reasons(42). Our power analysis is therefore based on a repeated measures ANOVA.

Intervention

For peer review only - http://bmjopen.bmj.com/site/about/guidelines.xhtml

Online CBT will consist of eight one-on-one sessions, called lessons, offered within a timeframe of 12 weeks. Eight sessions have shown to be sufficient to yield clinically relevant effects in prior research(40). Following Dutch guidelines for grief-specific CBT(42), central components of the treatment are exposure, cognitive restructuring, and behavioral activation. In the first session, psychoeducation is offered, including information about possible emotional reactions to the death of a loved one in a traffic accident and processes that might foster or hamper recovery. A rationale for the CBT interventions is provided.

Then, session 2, 3, and 4 are focused on exposure; the circumstances and story of the loss are presented in detail, and the participant is encouraged to confront stimuli that s/he tends to avoid. Exposure is conducted by imaginary exposure assignments and by writing assignment that have proven to be effective in prior research(30). These writing assignments are focused on writing a detailed narrative of the loss and its circumstances.

The next sessions (5 and 6) focus on identifying and changing negative cognitions that hamper adjustment (i.e., cognitive restructuring); specific attention is paid to cognitions connected with responsibility/guilt and anger that may be experienced following the accidental death(10). Cognitive restructuring assignments are provided to gain an alternative perspective on negative thoughts about the self, life, the future, through 1) psycho-education about common unhelpful thoughts, 2) identifying one's own unhelpful thoughts, and 3) challenging these thoughts. Participants are instructed to undertake these three steps by providing a daily description of i) an emotional moment/event, ii) their thoughts during this event, iii) their feelings (and intensity of these feelings on a scale of 1 through 10), iv) their behavior, v) evaluation of their thoughts), and vi) alternative helpful thoughts.

For peer review only - http://bmjopen.bmj.com/site/about/guidelines.xhtml

In session 7 and 8, participants are encouraged to re-engage in previously valued social, recreational, and occupational activities in order to facilitate the process of adjustment. Behavioral activation assignments are focused on writing about valued activities and making plans to achieve valued goals. Session 8 is also focused on what the participant has learned and how to deal with difficulties in the future.

All information and assignments are presented in an online framework, offered via a secure website. Participants receive online written information that consists of psychoeducation, information about treatment content and structure, and homework assignments. As part of the online treatment, participants also listen to a video-therapist verbally sharing parts of information that are also presented in text. The video-therapists are two members from the traumatic loss network; one male and one female psychotherapist who are middle-aged and specialized in treating bereaved people. At the start of the treatment the video-therapists introduce themselves and the participant is asked to select one of the video-therapists. The information shared by these video-therapists are recorded in video-messages in which they read parts of the texts out loud. Each participant therefore receives the same information from a video-therapist. Direct contact with the video-therapist is not possible.

Participants receive weekly asynchronous written feedback from one online therapist on each assignment that they complete online. As mentioned earlier, six online therapists are trained to guide the participants. The online therapists are instructed to contact the participant twice a week; once to encourage participants to log in and complete assignments and once to provide feedback on assignments. In total, they spend 30 minutes per week on reading assignments and providing feedback. Moreover, participants are encouraged to ask

For peer review only - http://bmjopen.bmj.com/site/about/guidelines.xhtml

a family member or friend to support them during treatment. This support figure is then informed about the treatment through written information in an online framework.

Measures

Primary outcome measures

PCBD will be assessed with the Traumatic Grief Inventory-Self Report (TGI-SR)(43). The TGI-SR consists of 18 items on a 5-point Likert scale ranging from 1 = never through 5 = always. Four items tapping disturbed grief criteria according to the 11th edition of the International Classification of Diseases were added(44). An example of an item is: “I found it difficult to trust others”. The instruction of the original questionnaire was altered from referring to “the death of your loved one” to “the death of your loved one(s) due to a traffic accident”. Psychometric properties of the TGI-SR are adequate(43,45). Participants are considered to meet criteria for DSM-5 PCBD(4) when they score at least 3 (“Sometimes”) on at least 1 criterion B symptom (Item 1, item 2, item 3, and item 14), at least 6 criterion C symptoms (item 4 up to 11, and item 15 up to 18), and the criterion D symptom (item 13).

PTSD will be assessed with the PTSD Checklist for DSM-5 (PCL-5)(46) (Dutch version: (47)). Participants rate how often they were bothered by each symptom (e.g., “In the past month, how much were you bothered by trouble remembering important parts of the accident?”) on 5-point Likert scales (0 = not at all and 4 = extremely). The instruction and the items of the original questionnaire are altered from referring to the “stressful event” to the “the death of your loved one(s) due to a traffic accident”. The PCL-5 has shown to be reliable and valid(46). Participants meet the criteria for DSM-5 PTSD(4) when they score at least 2 ("Moderately") on 1 criterion B item (items 1-5), 1 criterion C item (items 6-7), 2 criterion D items (items 8-14), and 2 criterion E items (items 15-20).

For peer review only - http://bmjopen.bmj.com/site/about/guidelines.xhtml

Depression symptom levels are assessed with the depression subscale of the HADS-D(48). The HADS-D consists of 7 items (e.g., “I still enjoy the thing I used to do”) rated on 4-point scores ranging from 0 (e.g., “Hardly at all”) through 3 (e.g., “Definitely as much”). The Dutch HADS-D is a reliable and valid screening tool for depression(49). A cut-off score of ≥8 is used as indicator for clinically relevant depression(48).

Secondary outcome measures

Negative grief-related cognitions are assessed with 18 items from the Grief Cognitions Questionnaire (GCQ)(14). Participants are asked to rate their agreement with each item (e.g., “Since [–] is dead, I feel less worthy”) on 6-point scales varying from 0 = disagree strongly through 5 = agree strongly. The psychometric properties have been positively evaluated in prior research(14).

Avoidance is measured with the Depressive and Anxious Avoidance in Prolonged Grief Questionnaire (DAAPGQ)(50). The depressive avoidance subscale consists of 5 items (e.g. ‘Since [–] is dead, I do much less of the things that I used to enjoy.’) and the anxious avoidance subscale consists of 4 items (e.g., ‘I avoid to dwell on painful thoughts and memories connected to his/her death.’). Participants answer each item on an 8-point scale with 0 = not at all true for me, and 7 = completely true for me. The DAAPGQ has adequate psychometric properties(50).

State anger is assessed with the 15-item state anger subscale of the State-Trait Anger Expression Inventory-2 (STAXI-2)(51) (Dutch version:(52)). Participants are asked to rate on 4-point Likert scales (1 = not at all and 4 = extremely) how angry they feel right now (e.g., “I feel annoyed”). The STAXI-2 is a valid and reliable measure to assess state anger(52).

For peer review only - http://bmjopen.bmj.com/site/about/guidelines.xhtml

A sense of unrealness is measured with the 5-item Experienced Unrealness Scale(17). Participants are asked to rate their agreement with each item (e.g., "I still can hardly imagine that [–] will never be here again") on 8-point scales (0 = not at all true for me 7 = completely true for me). This instrument demonstrated adequate psychometric properties(17).

Self-efficacy is assessed with the General Self-Efficacy Scale (GSES)(53). The GSES is a 10-item measure. Participants are asked to rate their agreement with each item (e.g., "I can solve most problems if I invest the necessary effort.") on a 4-point scale (1 = completely not true, 4 = completely true). The GSES has shown excellent reliability and validity(53).

Quality of the therapeutic alliance is measured with the 12-item Work Alliance Inventory-Short Form, Client Version and Therapist Version after session 4 (WAI-SF)(54) (Dutch version:(55). The WAI-SF consists of 12 items (e.g., Client version: "We agree on what is important for me to work on", Therapist Version: "We are working towards mutually agreed upon goals.") that are rated on 5-point scales (1 = never and 5 = always). Higher total scores indicate a higher quality of the therapeutic alliance as perceived by the participant and therapist. The WAI-SF is a reliable and valid assessment tool(56).

Other measures

Background characteristics (i.e., gender, age, and educational level, kinship to the deceased, and time since loss) and accident-related stressors (i.e., single vs. multiple loss, witnessed the accident, and status of legal trial) will be assessed with single items.

Participants are allowed to receive other forms of psychosocial, instrumental or legal support during participation in the trial. Using a single question we will assess whether the participants received other forms of psychosocial professional support. The following

For peer review only - http://bmjopen.bmj.com/site/about/guidelines.xhtml

question will be used: “During the past 12 weeks/8 weeks (for T2 and T3, respectively) did you receive additional psychological professional support from a psychologist, therapist or psychiatrist other than the (online) therapist from the TrafVic-study?” We will also include two dichotomous items (yes/no) at T1 to assess psychological support received prior to participation in the study, namely: “Did you ever receive support from a psychologist, therapist or psychiatrist, for your own emotional/mental problems, prior to the loss of your loved one due to a traffic accident?” and “Did you ever receive support from a psychologist, therapist or psychiatrist, for your own emotional/mental problems, related to the loss of your loved one due to a traffic accident?”

Statistical analyses

To examine the differences in reductions of symptom levels of PCBD, PTSD, and depression from pre- to post-treatment/waiting period between the conditions (online CBT vs. waitlist), three independent multi-level models will be built (Hypothesis 1). Symptom levels of PCBD, PTSD, and depression will consecutively be included as dependent variables and condition (online CBT vs. waitlist controls), time, and time x condition (interaction term) as predictor variables, taking into account that repeated observations (level 1) are nested within individuals (level 2), and within households (level 3; if applicable). Additionally, relevant background, loss-related variables, and use of co-interventions (yes/no) during participation in our study, will be included in the analysis as covariates. Deviance tests will be used to examine whether inclusion of these covariates improves model fit(57). Data of all participants entering the study will be included in all analyses (i.e., intention-to-treat analysis). Furthermore, percentages of people meeting diagnostic criteria for PCBD, PTSD,

For peer review only - http://bmjopen.bmj.com/site/about/guidelines.xhtml

and clinically relevant depression will be calculated for each measurement occasion and percentages of people reporting reliable change scores for each outcome measure, using a formula from Jacobson and Truax(58, p. 14), will be reported.

To examine to what extent symptom improvement after treatment is related to improvement in possible correlates of change, residual gain scores will be calculated for all outcome measures (i.e., PCBD, PTSD, and depression) and possible correlates of change (i.e., negative cognitions, avoidance behaviors, state anger, a sense of unrealness, and self-efficacy). Following previous research (cf. (59)), residual gain scores will be calculated by subtracting the standardized combined pre-treatment scores of both conditions (T1 data from immediate treatment condition and T1b data from waitlist condition) multiplied by the correlation coefficient between standardized combined pre-treatment scores and standardized post-treatment (or follow-up) scores from standardized post-treatment (or follow up) scores. To test hypothesis 2a, multiple regression analyses will be conducted to examine the associations between residual gain scores of PCBD, PTSD, or depression and residual gain scores of negative cognitions, avoidance behaviors, state anger, a sense of unrealness, and self-efficacy.

To achieve research aim 2b, multiple regression analyses will be used to examine to what extent residual gain scores of PCBD, PTSD, and depression varies as function of a) background characteristics, including gender (male/female), age (in years), and educational level (low/high), kinship to the deceased (child/spouse vs other), and time since loss (in years) and b) accident-related stressors, including number of losses (single vs multiple), witnessing the accident (yes/no), and status of legal trial (not applicable/on-

For peer review only - http://bmjopen.bmj.com/site/about/guidelines.xhtml

going/completed). Condition (intervention vs. waitlist controls) will be added as a covariate to fulfill research aim 2a and 2b.

To achieve the third research aim, a) differences in therapeutic alliance scores will be assessed between people who completed and dropped out of treatment and b) multiple regression analyses will be used to examine to what extent symptom improvement in PCBD, PTSD, and depression is related to therapeutic alliance (from both participant and therapist perspectives).

Ethics and dissemination

The initial plan for this study was to conduct a three-arm (face-to-face CBT, online CBT, and waiting list) RCT to examine the effectiveness of face-to-face CBT (vs. waitlist controls) *and* online CBT (vs. waitlist controls). This study has been approved by the Medical Ethics Review Board of the University Medical Center Groningen (METc UMCG: ID number: M20.252121). Due to the COVID-19 outbreak we had to change our study protocol, because face-to-face contact with a therapist was not possible because of social distancing measures. Instead of comparing the effects of online and face-to-face CBT with waitlist controls, we changed the design of the study by comparing the effects of online CBT vs. waitlist controls before enrolment of participants took place. This amendment to our study has been approved by the same ethics committee.

The study will be conducted according to the principles of the Declaration of Helsinki (8th version, 2013) and in accordance with the Medical Research Involving Human Subjects Act. Collected data will be handled confidentially, according to the EU General Data Protection Regulation and the Dutch Act on Implementation of the General Data Protection

For peer review only - http://bmjopen.bmj.com/site/about/guidelines.xhtml

Regulation. Unidentifiable data from this trial will be stored in data repositories from the University of Groningen and Utrecht University.

Findings of this RCT will be disseminated among participants by means of a newsletter. If shown to be effective, the online framework will be made publicly accessible, so that it can benefit other bereaved people. Findings will also be disseminated among lay people by uploading the newsletters on our website (www.rouwnaverkeersongeval.nl) and through media performances. Our findings will be presented to our collaborators, including non-governmental organizations and (peer-)support organizations for bereaved people. Treatment materials will also be made available upon request. Lastly, colleagues will be informed about our findings during presentations at (inter)national conferences and publications in scientific journals.

Patient and public involvement

At the start of this project an advisory committee was established. This committee includes someone who lost a significant other after a traffic accident, a lawyer with expertise in supporting bereaved people after traffic accidents, and representatives of Victim Support the Netherlands and Fund Victim Support. This committee was involved in the development of the research questions, outcomes measures, and design of the study by reading and commenting on drafts of our research proposal and study-protocol. This committee pilot tested the questionnaires and was involved in the development of recruitment materials, recruitment strategies, and information materials for participants by reading, revising, and approving the drafts. This committee helps the research team in recruiting participants by sharing information about this study in their own professional network. The advisory committee is not involved in conducting the study or development of treatment materials.

For peer review only - http://bmjopen.bmj.com/site/about/guidelines.xhtml

The committee will support the research team when disseminating the study findings among relevant audiences by help writing and reviewing newsletters and press releases.

Discussion

The relatively few RCTs among general bereaved people with elevated grief levels indicate that grief-specific CBT-based interventions yield the largest effects on post-loss mental health compared with a waiting list(21–24). RCTs evaluating face-to-face or online treatment effects for people with elevated mental health complaints after confrontation with sudden/violent losses are lacking, with the exception of two studies that compared face-to-face EMDR plus CBT against waitlist controls(40,59). Given that traumatically bereaved people are at risk for PCBD and comorbid PTSD and depression(8), it seems particularly relevant to develop evidence-based interventions for this population.

This will be the first RCT to examine the effectiveness of online CBT in a sample exclusively comprised of people who experienced a traumatic death. We are not able to test whether the online CBT has equal effects as face-to-face CBT. Nonetheless, the findings are expected to yield important insights in the effects of online CBT. In this RCT, the online treatment is designed to be as similar as possible to face-face CBT in terms of treatment content, treatment duration, and experience and training of therapists. When we find effect sizes for online CBT that are similar to effect sizes found in earlier studies for face-to-face CBT, delivering CBT online can be considered as supplement to face-to-face treatment, in particular when barriers to face-to-face treatments, such as waiting lists and travel expenses, are experienced.

We will also examine potential correlates of change. These analyses, examining the associations between reductions in symptoms levels and among others negative cognitions

For peer review only - http://bmjopen.bmj.com/site/about/guidelines.xhtml

and avoidance behaviors, will provide insights in potential underlying therapeutic processes to foster recovery from traumatic loss. These insights are deemed important to design treatments that more effectively target these correlates of change. We also expect to improve our knowledge on for whom (e.g., women or people who are more remotely bereaved) grief-specific CBT works best. Findings on these potential correlates of change are necessary to improve treatments given that a maximum of 42% of bereaved people report clinically relevant reductions in grief levels after treatment(21).

Lastly, the role of therapeutic alliance on therapy outcomes will be explored. Prior research in bereaved people has shown that greater therapeutic alliance, from the perspective of the client, at week 4 of a face-to-face grief-specific treatment, was related to greater reductions in grief levels. This therapeutic alliance-grief relationship was not significant for a non-grief specific treatment(60). Our exploration of this association, from the perspective of client *and* therapist, may for the first time shed light on therapeutic processes in online CBT for traumatic grief.

An anticipated limitation of our RCT is the self-selected sample. It is possible that people who are more open towards innovative technology in general(61) and who received support prior to the loss(32) are more likely to sign up for this study, limiting the generalizability of findings emerging from this study. Due to the absence of an active control group (e.g., face-to-face CBT) we are not able to test the effects of online CBT compared with an alternative treatment. Furthermore, we will use self-report measures instead of diagnostic interviews, which may increase the risk of overestimating symptom levels(62). In addition, participants might experience difficulties with completing the mid-treatment assessment of the therapeutic relationship because the video-therapist that provides

For peer review only - http://bmjopen.bmj.com/site/about/guidelines.xhtml

information through recorded video messages (interaction between video-therapist and participant is not possible) might be a different person than the online therapist who provides personal written feedback twice a week. Although the instructions of the therapeutic alliance measure explicitly refer to the interaction with the online therapist (not the video-therapist), this might still be confusing for some participants. Another potential limitation of this trial relates to the fact that the operationalization and assessment of grief as a disorder is still under debate(63–65). For instance, PCBD, included as "condition for further study" in the DSM-5, is likely to be changed in a revision of the DSM. To maximize diagnostic compatibility, we added four items, corresponding to ICD-11 PGD criteria, to the TGI-SR, enabling operationalizion of our primary outcome measure in terms of diagnoses of pathological grief according to both the DSM-5 and the ICD-11.

To conclude, this RCT will provide new insights in effectiveness of online CBT for people who experience clinically relevant distress after bereavement due to a traffic accident, as well as in potential correlates of therapeutic change. As trials to date have primarily focused on effects of face-to-face treatment for non-traumatically bereaved people, our findings are expected to provide a valuable addition to the knowledge base on treating severely distressed bereaved people.

For peer review only

References

1. World Health Organization. The top 10 causes of death [Internet]. 2019 [geciteerd 14 oktober 2019]. Beschikbaar op: https://www.who.int/news-room/fact-sheets/detail/the-top-10-causes-of-death

2. Lundorff M, Holmgren H, Zachariae R, Farver-Vestergaard I, O'Connor M. Prevalence of prolonged grief disorder in adult bereavement: A systematic review and meta-analysis. J Affect Disord. 2017;212:138–49.

3. Onrust SA, Cuijpers P. Mood and anxiety disorders in widowhood: a systematic review. Aging Ment Health. 2006;10(4):327–34.

4. American Psychiatric Association. Diagnostic and statistical manual of mental disorders (5th ed.). Arlington, VA: American Psychiatric Publishing; 2013.

5. Boelen PA, van de Schoot R, van den Hout MA, de Keijser J, van den Bout J. Prolonged Grief Disorder, depression, and posttraumatic stress disorder are distinguishable syndromes. J Affect Disord. 2010;125(1–3):374–8.

6. Lenferink LIM, Nickerson A, de Keijser J, Smid GE, Boelen PA. Trajectories of grief, depression, and posttraumatic stress in disaster-bereaved people. Depress Anxiety. 2018;

7. Malgaroli M, Maccallum F, Bonanno GA. Symptoms of persistent complex bereavement disorder, depression, and PTSD in a conjugally bereaved sample: a network analysis. Psychol Med. 2018;48(14):2439–48.

For peer review only

For peer review only - http://bmjopen.bmj.com/site/about/guidelines.xhtml

8. Heeke C, Kampisiou C, Niemeyer H, Knaevelsrud C. A systematic review and meta-analysis of correlates of prolonged grief disorder in adults exposed to violent loss. Eur J Psychotraumatology [Internet]. 2019 [geciteerd 14 oktober 2019];10(1). Beschikbaar op: https://www.ncbi.nlm.nih.gov/pmc/articles/PMC6442112/

9. Kristensen P, Weisæth L, Heir T. Bereavement and mental health after sudden and violent losses: a review. Psychiatry. 2012;75(1):76–97.

10. Eisma, M.C., de Keijser, J. Nabestaanden van verkeersongevallen. In: de Keijser, J., Boelen, P.A., Smid, G.E., redacteuren. Handboek traumatische rouw. Amsterdam: Uitgeverij Boom; 2018. p. 104–16.

11. Boelen PA, de Keijser J, Smid G. Cognitive–behavioral variables mediate the impact of violent loss on post-loss psychopathology. Psychol Trauma Theory Res Pract Policy. 2015;7(4):382–90.

12. Boelen PA, van den Hout MA, van den Bout J. A Cognitive-Behavioral Conceptualization of Complicated Grief. Clin Psychol Sci Pract. 2006;13(2):109–28.

13. Janoff-Bulman R. Assumptive worlds and the stress of traumatic events: Applications of the schema construct. Soc Cogn. 1989;7(2):113–36.

14. Boelen PA, Lensvelt-Mulders GJLM. Psychometric Properties of the Grief Cognitions Questionnaire (GCQ). J Psychopathol Behav Assess. 2005;27(4):291–303.

15. Foa EB, Riggs DS, Massie ED, Yarczower M. The impact of fear activation and anger on the efficacy of exposure treatment for posttraumatic stress disorder. Behav Ther. 1995;26(3):487–99.

For peer review only - http://bmjopen.bmj.com/site/about/guidelines.xhtml

For peer review only

16. Tehrani, N. Road victim trauma: an investigation of the impact on the injured and bereaved. Couns Psychol Q. 2004;17(4):361–73.

17. Boelen PA. A Sense of 'unrealness' about the death of a loved-one: An exploratory study of its role in emotional complications among bereaved individuals. Appl Cogn Psychol. 2010;24(2):238–51.

18. Boelen PA. "It feels as if she might return one day": A sense of unrealness as a predictor of bereavement-related emotional distress / "Tengo la sensación de que ella puede volver algún día": la sensación de irrealidad como un predictor del sufrimiento emocional relacionado con la pérdida. Estud Psicol. 2017;38(3):734–51.

19. Benight CC. Social cognitive theory of posttraumatic recovery: the role of perceived self-efficacy. Behav Res Ther. 2004;42(10):1129–48.

20. Smid GE, Kleber RJ, Rie SM de la, Bos JBA, Gersons BPR, Boelen PA. Brief Eclectic Psychotherapy for Traumatic Grief (BEP-TG): toward integrated treatment of symptoms related to traumatic loss. Eur J Psychotraumatology. 2015;6(1):27324.

21. Boelen PA, Smid GE. Disturbed grief: prolonged grief disorder and persistent complex bereavement disorder. BMJ. 2017;357:j2016.

22. Currier JM, Holland JM, Neimeyer RA. Do CBT-Based Interventions Alleviate Distress Following Bereavement? A Review of the Current Evidence. International Journal of Cognitive Therapy. 2010;3:77–93.

23. Doering BK, Eisma MC. Treatment for complicated grief: state of the science and ways forward. Curr Opin Psychiatry. 2016;29(5):286–91.

For peer review only - http://bmjopen.bmj.com/site/about/guidelines.xhtml

24. Johannsen M, Damholdt MF, Zachariae R, Lundorff M, Farver-Vestergaard I, O'Connor M. Psychological interventions for grief in adults: A systematic review and meta-analysis of randomized controlled trials. J Affect Disord. 2019;253:69–86.

25. Boelen PA, Keijser J de, Hout MA van den, Bout J van den. Factors associated with outcome of cognitive–behavioural therapy for complicated grief: A preliminary study. Clin Psychol Psychother. 2011;18(4):284–91.

26. Bryant RA, Kenny L, Joscelyne A, Rawson N, Maccallum F, Cahill C, e.a. Predictors of treatment response for cognitive behaviour therapy for prolonged grief disorder. Eur J Psychotraumatology [Internet]. 2019 [geciteerd 13 oktober 2019];8(6). Beschikbaar op: https://www.ncbi.nlm.nih.gov/pmc/articles/PMC6383610/

27. Linde K, Treml J, Steinig J, Nagl M, Kersting A. Grief interventions for people bereaved by suicide: A systematic review. PloS One. 2017;12(6):e0179496.

28. Eisma MC, Boelen PA, van den Bout J, Stroebe W, Schut HAW, Lancee J, e.a. Internet-Based Exposure and Behavioral Activation for Complicated Grief and Rumination: A Randomized Controlled Trial. Behav Ther. 2015;46(6):729–48.

29. Kersting A, Dölemeyer R, Steinig J, Walter F, Kroker K, Baust K, e.a. Brief Internet-based intervention reduces posttraumatic stress and prolonged grief in parents after the loss of a child during pregnancy: a randomized controlled trial. Psychother Psychosom. 2013;82(6):372–81.

30. Wagner B, Knaevelsrud C, Maercker A. Internet-based cognitive-behavioral therapy for complicated grief: a randomized controlled trial. Death Stud. 2006;30(5):429–53.

For peer review only - http://bmjopen.bmj.com/site/about/guidelines.xhtml

31. Baumeister H, Reichler L, Munzinger M, Lin J. The impact of guidance on Internet-based mental health interventions — A systematic review. Internet Interv. 2014;1(4):205–15.

32. Lichtenthal WG, Corner GW, Sweeney CR, Wiener L, Roberts KE, Baser RE, e.a. Mental Health Services for Parents Who Lost a Child to Cancer: If We Build Them, Will They Come? J Clin Oncol Off J Am Soc Clin Oncol. 2015;33(20):2246–53.

33. Andersson G, Titov N. Advantages and limitations of Internet-based interventions for common mental disorders. World Psychiatry. 2014;13(1):4–11.

34. Eisma MC, Boelen PA, Lenferink LIM. Prolonged grief disorder following the Coronavirus (COVID-19) pandemic. Psychiatry Res. 2020;288:113031.

35. Melville KM, Casey LM, Kavanagh DJ. Dropout from Internet-based treatment for psychological disorders. Br J Clin Psychol. 2010;49(Pt 4):455–71.

36. Pihlaja S, Stenberg J-H, Joutsenniemi K, Mehik H, Ritola V, Joffe G. Therapeutic alliance in guided internet therapy programs for depression and anxiety disorders - A systematic review. Internet Interv. 2018;11:1–10.

37. Bordin ES. Theory and research on the therapeutic working alliance: New directions. In: The working alliance: Theory, research, and practice. Oxford, England: John Wiley & Sons; 1994. p. 13–37. (Wiley series on personality processes).

38. Cook JE, Doyle C. Working alliance in online therapy as compared to face-to-face therapy: preliminary results. Cyberpsychology Behav Impact Internet Multimed Virtual Real Behav Soc. 2002;5(2):95–105.

For peer review only - http://bmjopen.bmj.com/site/about/guidelines.xhtml

39. Sucala M, Schnur JB, Constantino MJ, Miller SJ, Brackman EH, Montgomery GH. The therapeutic relationship in e-therapy for mental health: A systematic review. J Med Internet Res. 2012;14(4):e110.

40. van Denderen M, de Keijser J, Stewart R, Boelen PA. Treating complicated grief and posttraumatic stress in homicidally bereaved individuals: A randomized controlled trial. Clin Psychol Psychother. 2018;

41. Lenferink LIM, de Keijser J, Wessel I, Boelen PA. Cognitive behavioural therapy and mindfulness for relatives of missing persons: A pilot study. Pilot Feasibility Stud. 2019;5(1):93.

42. Boelen PA, Bout J van den. Protocollaire behandeling van persisterende complexe rouwstoornis. In: Keijsers, G., Minnen, A. van, Verbraak, M., Hoogduin, K., Emmelkamp, P., redacteuren. Protocollaire behandelingen voor volwassenen met psychische klachten. 1ste dr. Amsterdam: Boom; 2017.

43. Boelen PA, Smid GE. The Traumatic Grief Inventory Self-Report Version (TGI-SR): Introduction and Preliminary Psychometric Evaluation. J Loss Trauma. 2017;22(3):196–212.

44. World Health Organization. ICD-11. Prolonged Grief Disorder Criteria [Internet]. 2018 [geciteerd 14 oktober 2019]. Beschikbaar op: https://icd.who.int/browse11/l-m/en#/http://id.who.int/icd/entity/1183832314

For peer review only

45. Boelen PA, Djelantik AAAMJ, de Keijser J, Lenferink LIM, Smid GE. Further validation of the Traumatic Grief Inventory-Self Report (TGI-SR): A measure of persistent complex bereavement disorder and prolonged grief disorder. Death Stud. 2019;43(6):351–64.

46. Blevins CA, Weathers FW, Davis MT, Witte TK, Domino JL. The Posttraumatic Stress Disorder Checklist for DSM-5 (PCL-5): Development and Initial Psychometric Evaluation. J Trauma Stress. 2015;28(6):489–98.

47. Boeschoten, M.A., Bakker, A., Jongedijk, R.A., Olff, M. PTSS checklist voor de DSM-5 (PCL-5). Diemen: Arq Nationaal Psychotrauma Centrum; 2014.

48. Zigmond AS, Snaith RP. The hospital anxiety and depression scale. Acta Psychiatr Scand. 1983;67(6):361–70.

49. Spinhoven PH, Ormel J, Sloekers PPA, Kempen GIJM. A validation study of the Hospital Anxiety and Depression scale (HADS) in different groups of Dutch subjects. Psychol Med. 1997;27(2):363–70.

50. Boelen PA, van den Bout J. Anxious and depressive avoidance and symptoms of prolonged grief, depression, and post-traumatic stress disorder. Psychol Belg. 2010;50(1–2):49–67.

51. Spielberger, C.D. State-Trait Anger Expression Inventory-2. Psychological Assessment Resources; 1999.

52. Lievaart M, Franken IHA, Hovens JE. Anger Assessment in Clinical and Nonclinical Populations: Further Validation of the State-Trait Anger Expression Inventory-2. J Clin Psychol. 2016;72(3):263–78.

For peer review only - http://bmjopen.bmj.com/site/about/guidelines.xhtml

53. Schwarzer, R., Jerusalem, M. Generalized self-efficacy scale. In: Weinman, J., Wright, S., Johnston, M., redacteuren. Measures in health psychology: A user's portfolio Causal and control beliefs. Windsor, UK: Nfer-Nelson; 1995. p. 35–7.

54. Horvath AO, Greenberg LS. Development and validation of the Working Alliance Inventory. J Couns Psychol. 1989;36(2):223–33.

55. Vertommen, H., Vervaeke, G. Nederlandstalige experimentele vertaling van de Working Alliance Inventory van Horvath en Greenberg. Leuven: KU; 1990.

56. Munder T, Wilmers F, Leonhart R, Linster HW, Barth J. Working Alliance Inventory-Short Revised (WAI-SR): psychometric properties in outpatients and inpatients. Clin Psychol Psychother. 2010;17(3):231–9.

57. Snijders, T.A.B., Bosker, R.J. Deviance tests. In: Snijders, T.A.B., Bosker, R.J., redacteuren. Multilevel analysis. London: SAGE Publications Ltd; 2012. p. 97–8.

58. Jacobson NS, Truax P. Clinical significance: A statistical approach to defining meaningful change in psychotherapy research. J Consult Clin Psychol. 1991;59(1):12–9.

59. Lenferink LIM, de Keijser J, Smid GE, Boelen PA. Cognitive therapy and EMDR for reducing psychopathology in bereaved people after the MH17 plane crash: Findings from a randomized controlled trial. Traumatology. 2020;

60. Glickman K, Shear, K.M., Wall MM. Therapeutic Alliance and Outcome in Complicated Grief Treatment. Int J Cogn Ther. 2018;11(2):222–33.

61. Arjadi R, Nauta MH, Bockting CLH. Acceptability of internet-based interventions for depression in Indonesia. Internet Interv. 2018;13:8–15.

For peer review only - http://bmjopen.bmj.com/site/about/guidelines.xhtml

62. Lim GY, Tam WW, Lu Y, Ho CS, Zhang MW, Ho RC. Prevalence of Depression in the Community from 30 Countries between 1994 and 2014. Sci Rep. 2018;8(1):2861.

63. Lenferink, L.I.M., Boelen, P.A., Smid, G.E., Paap, M.C.S. The importance of harmonising diagnostic criteria sets for pathological grief. British Journal of Psychiatry. in press;

64. Maciejewski PK, Prigerson HG. Prolonged, but not complicated, grief is a mental disorder. Br J Psychiatry. 2017;211(4):189–91.

65. Wakefield JC. Should prolonged grief be reclassified as a mental disorder in DSM-5?: reconsidering the empirical and conceptual arguments for complicated grief disorder. J Nerv Ment Dis. 2012;200(6):499–511.

For peer review only

Authors' contributions

JdK is principal investigator. LL is executive researcher. JdK, PB, ME, and GS are grant holders. LL developed the study design and wrote the ethics proposal and drafts of the manuscript. JdK, ME, GS, and PB read, revised, and approved the drafts of the study design, ethics proposal, and the manuscript.

Funding statement

Fund Victim Support subsidized this work (grant number: not applicable).

Competing Interests Statement

All authors declare to have no competing interests.

For peer review only - http://bmjopen.bmj.com/site/about/guidelines.xhtml

Figure caption/legend

Figure 1. Design of RCT

Note. CBT = cognitive behavioral therapy

For peer review only

For peer review only - http://bmjopen.bmj.com/site/about/guidelines.xhtml

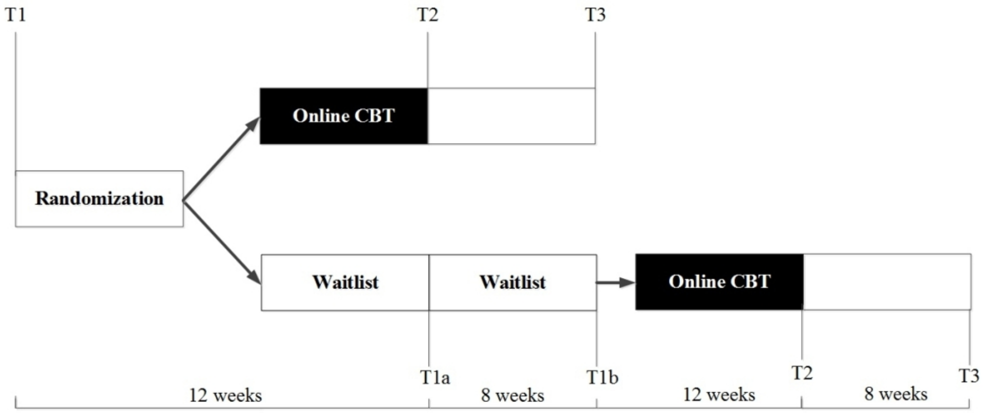

Figure 1. Design of RCT

356x148mm (96 x 96 DPI)

Supplementary File

Information for participants

Subject: Participation in study on the treatment of grief after a traffic accident

Dear sir/madam,

We ask you to take part in a scientific study. This participation is voluntary. In order to take part we do need your permission. You receive this information because you filled in a questionnaire some time ago, mapping the emotional consequences of the death of a loved one due to a traffic accident. You have indicated that you may be interested in taking part in a follow-up study exploring the effects of a treatment to learn to cope better with the death of your loved one.

Before you decide whether you want to take part in this study, you receive an explanation of what the study entails. Read this information carefully and ask the researcher to explain if you have questions.

1. **General Information**

This study is conducted by the University of Groningen, Utrecht University and Stichting Centrum '45. The study is financed by the Fonds Slachtofferhulp (Victim Support Fund). The study consists of participation in an online psychological treatment. The treatment is provided by therapists in various practices across the Netherlands. The medical-ethical review committee of the Universitair Medisch Centrum Groningen (Academic Medical Centre Groningen) has approved this study.

2. **Purpose of the study**

Those left behind after a traffic accident often indicate that the help provided does not sufficiently connect to their experiences. Therefore a treatment has been developed that is specifically intended for the partner, relatives and friends of someone who died due to a traffic accident. You can discuss with the therapist how you are coping with the loss and what the consequences of this loss are in your life. The aim of the treatment is to cope better with the loss. The treatment is part of a scientific study. The purpose of the study is to explore whether treatment leads to a reduction in emotional problems for those who lost someone due to a traffic accident.

3. **Background of the study**

During the treatment cognitive behavioural therapy will be used. Previous research has shown that cognitive behavioural therapy is the most effective treatment for reducing emotional problems after the loss of a loved one due to a natural cause (for instance illness). Cognitive behavioural therapy is mainly applied during individual sessions with a therapist (face-to-face treatment). Research has shown that cognitive behavioural therapy, offered via the internet (online treatment), also seems to be suitable for reducing problems after a natural death. More research is needed to find out whether this online version of cognitive behavioural therapy is also suitable for those who lost someone due to a traffic accident. The purpose of this study is to find out whether online cognitive behavioural therapy is accompanied by a reduction of emotional problems after the death of a loved one due to a traffic accident.

The online treatment is provided individually and consists of eight modules which you go through in twelve weeks. You will then have online contact with a therapist who will guide you during the treatment. In the Netherlands, a network of therapists has been trained in the online treatment of people

For peer review only

who lost someone due to a traffic accident. The therapists work at several treatment centres. The online treatment is offered by Therapieland. Therapieland is a provider of psychological care via the internet.

4. What participation entails

If you take part, this will take at least 20 weeks in total for you.

Screening

First we will determine whether you can take part. You will be asked to fill in a questionnaire. The questionnaire contains questions about emotional problems you may experience in response to the passing away of your loved one due to a traffic accident. Also, questions are asked about previous psychological help you may have received. The questionnaire is used to get a picture of the degree to which you experience emotional problems.

If your completed questionnaire shows that you experience relatively few emotional problems, you cannot take part in the study. You also cannot take part in the study if you have no access to the internet. If your answers show that the treatment offered is not suitable for you, an alternative treatment will be looked for in consultation with you.

It is possible that you filled out a questionnaire on this topic before. As problems can change over time, we ask you to fill in a questionnaire once more. In this way we get a picture of the problems you are experiencing at the moment.

Treatment

In order to be able to determine the effect of the treatment, participants are assigned to one of two groups. The first group will start with the online treatment as soon as possible after registration. The second group will start with the online treatment after a waiting period of 20 weeks. By adding a waitlist group it can be determined that a reduction of problems is actually the result of the treatment, and not of the passage of time. Which group you are assigned to will be determined by drawing lots. You and we do not have any influence on the draw. We will let you know when your treatment starts.

Measurements

Before the treatment can start, you will be asked to fill in a questionnaire. This questionnaire will focus on the problems you experience. We map these in order to be able to determine whether the treatment might help you.

The therapist who will guide your online treatment will be informed of the results of this questionnaire beforehand. In order to determine to what extent the treatment helps you, we ask you to fill in a questionnaire not only beforehand, but also once during the treatment, after the last treatment session, and 8 weeks after the treatment. People assigned to the waiting group will be asked to fill in an additional questionnaire after 12 weeks and 20 weeks of waiting. In this way it can be determined whether the treatment has been effective and what the short and long term effects of the treatment are. The filling in will take approximately 10 to 30 minutes per measurement.

5. What is expected from you

In order to ensure that the study runs smoothly it is important that you adhere to the following agreements. These agreements are that you:

- contact the researcher in case of problems (the contact details are listed at the end of the information letter);
- keep all appointments with your therapist;
- fill in the questionnaires before and after the treatment.

For peer review only

Besides, it is important that you contact the researcher if:

- your contact information changes
- you no longer wish to take part in the study

6. Possible negative effects

During the treatment you will actively engage with your thoughts and feelings about the loss. It is possible that feelings such as grief or loss or fatigue may temporarily increase in intensity. Filling in the questionnaire can also evoke emotional responses. When your problems increase to a great extent, you can contact the researcher via the contact details listed at the end of this letter.

7. Possible advantages and disadvantages

It is important that you carefully weigh the possible advantages and disadvantages before you decide to take part. The treatment may reduce your emotional problems, but this is not certain. Your participation will contribute to more knowledge about the treatment of emotional problems after the death of a loved one due to a traffic accident.

Disadvantages of taking part in the study may be:

- possible worsening of problems due to taking part in the treatment;
- possible worsening of problems due to filling in the questionnaire.

Participation in the study also means that:

- the study will cost you time;
- you will have to adhere to certain agreements.

These issues have all been described in section 4, 5 and 6 above.

8. If you do not wish to participate or if you wish to stop participating in the study

You decide whether or not to take part in the study. Participation is voluntary. If you do take part, you can always change your mind and stop participating after all, during the study as well. You do not have to give a reason for stopping. You do have to report this to the researcher immediately. The data collected up to that point will be used for the study.

If there is new information about the study that is important to you, the researcher will let you know this. We will then ask you if you continue to participate.

9. End of the study

Your participation in the study will end when: you will have filled in the questionnaire 8 weeks after the end of the treatment; you make the choice to stop participating; the researcher considers it better for you to stop participating; the government or the reviewing medical-ethical committee decide to end the study. The entire study will end when all participants are done. After processing all the data, the researcher will inform you of the most important outcomes of the study by means of a newsletter. You can indicate whether you wish to receive this newsletter at the end of the questionnaire.

For peer review only

10. Use and storage of your data

For this study your personal data will be collected, used and stored. This concerns data such as your name, address, date of birth and data related to your health. The collection, use and storage of your data is necessary to be able to answer the questions asked in this study and to publish the results. We ask your permission for the use of your data.

Confidentiality of your data

In order to protect your privacy, your data will get a code. Your name and other data which can identify you directly will be left out from this. Data can only be traced to you with the key to the code. The key to the code will remain safely stored at the local research institution. The data sent to eventual other parties involved only contain the code, but not your name or other data with which you can be identified. In reports and publications on the study the data cannot be traced to you either.

Access to your data for checks

Some people may get access to all your data at the research location, including the data without code. This is necessary to be able to check whether the study has been done properly and reliably. People who will gain access to you data for checking purposes will be the researchers Lonneke Lenferink, a research assistant and Jos de Keijser, the committee monitoring the safety of the study and international supervising authorities. They will keep your data secret. We ask you to give your permission for this access.

Data retention period

Your data must be retained at the research location for 15 years. They are stored in order to be able to make new provisions related to this study in the course of this study.

Withdrawal of permission

You can always withdraw your permission for the use of your personal data again. The research data collected up to the moment you withdraw permission will still be used in the study.

Further information about your rights regarding data processing

For general information about you rights you can contact the person responsible for processing your personal data. For this study this is Lonneke Lenferink (University of Groningen). In case of questions or complaints regarding the processing of your personal data we recommend that you contact her.

11. Insurance for test subjects

An insurance has been taken out for everyone taking part in the study. The insurance covers damage from the study. You can report damage to the researchers.

12. Informing family doctor/GP

We do not share information about your participation in the study with your family doctor/GP.

13. Compensation for participation

For peer review only

Participation in the online treatment is free of charge.

14. Do you have any questions?

In case of general questions regarding the study you can contact the researcher assistant. If you have complaints about the study, you can discuss this with the researchers or with your treating therapist. If you prefer not to do this, you can contact the University of Groningen.

15. Signing the consent form

Below you find a 'Declaration of consent for participation in research into the treatment of grief after a traffic accident', on which you can indicate whether you wish to take part in the study. With your permission you indicate that you have understood the information and that you agree to participate in the study.

After you have filled in this declaration, you can start filling in the questionnaire. Approximately within four weeks after filling in the questionnaire we will let you know by email or by phone whether you qualify for the treatment. When you qualify for treatment, you will receive the outcome of the draw which indicates whether you have been assigned to a group that can start with the online treatment as soon as possible, or whether you have been assigned to the group that needs to wait for 20 weeks before the start of the online treatment.

If you have any further questions, you can contact us via the details below.

Kind regards,

[name research assistant]

Research assistant University of Groningen

Lonneke Lenferink

Post-doctoral researcher University of Groningen and Utrecht University

Contact Details

General questions about the study: [name research assistant], info@rouwnaverkeersongeval.nl

Questions regarding the protection of your data, your rights or complaints: please contact Lonneke Lenferink, l.i.m.lenferink@rug.nl.

For peer review only

Consent Form
for participation in research on treatment of grief after a traffic accident

- I have read the information letter. I also had the opportunity to ask questions. My questions have been answered adequately. I had enough time to decide whether to participate or not.
- I know that participation is voluntary. I also know that I can decide at any moment not to participate after all or to stop participating in the study. I do not have to give a reason for this.
- I give permission for collecting and using my data to answer the research question of this study.
- I know that some people may gain access to all of my data for the purpose of checks of this study. Those people are listed in this information letter. I give permission for this access by these people.
- I wish to participate in this study.

Name of participant:

Email address:

Phone number:

Signature of participant: Date: __ / __ / __

The researcher, Lonneke Lenferink, hereby declares that the test subject has been fully informed about the aforementioned study.

If information becomes known during the study which might influence the consent of the participant, the participant will be timely informed about this.

For peer review only

Reporting checklist for protocol of a clinical trial.

Based on the SPIRIT guidelines.

Instructions to authors

Complete this checklist by entering the page numbers from your manuscript where readers will find each of the items listed below.

Your article may not currently address all the items on the checklist. Please modify your text to include the missing information. If you are certain that an item does not apply, please write "n/a" and provide a short explanation.

Upload your completed checklist as an extra file when you submit to a journal.

In your methods section, say that you used the SPIRITreporting guidelines, and cite them as:

Chan A-W, Tetzlaff JM, Altman DG, Laupacis A, Gøtzsche PC, Krleža-Jerić K, Hróbjartsson A, Mann H, Dickersin K, Berlin J, Doré C, Parulekar W, Summerskill W, Groves T, Schulz K, Sox H, Rockhold FW, Rennie D, Moher D. SPIRIT 2013 Statement: Defining standard protocol items for clinical trials. Ann Intern Med. 2013;158(3):200-207

		Reporting Item	Page Number
**Administrative information**			
Title	#1	Descriptive title identifying the study design, population, interventions, and, if applicable, trial acronym	1

For peer review only

Trial registration	#2a	Trial identifier and registry name. If not yet registered, name of intended registry	3
Trial registration: data set	#2b	All items from the World Health Organization Trial Registration Data Set	n/a
Protocol version	#3	Date and version identifier	n/a
Funding	#4	Sources and types of financial, material, and other support	32
Roles and responsibilities: contributorship	#5a	Names, affiliations, and roles of protocol contributors	32
Roles and responsibilities: sponsor contact information	#5b	Name and contact information for the trial sponsor	n/a
Roles and responsibilities: sponsor and funder	#5c	Role of study sponsor and funders, if any, in study design; collection, management, analysis, and interpretation of data; writing of the report; and the decision to submit the report for publication, including whether they will have ultimate authority over any of these activities	32
Roles and responsibilities: committees	#5d	Composition, roles, and responsibilities of the coordinating centre, steering committee, endpoint adjudication committee, data management team, and	n/a

For peer review only

		other individuals or groups overseeing the trial, if applicable (see Item 21a for data monitoring committee)	
**Introduction**			
Background and rationale	#6a	Description of research question and justification for undertaking the trial, including summary of relevant studies (published and unpublished) examining benefits and harms for each intervention	4-7
Background and rationale: choice of comparators	#6b	Explanation for choice of comparators	4-7
Objectives	#7	Specific objectives or hypotheses	8
Trial design	#8	Description of trial design including type of trial (eg, parallel group, crossover, factorial, single group), allocation ratio, and framework (eg, superiority, equivalence, non-inferiority, exploratory)	8-9
**Methods: Participants, interventions, and outcomes**			
Study setting	#9	Description of study settings (eg, community clinic, academic hospital) and list of countries where data will be collected. Reference to where list of study sites can be obtained	10

For peer review only

Eligibility criteria	#10	Inclusion and exclusion criteria for participants. If applicable, eligibility criteria for study centres and individuals who will perform the interventions (eg, surgeons, psychotherapists)	11
Interventions: description	#11a	Interventions for each group with sufficient detail to allow replication, including how and when they will be administered	12-13
Interventions: modifications	#11b	Criteria for discontinuing or modifying allocated interventions for a given trial participant (eg, drug dose change in response to harms, participant request, or improving / worsening disease)	19
Interventions: adherance	#11c	Strategies to improve adherence to intervention protocols, and any procedures for monitoring adherence (eg, drug tablet return; laboratory tests)	10
Interventions: concomitant care	#11d	Relevant concomitant care and interventions that are permitted or prohibited during the trial	16-17
Outcomes	#12	Primary, secondary, and other outcomes, including the specific measurement variable (eg, systolic blood pressure), analysis metric (eg, change from baseline, final value, time to event), method of aggregation (eg, median, proportion), and time point for each outcome. Explanation of the clinical relevance of chosen efficacy and harm outcomes is strongly recommended	14-17

For peer review only

Participant timeline	#13	Time schedule of enrolment, interventions (including any run-ins and washouts), assessments, and visits for participants. A schematic diagram is highly recommended (see Figure)	10
Sample size	#14	Estimated number of participants needed to achieve study objectives and how it was determined, including clinical and statistical assumptions supporting any sample size calculations	11
Recruitment	#15	Strategies for achieving adequate participant enrolment to reach target sample size	10
**Methods: Assignment of interventions (for controlled trials)**			
Allocation: sequence generation	#16a	Method of generating the allocation sequence (eg, computer-generated random numbers), and list of any factors for stratification. To reduce predictability of a random sequence, details of any planned restriction (eg, blocking) should be provided in a separate document that is unavailable to those who enrol participants or assign interventions	8-9
Allocation concealment mechanism	#16b	Mechanism of implementing the allocation sequence (eg, central telephone; sequentially numbered, opaque,	8-9

For peer review only

		sealed envelopes), describing any steps to conceal the sequence until interventions are assigned	
Allocation: implementation	#16c	Who will generate the allocation sequence, who will enrol participants, and who will assign participants to interventions	9
Blinding (masking)	#17a	Who will be blinded after assignment to interventions (eg, trial participants, care providers, outcome assessors, data analysts), and how	9
Blinding (masking): emergency unblinding	#17b	If blinded, circumstances under which unblinding is permissible, and procedure for revealing a participant's allocated intervention during the trial	n/a
**Methods: Data collection, management, and analysis**			
Data collection plan	#18a	Plans for assessment and collection of outcome, baseline, and other trial data, including any related processes to promote data quality (eg, duplicate measurements, training of assessors) and a description of study instruments (eg, questionnaires, laboratory tests) along with their reliability and validity, if known. Reference to where data collection forms can be found, if not in the protocol	14-17

For peer review only

Data collection plan: retention	#18b	Plans to promote participant retention and complete follow-up, including list of any outcome data to be collected for participants who discontinue or deviate from intervention protocols	n/a
Data management	#19	Plans for data entry, coding, security, and storage, including any related processes to promote data quality (eg, double data entry; range checks for data values). Reference to where details of data management procedures can be found, if not in the protocol	19
Statistics: outcomes	#20a	Statistical methods for analysing primary and secondary outcomes. Reference to where other details of the statistical analysis plan can be found, if not in the protocol	17-20
Statistics: additional analyses	#20b	Methods for any additional analyses (eg, subgroup and adjusted analyses)	17-20
Statistics: analysis population and missing data	#20c	Definition of analysis population relating to protocol non-adherence (eg, as randomised analysis), and any statistical methods to handle missing data (eg, multiple imputation)	17-20

Methods: Monitoring

Data monitoring: formal committee	#21a	Composition of data monitoring committee (DMC); summary of its role and reporting structure; statement of whether it is independent from the sponsor and competing interests; and reference to where further	19

For peer review only

		details about its charter can be found, if not in the protocol. Alternatively, an explanation of why a DMC is not needed	
Data monitoring: interim analysis	#21b	Description of any interim analyses and stopping guidelines, including who will have access to these interim results and make the final decision to terminate the trial	19
Harms	#22	Plans for collecting, assessing, reporting, and managing solicited and spontaneously reported adverse events and other unintended effects of trial interventions or trial conduct	19
Auditing	#23	Frequency and procedures for auditing trial conduct, if any, and whether the process will be independent from investigators and the sponsor	19
**Ethics and dissemination**			
Research ethics approval	#24	Plans for seeking research ethics committee / institutional review board (REC / IRB) approval	19
Protocol amendments	#25	Plans for communicating important protocol modifications (eg, changes to eligibility criteria, outcomes, analyses) to relevant parties (eg, investigators, REC / IRBs, trial participants, trial registries, journals, regulators)	19

For peer review only

Consent or assent	#26a	Who will obtain informed consent or assent from potential trial participants or authorised surrogates, and how (see Item 32)	19
Consent or assent: ancillary studies	#26b	Additional consent provisions for collection and use of participant data and biological specimens in ancillary studies, if applicable	n/a
Confidentiality	#27	How personal information about potential and enrolled participants will be collected, shared, and maintained in order to protect confidentiality before, during, and after the trial	19
Declaration of interests	#28	Financial and other competing interests for principal investigators for the overall trial and each study site	32
Data access	#29	Statement of who will have access to the final trial dataset, and disclosure of contractual agreements that limit such access for investigators	19
Ancillary and post trial care	#30	Provisions, if any, for ancillary and post-trial care, and for compensation to those who suffer harm from trial participation	19
Dissemination policy: trial results	#31a	Plans for investigators and sponsor to communicate trial results to participants, healthcare professionals, the public, and other relevant groups (eg, via publication, reporting in results databases, or other data sharing arrangements), including any publication restrictions	19-20

For peer review only

Dissemination policy: authorship	#31b	Authorship eligibility guidelines and any intended use of professional writers	n/a
Dissemination policy: reproducible research	#31c	Plans, if any, for granting public access to the full protocol, participant-level dataset, and statistical code	n/a

Appendices

Informed consent materials	#32	Model consent form and other related documentation given to participants and authorised surrogates	10
Biological specimens	#33	Plans for collection, laboratory evaluation, and storage of biological specimens for genetic or molecular analysis in the current trial and for future use in ancillary studies, if applicable	n/a

None The SPIRIT checklist is distributed under the terms of the Creative Commons Attribution License CC-BY-ND 3.0. This checklist can be completed online using https://www.goodreports.org/, a tool made by the EQUATOR Network in collaboration with Penelope.ai

For peer review only
